# Loneliness predicts a preference for larger interpersonal distance within intimate space

**DOI:** 10.1371/journal.pone.0203491

**Published:** 2018-09-06

**Authors:** Elliot A. Layden, John T. Cacioppo, Stephanie Cacioppo

**Affiliations:** 1 Department of Psychology, The University of Chicago, Chicago, IL, United States of America; 2 Center for Cognitive and Social Neuroscience, The University of Chicago, Chicago, IL, United States of America; 3 Department of Psychiatry and Behavioral Neuroscience, The University of Chicago Pritzker School of Medicine, Chicago, IL, United States of America; Middlesex University, UNITED KINGDOM

## Abstract

Loneliness is thought to serve as an adaptive signal indicating the need to repair or replace salutary social connections. Accordingly, loneliness may influence preferences for interpersonal distance. If loneliness simply motivates a desire to socially reconnect, then loneliness may be associated with a preference for smaller interpersonal distances. According to the evolutionary model of loneliness, however, loneliness also signals an inadequacy of mutual aid and protection, augmenting self-preservation motives. If loneliness both increases the motivation to reconnect and increases the motivation for self-protection, then the resulting approach-avoidance conflict should produce a preference for larger interpersonal distance, at least within intimate (i.e., proximal) space. Here, we report two survey-based studies of participants’ preferences for interpersonal distance to distinguish between these competing hypotheses. In Study 1 (*N* = 175), loneliness predicted preferences for larger interpersonal distance within intimate space net gender, objective social isolation, anxiety, depressive symptomatology, and marital status. In Study 2 (*N* = 405), we replicated these results, and mediation analyses indicated that measures of social closeness could not adequately explain our findings. These studies provide compelling evidence that loneliness predicts preferences for larger interpersonal distance within intimate space, consistent with predictions from the evolutionary model of loneliness.

## Introduction

Loneliness, defined as a discrepancy between one’s desired and achieved levels of social connectedness [1], has been found to impact behavioral processes related to attention, executive function, and social cognition (for review, see [2]). Recently, it has been hypothesized that loneliness may also influence preferences for interpersonal distance, that is, the preferred space between people in proximal space [3,4]. Interpersonal distance corresponds to "an area with an invisible boundary surrounding a person's body, into which intruders may not come" [5] (see also [6]). In Hall’s 1966 description of interpersonal distance, the space surrounding an individual’s physical body includes four main spheres/circles: 1) Intimate circle (0–18 in.; 0–45 cm), which corresponds to the distance closely surrounding a person’s body, wherein one feels comfortable interacting with intimate others; 2) Personal circle (18–47 in.; 45–120 cm), in which “subjects of personal interest and involvement can be discussed” [7]; 3) Social circle (47–142 in.; 120–360 cm), a zone used for impersonal business, interactions with colleagues, and casual social gatherings [7]; and 4) Public circle (> 142 in.; > 360 cm; Hall, 1963). Notably, these regions are similar both for physical and mental representations of the space surrounding one’s body [4,8–10].

The extant research on interpersonal distance, originally derived from Hall’s theory of proxemics, suggests that various factors can influence people’s preferences. For instance, preferences for interpersonal distance are strongly influenced both by feelings of social closeness [6], with preferences for smaller distances associated with greater intimacy [7,11], and by the defensive avoidance of social threats, with preferences for larger distances serving as a protective “body-buffer zone” [12]. Interestingly, the strength of avoidance motives increases more markedly with physical nearness than the strength of approach motives does [13–15]. Consistent with this finding, Hall (1969) described the intimate space as the most guarded, with only emotionally close individuals permitted.

Some gender differences in interpersonal distance have also been identified. For instance, females have been found to prefer smaller interpersonal distances than males [16], but Heshka and Nelson (1972) reported that specifically close female friends stood closer together than male dyads, whereas female strangers tended to stand at a larger distance than male dyads [17]. Heshka and Nelson (1972) explained this finding as due to the caution and reserve that females felt towards each other until a relationship has been established.

In the evolutionary model of loneliness, Cacioppo and colleagues [18–20] depict loneliness as an aversive signal that alerts individuals of the need to attend to and repair or replace the salutary connections with others that form the basis for cooperation and mutual aid and protection. This evolutionary model is not unique in predicting that loneliness motivates individuals to attend to or approach others. Gardner, Pickett, Jefferis, and Knowles (2005), for instance, found that lonelier participants exhibit significantly better recall of interpersonal and collective social events, are more accurate in identifying facial emotional expressions, and display greater attention to vocal tone in an emotional Stroop task [21]. These results were interpreted to suggest that lonely individuals engage in heightened social monitoring, which involves the selective biasing of attention and memory toward social cues during a state of “social hunger” [21,22]. This is thought to be analogous to the biasing of motivation, attention, and memory toward food cues during a state of physical hunger [23].

However, Cacioppo and colleagues’ evolutionary model additionally holds that loneliness is associated with a set of motivational, behavioral and cognitive adjustments related to short-term self-preservation [18,19]. Among these adjustments are an increase in self-centeredness [3] and an increase in implicit hypervigilance for social threats [24–26]. Intra-species aggression and violence feature as prominent threats to reproductive success among many social animals, and this is perhaps particularly characteristic of humans [27,28]. An unfettered motivation to approach and connect with others may meet with perilous consequences at the hands of hostile or duplicitous conspecifics. Therefore, the evolutionary model predicts that loneliness activates conflicting motivations, a motivation to approach to repair or replace tattered bonds in the service of long-term self-preservation, and a motivation to withdraw from and be alert for potential social threats in the service of short-term self-preservation. Evidence that loneliness is related to hypervigilance for social threats has been provided by studies utilizing functional neuroimaging [29–31], electrical neuroimaging [25,26], eye tracking [32–34], and behavioral techniques [24].

If loneliness simply motivates a desire to socially reconnect, then it should be associated with a motivation to approach, producing a preference for smaller interpersonal distance [11,35]. However, if loneliness also increases a self-protective hypervigilance for social threats, the resulting approach-avoidance conflict should produce a preference for larger interpersonal distance. If so, avoidance-related distancing should be particularly evident within intimate space, given that the strength of avoidance motives increases more markedly with physical nearness than the strength of approach motives [13–15]. Consistent with such an approach-avoidance conflict, behavioral research using a nonhuman primate model of loneliness has characterized lonely rhesus monkeys as showing more walkbys [36]. Specifically, the monkeys would approach other adult monkeys, walk within arms distance, and then walk away without interacting. Although the results of this animal study are suggestive, Studies 1 and 2 were designed test these hypotheses in humans [4,9,10].

## Study 1

To distinguish between our two competing hypotheses, we measured participants’ preferences for interpersonal distance within three regions of personal space that correspond to the three facets of loneliness: (1) intimate space–the personal space in which an individual is comfortable interacting with intimate others such as partners; (2) relational space–the personal space in which an individual is comfortable interacting with relational others such as friends; and (3) collective space–the personal space in which an individual is comfortable interacting with others who share a social identity [4]. We have previously found that intimate loneliness is associated (inversely) with marital status, that relational loneliness is associated with frequency of contact with friends and family, and that collective loneliness is associated with the number of voluntary groups to which one belongs [37,38]. To assure that participants conceptually understood these three interpersonal spaces, they were asked to list the relationship types of individuals with whom they preferred to interact in each space. Additionally, to investigate the specificity of association between loneliness and preferences for interpersonal distance, measures of objective social isolation, anxiety, depressive symptomatology, and marital status served as covariates. Loneliness, gender, personal space, and these covariates, were entered into a data-driven stepwise model selection procedure to determine which, if any, significantly predicted preferences for interpersonal distance.

### Materials and methods

This study was approved by the University of Chicago Social and Behavioral Sciences Institutional Review Board (IRB14-0649-CR002), and all participants provided written informed consent prior to participation. 233 international participants (175 US-based) completed a SurveyMonkey survey distributed via a co-author S. C.’s email-based social network during an approximately one-month period (June-July 2014). We restricted our main analyses to the 175 US-based respondents (111 women, 64 men, *M*_age_ = 44.0 years, age range: 19–83 years), due to the small numbers of respondents from any given foreign country and previously detailed cultural differences in preferences for interpersonal distance [7]. Notably, however, our main results were robust to this decision (see Section A in S1 File). Demographic information, including age, gender, ethnicity, income, education level, and marital/cohabitation status, was collected. Participants were instructed to respond to a number of questionnaires and behavioral measures, described below. A PDF of the survey is available (S1 Survey), and demographic information regarding participants in Study 1 is summarized in Table A in S1 File.

If any key behavioral (loneliness, objective isolation, depressive symptomatology, anxiety) or demographic (gender, marital status) variable was missing for a given participant, data for that participant were not included in the analyses. If key behavioral and demographic data were all present for a given participant, but some observations were missing for preferred interpersonal distance, the latter were treated as missing observations in the analyses. This criterion was used to ensure that all regression models incorporated the same participants, an important prerequisite for valid model comparisons during stepwise model selection (see below). This resulted in the removal of data for 19 of the 175 US-based participants (10.9%), yielding a final sample of 156 participants (101 women, 55 men, *M*_age_ = 44.4 years, age range: 19–83 years). Further details regarding the treatment of missing data are provided in Section B in S1 File.

#### Interpersonal distance preference

The expressed preference for interpersonal distance served as the criterion variable. Interpersonal distance preference was rated once for each personal space (intimate, relational, collective) and for strangers. Each personal space was described as follows: (1) intimate space–“My intimate circle includes people I consider intimate (e.g., myself, the love of my life), people in whom I can confide and who can confide in me, or people whose love means everything to me”; (2) relational space–“My relational circle includes people I consider as being a part of my life, people who are friends or family members that I can trust for protection and assistance, and who can similarly trust me”; (3) collective space–“My collective circle includes ‘social identities’ or groups with which I identify, such as a sport team, an occupational association, religious group, social group, or political party with which I identify. I feel connected to the people in this sector because of a shared group identity”; and (4) strangers–“People I do not consider friends or part of any group with which I may identify”. The preference for interpersonal distance for strangers served as a reference/control group to be contrasted with preferences within each of the aforementioned personal spaces.

Next, participants were asked the following question: “If a member in your ______ [Intimate, Relational, Collective, or Stranger] circle were to be sitting or standing next to you, how close would that individual have to get to make you feel uncomfortable?” The response scale was ordinal, with response options of, “No distance would make me feel uncomfortable,” “1–9 inches would begin to feel uncomfortable,” “10–25 inches would begin to feel uncomfortable,” “2.5 feet would begin to feel uncomfortable,” “5 feet would begin to feel uncomfortable,” “More than 5 feet would begin to feel uncomfortable,” “I have no one in my ______ [Intimate, Relational, Collective, or Stranger] circle,” and “Prefer not to answer” [39]. Responses to these questions corresponded to ordinal values of 1 (“No distance”), 2 (“1–9 inches”), 3 (“10–25 inches”), and 4 (“2.5 feet or greater”). Responses of “I have no one in my circle” and “prefer not to answer” were treated as missing observations. Importantly, these ordinal response options were chosen to coincide with dissociations of perceptual and representational space evidenced by neurological lesion case studies [8–10]. This evidence, largely in line with Hall (1966)’s divisions of interpersonal distance [24], suggests that interpersonal distance may be appropriately conceived of as a set of ordered zones or layers. In cases in which a construct is divisible into a small set of meaningful categories, an ordinal measure may provide more reliable and valid measurement of the construct than a continuous measure which seeks to maximize fine distinctions between levels [40–42]. However, to assure that this methodological choice did not somehow bias our results, we also included a sliding-scale measure of interpersonal distance in Study 2 below. With this continuous measure, we obtained results that were largely consistent with our primary ordinal measure; additionally, our main findings in Study 1 were robust to treating our ordinal interpersonal measure as continuous (see Section C in S1 File).

#### Relationship types

To validate that participants understood these personal spaces, we asked participants to select the relationship types of individuals with whom they preferred to interact in each space, as well as how many individuals they included in each. For the intimate and relational spaces, options included “Romantic Partner,” “Grand Parent(s),” “Mother,” “Father,” “Brother(s),” “Sister(s),” “Best Friend(s),” or “Other.” For the collective space, which is primarily related to collective identities and groups as opposed to individuals [37], options included, “Sport Team(s),” “Club(s),” “Professional Association(s),” “Committee(s),” “Religious Group(s),” “Nation(s),” or “Other.”

The relationship types specified for each personal space suggested that the participants understood the distinctions among the personal spaces when expressing their preferences for interpersonal distance. For instance, “Best Friend(s)” (61.5%) and “Romantic Partner” (59.6%) were among the most cited relationship types included in intimate space. “Best Friend(s)” (71.8%) and various family members, including “Brother(s)” (29.5%), “Mother” (25.0%), “Father” (25.0%), and “Sister(s)” (24.4%), were the most commonly cited types for relational space. Finally, “Professional Association(s)” (57.7%) and “Club(s)” (38.5%) were the most commonly cited group types for collective space. Across personal spaces, the only relationship type that differed between lonelier and less lonely participants (median split) was “Romantic Partner” within the intimate space. 51.4% of lonelier individuals included this relationship type, compared to 70.9% of less lonely individuals (χ^2^ = 6.46, *p* = .011). Table B in S1 File displays the number and percentage of participants who specified given relationship types for each personal space.

#### Loneliness

Loneliness was assessed using a 9-item version of the revised UCLA Loneliness Scale [37]. An example item is, “How often do you feel that there is no one you can turn to?” Each item is rated on a 4-point Likert-type scale: never (1), rarely (2), sometimes (3), or often (4). Total loneliness scores were calculated by summing across all 9 items (*α* = .88), reverse coding where necessary so that higher scores corresponded to greater loneliness (*M* = 15.54, *SD* = 4.62, sample range: 9–30).

#### Additional covariates

Gender, marital/cohabitation status, objective social isolation, anxiety, and depressive symptomatology were measured and served as covariates (see “Data analyses,” below). *Marital/cohabitation status* was quantified by coding a participant as married/cohabiting if the participant was legally married, in a civil union, or cohabiting. A participant was coded as non-married/not cohabiting if the participant was single, divorced, separated, or widowed. By this criterion 52.6% of participants were considered married/cohabiting. Marriage and female gender predict smaller interpersonal distances [43], so both variables served as predictors.

Objective social isolation was gauged using the scale reported in [44]. This measure asks respondents if they are unmarried/not cohabiting; have less than monthly contact with each of children, other family members, and friends; and if they participate in organizations such as social clubs, religious groups, or committees. To calculate total scores (possible range: 0–5), one point was added for each form of isolation indicated by a respondent (*M* = 1.44, *SD* = 1.03, sample range: 0–4).

Anxiety was measured using the Hospital Anxiety and Depression Scale [45], a validated self-report measure. Responses to the seven anxiety items were scored from 0 to 3, reverse scoring where necessary so that higher scores correspond to greater anxiety (*α* = .80, *M* = 5.65, *SD* = 3.64, sample range: 0–16). Trait anxiety is associated with both loneliness and preferences for interpersonal distance [46,47], so this variable served as a covariate.

Finally, depressive symptomatology was also measured using the HADS. The HADS contains seven items relating to depressive symptomatology, scored from 0–3. These were reverse scored where necessary so that higher scores corresponded to higher depressive symptomatology *α =* .78, *M* = 3.24, *SD* = 3.00, sample range: 0–15). Loneliness and depressive symptomatology are related [48], but the association between depressive symptomatology and preferences for interpersonal distance has not been determined. Nevertheless, this variable served as a covariate to ensure any associations between loneliness and preferences for interpersonal distance were not attributable to depressive symptomatology.

#### Data analyses

Due to the ordinal nature of the preference ratings and the presence of repeated measures, we implemented proportional odds mixed models to test our main hypotheses [49,50] using the “ordinal” package in R [51]. For an ordinal outcome measure, the proportional odds model offers higher statistical efficiency than binary logistic regression based on a dichotomization of the ordinal scale; additionally, this model avoids issues often encountered in cases wherein an ordinal outcome is assumed to have the properties of an interval scale (e.g., violation of the variance homogeneity assumption and/or heteroscedasticity of residuals) [52].

The proportional odds model splits the ordinal criterion variable into a sequence of dichotomies or “cut-points,” with one fewer cut-point than the number of categories. Inspection of the response distribution for the rated preferences for interpersonal distance revealed that only two participants selected response categories greater than “10–25 inches” for intimate space, so we collapsed the preference ratings into the following three ordered categories for all analyses in Study 1: “No distance,” “1–9 inches,” and “10–25 inches or greater.” Therefore, the three response categories for our criterion variable were (a) no-distance, (b) 1–9 in, and (c) equal to or greater than 10 in. The first cut-point was between (a) and (b), and the second cut-point was between (b) and (c). At each cut-point, the logit, $\log\left(\frac{P(Y\geq j)}{P(Y<j)}\right),$ is modelled, wherein *j* is an ordered category of the criterion variable, *Y*. That is, the model predicts the log odds of a response being equal to or greater than each cut-point. A distinct threshold or intercept term could be fitted for each cut-point of the ordinal criterion variable, but the proportional odds assumption means that parameter estimates for a given predictor are constant across cut-points [49]. Thus, parameter estimates are obtained by maximizing the likelihood function across all cut-points. This procedure yields cumulative odds ratios that are independent of the specific cut-points used to dichotomize the ordinal criterion variable, so long as the proportional odds assumption is valid [52].

Maximum likelihood parameter estimates were obtained via adaptive Gauss-Hermite quadrature approximation using 11 quadrature points [53,54], implemented using the nlminb function in R 3.2.3 (R Foundation for Statistical Computing, www.rproject.org). A random intercept was included for each subject, accounting for repeated measurements of the criterion variable. Personal space (intimate, relational, collective), loneliness, gender, marital status, objective social isolation, anxiety, and depressive symptomatology were entered into a data-driven stepwise (forward-selection) procedure to determine which, if any, significantly predicted preferences for interpersonal distance [55]. Importantly, loneliness competed with the remaining factors for entry into the model in an experimenter-blind, data-driven fashion, thereby reducing experimenter degrees-of-freedom for model specification.

Beginning from an intercept-only model, at each step, we selected the predictor that yielded the largest, significant increase in model log-likelihood. For each candidate predictor, we then tested whether the proportional odds assumption was met by computing the likelihood-ratio test between two candidate models, one in which the predictor was entered as an ordinal effect (slope assumed equal across cut-points) and one in which the predictor was entered as a nominal effect (slope allowed to vary across cut-points) [51]. If ordinal and nominal effects were not significantly different, the more parsimonious ordinal effect was opted for and entered into the model. Next, we tested whether the inclusion of an interaction between the predictor and personal space significantly improved the model fit. If so, this interaction was also included in the model. As the proportional odds case (all effects are ordinal) and the partial proportional odds case (some effects are nominal) both represent sub-models of (i.e. are nested within) the non-proportional odds case, the likelihood-ratio test represents a valid method for comparing otherwise equivalent models fitted using these differing assumptions [56]. Furthermore, step-up model selection was deemed preferable to an alternative step-down selection procedure due to the unwieldiness of the latter when multiple variables are considered [55]. A full description of the model selection steps and relevant statistics are provided in Table C in S1 File.

### Results

Stepwise selection indicated that the optimal model for predicting interpersonal distance preferences included the following predictors, added in this order: (1) personal space dimension, (2) loneliness, (3) a loneliness*personal space dimension interaction, and (4) gender. As expected, preferences for interpersonal distance increased markedly from proximal to distal personal space dimensions: intimate space (*M* = 1.23, *SD* = .59), relational space (*M* = 1.86, *SD* = .90, collective space (*M* = 2.57, *SD* = .93), and stranger (*M* = 3.28, *SD* = .97). Critically, the loneliness*personal space interaction indicated that lonelier participants preferred significantly larger interpersonal distance within their intimate space (*B* = .96, *Z* = 2.10, *p* = .035, *OR* = 2.62, *CI* = [1.07, 6.44]). In contrast, loneliness was not associated with preferences for relational (*B* = .25, *Z* = .66, *p* > .5) or collective interpersonal distance (*B* = -.11, *Z* = -.29, *p* > .75) (Table 1). We also noted that male, compared to female, participants expressed preferences for larger interpersonal distance across personal spaces (*B* = 1.18, *Z* = 2.47, *p* = .014, *OR* = 3.25, *CI* = [1.27, 8.28]). As a whole, the model was significant compared to an intercept-only model (*χ*^*2*^ (8) = 444.93, *p* < .001; *McFadden R*^*2*^ = .35; *adjusted McFadden R*^*2*^ = .34), with *McFadden’s R*^*2*^ [57] and *Count R*^*2*^ [58] both indicating an excellent fit (386/591 = 65.31% of interpersonal distance ratings were predicted correctly; *adjusted Count R*^*2*^ = .41). Coefficient estimates, standard errors, and odds ratios for all predictors in the model are displayed in Table 1.

**Table 1:** Study 1 final model.

Covariate	*B* (*SE B*)	*Z* (*p*)	*OR* (95% *CI*)
**Personal Space**			
Intimate	-8.27 (.684)	-12.09 (< .001)	< .01 (< .01 - < .01)
Relational	-4.70 (.453)	-10.37 (< .001)	.01 (< .01 - .02)
Collective	-2.21 (.363)	-6.08 (< .001)	.11 (.05 - .22)
**Psychosocial**			
Loneliness	.022 (.376)	0.06 (.95)	1.02 (.49–2.14)
Loneliness*Intimate	.964 (.458)	2.10 (.035)	2.62 (1.07–6.44)
Loneliness*Relational	.251 (.377)	0.66 (.51)	1.28 (.61–2.69)
Loneliness*Collective	-.108 (.371)	-.29 (.77)	.90 (.43–1.86)
**Demographic**			
Gender: Male	1.18 (.478)	2.47 (.014)	3.25 (1.27–8.28)
**Thresholds**			
1|2	-4.85 (.498)	-9.73 (< .001)	.01 (< .01 - .02)
2|3	-2.18 (.394)	-5.54 (< .001)	.11 (.05 - .24)
	***SD***	***95% CI***	
Random Intercept (Subj.)	2.36	(1.84, 2.97)	-
**Model Fit**			
AIC	841.04		
Log-Likelihood	-409.52		
Adjusted McFadden R^2^	0.34		
Count R^2^	0.65		
Adjusted Count R^2^	0.41		

*Note*. *The 95% confidence interval for the standard deviation of the random intercept was determined using the profile likelihood method*.

*Note 2*. *No row for strangers appears because strangers served as the reference category for the nominal personal space dimension variable*.

#### Controlling for social network characteristics

We considered the possibility that lonelier individuals might have zero or fewer individuals within their intimate space, something that might make estimating interpersonal distance within intimate space difficult. As expected, there was a small negative correlation between loneliness and the number of members participants included within their intimate spaces (*r*(169) = -0.29, *p* < 0.001), yet those in the 75^th^ percentile of loneliness scores still included an average of 3.4 individuals within their intimate space. We added the number of individuals in personal space dimensions as a factor in our proportional odds mixed model and found that it was not significantly associated with interpersonal distance preferences (*B* = .02, *Z* = 1.42, *p* > .15), whereas loneliness remained a significant predictor of interpersonal distance preference within intimate space (*B* = 2.62, *Z* = 2.49, *p* = .013) when controlling for this factor. In a follow-up regression, we also coded an interaction between number of individuals and personal space dimension. However, no simple effects related to number of individuals were significant (all *p*’s > .38), whereas loneliness still predicted interpersonal distance preference within intimate space (*p* = .013). Finally, for the sake of thoroughness, we coded a three-way interaction between personal space dimension, loneliness, and number of individuals. Again, no simple effects related to number of individuals were significant (all *p*’s > .46), whereas loneliness still significantly predicted interpersonal distance preference within intimate space (*p* = .044).

#### The effect of marriage

We also considered the possibility that married individuals might prefer less interpersonal distance within intimate space, given that one’s spouse would likely be included as a member of intimate space. Notably, marital status was a candidate variable for stepwise model selection but was not added by our automated procedure, meaning that its inclusion did not significantly improve the model. Nevertheless, we added marital status to our final model to test whether the inclusion of this variable affected the association observed between loneliness and interpersonal distance preferences within intimate space. Marital status was not a significant predictor of interpersonal distance preference in this model (*B* = -.70, *Z* = -1.55, *p* = .12), and loneliness still predicted preferences for larger interpersonal distance within intimate space (*B* = 95, *Z* = 2.08, *p* = .038). Additionally, we found no evidence to suggest an interaction between marital status and personal space dimension (all *p*’s > .18), nor a three-way interaction between marital status, personal space dimension, and loneliness (all *p*’s > .18).

### Discussion

Our aim in Study 1 was to test competing hypotheses about the association between loneliness and personal space, especially within intimate space. If loneliness simply motivates a desire to socially reconnect, analogous to the effects of hunger on food seeking, then we reasoned that loneliness should be associated with a preference for smaller interpersonal distance. However, if loneliness simultaneously augments a self-protective hypervigilance for social threats in the service of short-term self-preservation, the resulting approach-avoidance conflict should produce a preference for a larger interpersonal distance within intimate space, based upon the proximity gradients of approach-avoidance conflicts [13–15]. The results of Study 1 were consistent with the prediction from the evolutionary model [19,20] that loneliness would be associated with a preference for larger interpersonal distance within intimate space, and they were inconsistent with the notion that loneliness serves merely as a signal to reconnect with others. Additionally, the results of Study 1 replicated a gender difference that has been found consistently in the literature on personal space [59], specifically, that males tend to prefer larger interpersonal distances than females across personal spaces.

Importantly, our main result could not be accounted for by lonelier participants including fewer individuals within their intimate spaces, nor by the marital status of participants. Moreover, a measure of objective social isolation did not contribute to our model of interpersonal distance preferences. These results do not support the notion that objective or quantitative differences in personal space composition mediate lonelier participants’ preferences for larger intimate distance. However, these analyses do not rule out the possibility that differences in the *quality* of relationships with members of the intimate space might mediate the differences observed. Therefore, in Study 2, we introduced a set of multifaceted measures of relationship quality to gauge whether lonelier participants might prefer larger intimate distances as a result of relationships within their intimate spaces being of lesser quality.

## Study 2

We had two primary aims in Study 2: First, we sought to cross-validate the results of Study 1 by testing the final model derived in Study 1 in a larger sample. This cross-validation provides a strong test of the replicability of the association identified in Study 1 between loneliness and the preference for larger intimate interpersonal distance. Second, although we controlled for objective or quantitative differences in personal space composition in Study 1, here we also sought to determine the extent to which this association might be mediated by differences in relationship quality between lonelier and less lonely individuals. Specifically, we measured multiple facets of the psychological dimension of social closeness–a construct that is strongly associated with preferences for (smaller) interpersonal distance [6]. Social closeness is a manifold construct that consists of the dissociable factors of “feeling close” and “behaving close” [60]. To account for this diversity, we incorporated five measures spanning the construct of social closeness: (1) an index of subjective social closeness to gauge “feeling close” [60,61]; (2) preferences for more or less social closeness (closeness preference); (3) an index of frequency of contact, gauging the “behaving close” facet [60,61]; (4) preferences for more or less frequency of contact; and (5) the Inclusion of Other in the Self (IOS) Scale as an overall index of closeness, gauging the extent to which others are regarded as close to or overlapping with the self [60,62]. We then formally tested whether any of these variables mediated the observed association between loneliness and the preference for larger interpersonal distance within intimate space.

### Materials and method

This study was approved by the University of Chicago Social and Behavioral Sciences Institutional Review Board (IRB14-0649-CR002), and all participants provided written informed consent prior to participation. 520 participants attempted a Qualtrics-based survey (S2 Survey) distributed via Amazon Mechanical Turk (MTurk). MTurk is an online participant pool with similar validity and reliability characteristics to traditional subject pools [63–67] and comparable rates of problematic responding [68]. Participants were compensated $3 for the approximately 20–25 minute survey. We included a set of 10 attention checks to help assure that participants fully read the questions and did not rush excessively. We made the *a priori* decision to exclude data from any participants who answered fewer than 8 of the 10 attention checks correctly. The survey automatically tallied the number of attention checks missed in real-time and ejected participants from the survey if they missed three or more questions. Participants were explicitly informed of this compensation and attention check procedure within the written consent form prior to participating. Data for 88 participants were automatically excluded prior to completion of the survey for too many attention checks missed, data from 22 participants were excluded for attempting to take the survey multiple times for additional compensation (detected via duplicate MTurk IDs), data from 4 participants were excluded for providing invalid MTurk worker IDs, and data from 1 participant was automatically excluded for attempting the survey as a non-US resident. In total, 405 US-based participants (208 women, 197 men, *M*_age_ = 35.1 years, *SD* = 10.6) successfully completed the survey. Missing data from among these 405 participants were treated the same as in Study 1. Participants were instructed to respond to the same questionnaires and behavioral measures as used in Study 1, in addition to measures (described below) included to aid in the assessment of mediation of the main association of interest observed in Study 1 (i.e., between loneliness and intimate interpersonal distance preference).

#### Demographic and behavioral measures

Demographic information was gathered as described in Study 1, and sample demographic data for Study 2 are summarized in Table D in S1 File. The following behavioral scales from Study 1 were used in Study 2: UCLA loneliness scale (*α*_Study 2_ = .94, *M* = 16.78, *SD* = 6.29, sample range: 9–36), HADS anxiety scale (*α*_Study 2_ = .89, *M* = 4.98, *SD* = 4.37, sample range: 0–21), HADS depressive symptomatology scale (*α*_Study 2_ = .88, *M* = 4.25, *SD* = 4.22, sample range: 0–21), and the objective isolation measure (*M* = 1.78, *SD* = 1.14, sample range: 0–5). Unlike in Study 1, there was sufficient data available in Study 2 to utilize four response ranges for the interpersonal distance preference ratings (“No distance,” “1–9 inches,” “10–25 inches,” and “2.5 feet or greater”). All ordinal regression models therefore incorporated these four ordinal response categories.

#### Relationship types

As in Study 1, we asked participants to select the relationship types of individuals with whom they preferred to interact in each personal space to validate that they understood these personal spaces. Response options for all personal spaces included “Spouse or Partner,” “Parent(s),” “Grandparent(s),” “Sibling(s),” “Children,” “Grand Children,” “Other Relative(s),” “Close Friend,” “Friend(s),” “Acquaintance(s),” “Co-worker(s) or Colleague(s),” “Teammate(s),” “Club Member(s),” “Professional Association Member(s),” “Committee Member(s),” “Religious Organization Member(s),” “Classmate(s),” or “Leisure Activity Group Member(s).” The relationship types specified for each personal space suggested that participants understood the distinctions among the personal spaces when expressing their preference for interpersonal distance. For instance, “Romantic Partner” (62.7%), “Parent(s)” (53.3%), and “Close Friend(s)” (38.0%), were the most commonly cited relationship types for intimate space. “Close Friend(s)” (52.4%), “Friend(s)” (47.7%), and “Siblings” (31.9%) were the most commonly cited relationship types for relational space. “Colleague(s)” (27.7%), “Friend(s)” (21.2%), and “Acquaintance(s)” (20.0%) were the most commonly cited relationship types for collective space. Table E in S1 File displays the number and percentage of participants who specified given relationship types for each personal space.

#### Social/emotional closeness

As a putative mediator, we included a more direct measure of the cognitive and affective components of social closeness. This measure was implemented for each personal space by asking participants, “How close (psychologically / emotionally) do you feel to members of your ______ [Intimate, Relational, or Collective] circle on average?” Responses were made using a sliding-bar type question in Qualtrics, with ticks from “Least Close” (0) to “Closest” (10). “Strangers” was omitted as a target for these ratings due to potential confusion about what was meant by feelings of closeness toward this group. We also gauged the participants’ preference for social closeness with members of each personal space. This measure was implemented for each personal space by asking participants, “Considering your level of closeness to members of your _____ [Intimate, Relational, or Collective] circle, would you prefer to have:” Responses were made on a 5-point scale, from “Much less closeness” (-2) to “Much more closeness” (2). This item was also omitted for strangers.

We also implemented the IOS scale to pictorially measure participants’ feelings of self-other overlap. This scale was implemented by asking participants to “Please select the picture below that best describes your relationship with members of your _____ [Intimate, Relational, or Collective] circle on average:” Response options included a series of seven standard pairs of circles which overlap to greater or lesser degrees. This question was omitted for strangers.

#### Social/behavioral closeness

To assess feelings of behavioral closeness with others, we measured the participants’ frequency of contact with members of each personal space by asking, “How often do you have contact (face-to-face, telephone, written, online or via a monitor screen) with members of your ______ [Intimate, Relational, or Collective] circle on average?” Responses were made on a Likert-type scale from “Very Rarely” (1) to “Very Often” (5). We also asked participants about their preferences for more or less contact with members of their personal spaces: “Considering your amount of contact with your ______ [Intimate, Relational, or Collective] circle, would you prefer to have:” Responses were made on a Likert-type scale from “Much less contact” (-2) to “Much more contact” (2). These items were omitted for strangers.

#### Cross-validation of Study 1

To address our first aim, we used the final model identified by stepwise selection in Study 1, and we applied the proportional odds mixed model to determine the association between loneliness and preferences for interpersonal distance. Notably, to assure that the use of an ordinal measure of interpersonal distance preference did not somehow bias our results, we also included a sliding scale, continuous measure of interpersonal distance in Study 2. With this continuous measure, we obtained results that were consistent with those obtained using our primary ordinal measure in both studies 1 and 2 (see Section C in S1 File).

#### Mediation analysis

To address the second aim, we evaluated each of the various measures related to the construct of social closeness (social closeness, social closeness preference, IOS, frequency of contact, and frequency of contact preference) using the same stepwise model selection procedure as implemented in Study 1. This allowed us to separately test the proportional odds assumption for each measure, and to test for interactions with personal space. Only measures that met the criterion of statistical significance were included in the final model for Study 2 and served as potential mediators in subsequent analyses. As noted above, social closeness was not measured in relation to strangers to avoid confusion about what was meant by social closeness. Consequently, for mediation analyses, the collective personal space served as the reference category, rather than strangers. A full description of the model selection steps and relevant statistics are provided in Table F in S1 File.

Mediation was investigated using a non-parametric simulation-based method for proportional odds models [69,70], with implementation using custom R scripts based on the R package “mediation” [71], which is currently incompatible with “ordinal” package model types. This approach involves creating a bootstrap distribution of the natural direct effect (*NDE*) and the natural indirect effect [70], the latter of which is also known as the average causal mediation effect [71]. The *NIE*, or *ACME*, “represents the causal effect of the treatment on the outcome that can be attributed to the treatment-induced change in the mediator” when the treatment variable itself is held constant [72]. This approach has been generalized to multilevel modelling [71,73,74] and to ordinal outcome variables [70,71]. Importantly, we expected and confirmed moderate to strong associations among our putative mediators, even when controlling for loneliness. Thus, we utilized a methodology developed specifically for the case of multiple causally related mediators [72]. For further methodological details, see Section F in S1 File.

### Results

#### Cross-validation

We began by testing the consistency and replicability of the optimal model identified in Study 1 here in our larger Study 2 sample. Consistent with Study 1, preferences for interpersonal distance increased markedly from proximal to distal personal space dimensions: intimate space (*M* = 1.42, *SD* = .77), relational space (*M* = 2.22, *SD* = .89), collective space (*M* = 2.90, *SD* = .87), and stranger (*M* = 3.51, *SD* = .72). Importantly, we found that lonelier participants again preferred significantly larger interpersonal distances within intimate space (*B* = .69, *Z* = 3.68, *p* < .001, *OR* = 2.00, *CI* = [1.38, 2.89]), whereas loneliness was not associated with interpersonal distance preferences within relational (*B* = .11, *Z* = 0.66, *p* > .5) or collective space (*B* = .15, *Z* = 0.92, *p* > .75). The gender effect noted in Study 1 also replicated, with males preferring significantly larger interpersonal distances than females across social dimensions (*B* = .71, *Z* = 3.11, *p* = .002, *OR* = 2.03, *CI* = [1.30, 3.16]). As in Study 1, the model was significant compared to an intercept-only model (*χ*^*2*^ (8) = 1329.10, *p* < .001; *McFadden R*^*2*^ = .31; *adjusted McFadden R*^*2*^ = .31), with *McFadden’s R*^*2*^ and *Count R*^*2*^ both indicating an excellent fit (826/1,542 = 53.57% of observations were predicted correctly; *adjusted Count R*^*2*^ = .38). Coefficient estimates, standard errors, and odds ratios for all predictors in the model are displayed in Table 2.

**Table 2:** Study 2 replication of Study 1 final model.

Covariate	*B* (*SE B*)	*Z* (*p*)	*OR* (*95% CI*)
**Personal Space**			
Intimate	-7.59 (.308)	-24.64 (< .001)	< .01 (< .01 - < .01)
Relational	-4.36 (.214)	-20.36 (< .001)	.01 (< .01 - .02)
Collective	-2.24 (.177)	-12.66 (< .001)	.11 (.07 - .15)
**Psychosocial**			
Loneliness	.278 (.162)	1.72 (.086)	1.32 (.96–1.81)
Loneliness*Intimate	.693 (.188)	3.68 (< .001)	2.00 (1.38–2.89)
Loneliness*Relational	.108 (.164)	0.66 (.51)	1.11 (.81–1.54)
Loneliness*Collective	.154 (.168)	0.92 (.36)	1.17 (.84–1.62)
**Demographic**			
Gender: Male	.707 (.227)	3.11 (.002)	2.03 (1.30–3.16)
**Thresholds**			
1|2	-5.91 (.283)	-20.90 (< .001)	< .01 (< .01 - < .01)
2|3	-3.07 (.224)	-13.69 (< .001)	.05 (.03 - .07)
3|4	-.513 (.193)	-2.66 (.008)	.60 (.41 - .87)
	***SD***	***SD 95% CI***	
Random Intercept (Subj.)	1.95	(1.65, 2.31)	-
**Model Fit**			
AIC	2967.24		
Log-Likelihood	-1471.62		
Adjusted McFadden R^2^	0.31		
Count R^2^	0.54		
Adjusted Count R^2^	0.38		

#### Mediation analysis

All measures of social closeness were significantly associated with both interpersonal distance preferences and loneliness, in at least one or more personal space dimensions. Distributional information and statistics summarizing these associations are detailed in Table 3.

**Table 3:** Putative mediators.

Behavioral Variable	Mean (SD)	Loneliness (*r*)	IPD Preference (*Z*)
**Social Closeness**			
Intimate	8.85 (1.67)	-0.41 ***	-6.43 ***
Relational	5.94 (2.11)	-0.41 ***	-2.55 *
Collective	3.54 (2.40)	-0.15 **	-2.24 *
**Social Closeness Preference**			
Intimate	0.32 (0.58)	0.25 ***	0.01
Relational	0.37 (0.62)	0.13 **	-2.53 *
Collective	0.13 (0.53)	-0.02	-0.43
**IOS**			
Intimate	5.74 (1.34)	-0.33 ***	-2.46 *
Relational	3.75 (1.26)	-0.31 ***	-2.78 **
Collective	2.54 (1.20)	-0.21 ***	-2.23 *
**Frequency of Contact**			
Intimate	4.58 (0.80)	-0.33 ***	-2.31 *
Relational	3.57 (0.89)	-0.28 ***	-1.88 †
Collective	2.71 (1.18)	-0.19 ***	-2.12 *
**Frequency of Contact Preference**			
Intimate	0.39 (0.65)	0.21 ***	0.33
Relational	0.44 (0.64)	0.05	-2.53 *
Collective	0.20 (0.76)	-0.01	-2.15 *

*Note*. *IPD = Interpersonal Distance; Z values were obtained from regressing IPD preference onto each putative mediator in a proportional odds model*.

*†*, *, **, and ***, *correspond to p <* .*10*, *p <* .*05*, *p <* .*01*, *and p <* .*001*, *respectively*.

Stepwise model selection produced an optimal model consisting of the following predictors: (1) personal space dimension, (2) loneliness, (3) a personal space*loneliness interaction, (4) gender, (5) social closeness, (6) social closeness preference, (7) a personal space*social closeness preference interaction, (8) frequency of contact preference, and (9) self-other overlap. Because social closeness was not measured in regards to strangers (see Materials and methods), collective space served as the reference category for the personal space dimension variable in this regression model. Largely consistent with the Study 1 model, lonelier participants were marginally more likely to prefer larger interpersonal distances within intimate space (*B* = .36, *Z* = 1.91, *p* = .056, *OR* = 1.44, *CI* = [.99, 2.09]), but no association was noted within relational space (*B* = -.19, *Z* = -1.14, *p* > .2). Notably, the small reduction in effect size and corresponding change in *p*-level for the former effect may reflect the use of collective space rather than strangers as the reference category for this analysis. Despite the association between loneliness and interpersonal distance preference within intimate space being marginally significant in the presence of putative mediators, the overall loneliness*personal space interaction remained significant (Likelihood-ratio drop-test: *χ*^*2*^ (2) = 9.21, *p* = .01).

In contrast to previous analyses, loneliness exhibited a significant main effect in this model, predicting preferences for larger interpersonal distance across personal space dimensions (*B* = .34, *Z* = 2.09, *p* = .036, *OR* = 1.40, *CI* = [1.02, 1.93]). The model as a whole was significant compared to an intercept-only model (*χ*^*2*^ (16) = 821.44, *p* < .001; *McFadden R*^*2*^ = .27; *adjusted McFadden R*^*2*^ = .26), with *McFadden’s R*^*2*^ and *Count R*^*2*^ both indicating an excellent fit (668/1,150 = 58.09% of observations were predicted correctly; *adjusted Count R*^*2*^ = .37). Regression results are shown in Table 4.

**Table 4:** Regression results for Study 2 putative mediator model.

Covariate	*B* (*SE B*)	*Z* (*p*)	*OR* (*95% CI*)
**Personal Space**			
Intimate	-3.25 (.37)	-8.88 (< .001)	.04 (.02 - .08)
Relational	-1.35 (.22)	-6.17 (< .001)	.26 (.17 - .40)
**Demographic**			
Gender: Male	.68 (.25)	2.68 (.007)	1.97 (1.20–3.24)
**Psychosocial**			
Loneliness	.34 (.16)	2.09 (.036)	1.40 (1.02–1.93)
Loneliness*Int.	.36 (.19)	1.91 (.056)	1.44 (.99–2.09)
Loneliness*Rel.	-.19 (.16)	-1.14 (.256)	.83 (.60–1.14)
**Putative Mediators**			
Freq. Contact Pref.	-.33 (.16)	-2.07 (.038)	.72 (.53 - .98)
Closeness Pref.	.53 (.28)	1.90 (.057)	1.70 (.98–2.92)
Closeness Pref.*Int.	-.69 (.38)	-1.79 (.074)	.50 (.24–1.07)
Closeness Pref.*Rel.	-.74 (.31)	-2.34 (.019)	.48 (.26 - .89)
**Social Closeness**			
1|2	.41 (.08)	4.91 (< .001)	.67 (.57 - .78)
2|3	.21 (.07)	3.12 (.002)	.81 (.71 - .93)
3|4	.19 (.08)	2.46 (.014)	.83 (.71 - .96)
**IOS**			
1|2	.32 (.12)	2.73 (.006)	.72 (.57 - .91)
2|3	.26 (.12)	2.21 (.027)	.77 (.61 - .97)
3|4	-.08 (.14)	-.56 (.577)	1.08 (.82–1.43)
**Thresholds**			
1|2	-7.07 (.57)	-12.36 (< .001)	< .01 (< .01 - < .01)
2|3	-2.36 (.34)	-6.88 (< .001)	.09 (.05 - .18)
3|4	1.51 (.36)	4.14 (< .001)	4.5 (2.21–9.18)
	***SD***	***SD 95% CI***	
Random Intercept (Subj.)	2.11	(1.72, 2.58)	-
**Model Fit**		
AIC	2275.51	
Log-Likelihood	-1117.76	
Adj. McFadden R^2^	0.26	
Count R^2^	0.58	
Adjusted Count R^2^	0.37	

Next, we formally tested whether any measure of social closeness significantly mediated the association between loneliness and preference for interpersonal distance within intimate space. We separately examined each measure of social closeness as a putative primary mediator, utilizing an analysis strategy that allows a primary mediator to also act via indirect effects transferred through secondary mediators, in this case, other social closeness measures [72] (see Section F in S1 File). We performed omnibus tests of the natural indirect effect (*NIE-Q*) across levels of interpersonal distance preference and gender. The *NIE-Q* was not statistically significant for any measure of social closeness: (a) social closeness preference (*NIE-Q* = .000046, *p* = .36), (b) frequency of contact preference (*NIE-Q* = .00021, *p* = .077), (c) social closeness (*NIE-Q* = .00093, *p* = .10), or (d) the IOS (*NIE-Q* = .00021, *p* = .11) (Table 5). Additionally, no mediation effects were significant when tested separately within each gender: (a) social closeness (*NIE-Q* = .00028, *p* = .079 for females, *NIE-Q* = .00065, *p* = .11 for males), (b) closeness preference (*NIE-Q* = .000014, *p* = .36 for females, *NIE-Q* = .000032, *p* = .37 for males), (c) frequency of contact preference (*NIE-Q* = .00006, *p* = .061 for females, *NIE-Q* = .000014, *p* = .10 for males), or (d) the IOS (*NIE-Q* = .000065, *p* = .094 for females, *NIE-Q* = .00015, *p* = .14 for males). The right column of Table 5 shows that the inclusion of the social closeness mediators only nominally affected the natural direct effects modeled for loneliness.

**Table 5:** Omnibus test for mediation of the association between loneliness and interpersonal distance preference within intimate space.

Putative Mediator	*NIE-Q*	*p*	*NDE-Q*	*p*
Closeness Preference	4.60E-05	0.356	0.021	0.047
Freq. Contact Preference	2.10E-04	0.078	0.024	0.032
Social Closeness	0.00093	0.103	0.012	0.070
IOS	2.10E-04	0.105	0.016	0.059

*Note*: *Natural indirect effects (NIE) represent effects of loneliness on the preference for interpersonal distance within intimate space that are explained by the tested mediator*, *whereas natural direct effects (NDE) represent the effects of loneliness on the preference for interpersonal distance within intimate space that remain after controlling for the tested mediator*. *Omnibus statistics (Q) summarize mediational effects across levels of interpersonal distance preference and genders*.

### Discussion

Cross-validation of the model developed in Study 1 confirmed the association between loneliness and a preference for larger interpersonal distance within intimate space. Although measures of social closeness did significantly predict interpersonal distance preferences, no measure significantly mediated the association between loneliness and the preference for larger interpersonal distance within intimate space. Analyses showed that the natural direct effect of loneliness on interpersonal distance preference within intimate space (Table 5, right column) was only nominally affected by the inclusion of putative social closeness mediators. Although Study 2 was well-powered, the possibility that social closeness or self-other overlap may serve as a partial mediator with small effects may warrant future attention. Additionally, it is possible that other aspects of relationship quality not measured here might also influence the observed association between loneliness and preferences for larger interpersonal distance within intimate space. However, given the absence of significant mediation effects across a set of multifaceted measures of social closeness, follow-up research might more promisingly investigate other putative mechanisms as well, including hypervigilance for social threats.

## General discussion

The current research provides evidence for an association between loneliness and preferred interpersonal distance. Two competing hypotheses were considered. First, if loneliness primarily promotes approach and social reconnection, loneliness should be associated with a preference for smaller interpersonal distance [11,35]. Second, if loneliness simultaneously increases social approach to promote the repair or replacement of salutary relationships *and* increases the motivation to avoid or withdraw to promote self-preservation, then loneliness should be associated with a preference for larger interpersonal distance within intimate space, based on the known proximity gradients of approach-avoidance conflicts [13–15]. The results of Studies 1 and 2 were consistent with the second of these two hypotheses and *inconsistent* with the first. In Study 1, we found that lonelier participants preferred larger interpersonal distances within intimate space, and this effect was above and beyond the effects of gender, objective social isolation, anxiety, depressive symptomatology, and marital status. In Study 2, a direct replication of the model developed in Study 1 produced a consistent result, and mediation analyses failed to find significant evidence that measures of social closeness mediated the association between loneliness and interpersonal distance within intimate space. To better estimate the effect size for this association, we performed an internal meta-analysis synthesizing results from both studies. Results indicated that loneliness is associated with more than a doubling of the odds of a preference for larger interpersonal distance within intimate space (*OR* = 2.08, *95% CI* = [1.48, 2.93], *p* < .001; see Table G in S1 File).

Intimate space represents the inner circle of personal space and corresponds to what Horowitz et al. (1964) called the immediate body-buffer zone. Horowitz and colleagues (1964) found that this body-buffer zone was smaller with nonthreatening objects than with persons, and that compared to healthy controls, this immediate body-buffer zone was larger for schizophrenia patients, whom the authors characterized as “notorious for interpersonal withdrawal and avoidance” [12]. Notably, the association between loneliness and preference for larger interpersonal distance within intimate space–or what Horowitz et al. (1964) called the immediate body-buffer zone–may not be limited to humans. Aggressive behaviors among rhesus macaques include slapping, pushing, pulling fur, tail yanking, and biting [75]. When a macaque walks within arm’s reach of another monkey, the approaching monkey places itself within harm’s way. Research using our nonhuman primate model of loneliness showed that lonely, compared to non-lonely, rhesus monkeys were characterized by walkbys—approaching other adult monkeys, walking within arms distance, but then walking away before interacting with these monkeys [36]. The similarity of these observations in macaques to the association between loneliness and the expressed preferences for interpersonal distance in intimate space shown here should be interpreted cautiously, but additional comparative research could be informative.

Our results are also notable in light of recent neuroimaging studies investigating both loneliness and the regulation of interpersonal distance. Our recent resting-state fMRI study identified a putative neural mechanism for tonic hypervigilance for social threats in loneliness. Specifically, we found that lonelier participants exhibited elevated functional connectivity within the cingulo-opercular network, which is known from prior literature to be involved in the maintenance of tonic alertness [30]. Future studies may be merited to investigate whether functional connectivity within this brain network is predictive of the association between loneliness and the preference for larger interpersonal distance within intimate space. Additionally, Kennedy, Gläscher, Tyszka, and Adolphs (2009) found that personal space violations within proximal interpersonal distance, relative to distal interpersonal distance, preferentially activated the bilateral amygdala in healthy participants; in contrast, a patient with complete bilateral amygdalar damage demonstrated no discomfort at any proximal interpersonal distance from the experimenter or an unfamiliar confederate [76]. These findings indicate that the amygdala may mediate the discomfort associated with personal space violations, and they also provide further evidence for a neural dissociation between proximal and distal interpersonal distance zones, with threat responses increasing with physical nearness in healthy participants. An integration of these findings with the present results suggests the possibility that loneliness may be associated with increased bilateral activation of the amygdala in response to invasions of intimate space, an intriguing possibility which may be explored in future studies.

The current results were based on participants’ rated responses to vignettes, rather than behavioral responses to interpersonal space violations. Measures of interpersonal distance such as the Comfortable Interpersonal Distance scale [39], which is similar to the measure used in Studies 1 and 2, have previously demonstrated high convergent validity with stop-distance measures of physical interpersonal distance [77,78]. Nevertheless, the extension of the present work to behavioral (e.g., stop-distance) paradigms would provide further important evidence for the association between loneliness and interpersonal distance preferences identified here. For instance, based on the present results, we would predict that, compared to less lonely individuals, lonelier individuals would show a larger stop-distance for an intimate other relative to a confederate (stranger). Such a study could also potentially be conducted using an immersive virtual reality (IVR) paradigm. IVR is increasingly being used to study proxemics behavior [79–81], shows evidence of ecological validity [80,82–84], and enables researchers to precisely control both the social and physical environments experienced by participants [82,84].

It should also be noted that the present results are correlational, and further studies will be needed to establish the causal direction of the association between loneliness and interpersonal distance preference. The present results, in conjunction with robust evidence of hypervigilance for social threats in loneliness and the known proximity gradients of approach-avoidance conflicts, suggest that loneliness may lead to a preference for maintaining a larger distance from intimate others. However, it is also possible that distancing oneself from intimate others, whether emotionally or physically, could lead to increased loneliness. Tests of causation utilizing longitudinal cross-lagged panel analyses or strong experimental manipulations of loneliness are thus warranted to evaluate these alternatives. We hope that the present study lays the groundwork for such potential future investigations.

In sum, across human history, not only have humans survived and prospered by banding together to provide companionship, mutual protection and assistance, but a chief threat to a person’s reproductive success and survival has come from other humans. The same objective social relationship (e.g., a sibling, tribal member) can be perceived as caring and protective or as exploitive and threatening, based on a host of factors including an individual’s prior experiences with, attributed intentions toward, and inferred goals of others. According to Cacioppo and colleagues’ evolutionary model, the brain is the key organ for forming, monitoring, maintaining, repairing, and replacing salutary connections with others, and the perception of being socially isolated, even when around others, triggers the aversive state of loneliness, which is part of the biological warning machinery that alerts us to threats to our social body [18–20]. Among the posited behavioral consequences of loneliness is a heightened motivation to attend to and repair/replace perceived deficits in social relationships, and an implicitly heightened motivation for self-preservation (e.g., alertness for social threats, elevated activation of the hypothalamic pituitary-adrenocortical axis) to promote short-term survival. The current research provides evidence that loneliness is associated with a preference for larger interpersonal distance within intimate space (immediate body-buffer zone), a preference which may putatively function to confer added protection from social threats.

## Supporting information

S1 FileSupporting information for the association between loneliness and interpersonal distance.**Figure A.** Odds ratios and associated 95% Wald confidence intervals (reported as “OR (5th percentile– 95th percentile)” for each predictor in the Study 2 replication of the final model obtained from step-wise selection in Study 1. Negative odds ratios indicate preferences for smaller interpersonal distances, whereas positive odds ratios indicate preferences for larger interpersonal distances. Strangers served as the reference category for the social dimension variable. ^+^: *p* < .10, *: *p* < .05, **: *p* < .01, and ***: *p* < .001, respectively. **Figure B.** Probability of intimate interpersonal distance within each category (No Distance, 1–9 Inches, 10–25 Inches or Greater) as a function of standardized UCLA loneliness score (x-axis), with effects averaged across genders. Probabilities and confidence intervals represent predictions derived from a proportional odds mixed model utilizing meta-analytically combined coefficient estimates across studies 1 and 2. Probabilities on the dotted line at x = 0 represent the baseline probabilities for no effect of loneliness. **Figure C**. Odds ratios and associated 95% confidence intervals (reported as “OR (5th percentile– 95th percentile)” for each predictor in the final model obtained from step-wise selection with putative mediators in Study 2, extended to the group of individuals specified by participants as members of their intimate, relational, and collective social dimensions. “Closeness 1|2,” “Closeness 2|3,” and “Closeness 3|4” represent the nominal effects of closeness on each respective cumulative probability of the ordinal outcome (i.e., for $\log\left(\frac{P(Y\leq 1)}{P(Y>1)}\right),\log\left(\frac{P(Y\leq 2)}{P(Y>2)}\right)$, and $\log\left(\frac{P(Y\leq 3)}{P(Y>3)}\right)$, respectively), similarly to IOS. ^+:^ < .10, *: *p* < .05, **: *p* < .01, and ***: *p* < .001, respectively.(DOCX)

S1 Survey Study 1 survey. (PDF)

S2 Survey Study 2 survey. (PDF)
