# Supplementary material for: Loneliness predicts a preference for larger interpersonal distance within intimate space
Source: PLoS One. 2018 Sep 6;13(9):e0203491. doi: 10.1371/journal.pone.0203491 (PMC6126853; doi:10.1371/journal.pone.0203491)

**Section A. Replication of Results with International Participants Included in Study 1**

Due to known national differences in interpersonal distance regulation, we excluded non-US residents from our main analyses in Study 1. However, to investigate the robustness of our main results to this decision, we replicated our final model from stepwise selection using data from all 209 participants with complete data, including both US and non-US residents. Loneliness still predicted a preference for increased interpersonal distance within intimate space (*B* = .76, *Z* = 2.05, *p* = .040), and male gender predicted a preference for increased interpersonal distance across personal spaces (*B* = .91, *Z* = 2.21, *p* = .027).

**Section B. Treatment of Missing Data (Study 1)**

**Additional Details**

Interpersonal distance scores could be missing due to any of three possibilities: (1) participants selecting “I have no one in my _____ [Intimate, Relational, Collective, or Stranger] circle” (affected 28 interpersonal distance (IPD) observations distributed over 20 subjects), (2) participants selecting “Prefer not to answer,” (affected 6 IPD observations), or (3) non-responses (affected 2 IPD observations). A two-sample *t*-test revealed that participants who had missing IPD observations due to possibility (1) for at least one social dimension were significantly lonelier (scoring an average of 3.04 points higher on the UCLA loneliness scale) than those with no missing IPD data (*t*(165) = 2.85, *p* = .005). However, when objective isolation was included along with loneliness in a binomial logistic regression predicting missing IPD responses due to possibility (1), the effect of loneliness was no longer significant, as determined via a likelihood-ratio drop-test (*B* = .43, *χ^2^* (1) = 3.04, *p* = .08). Participants with missing IPD data due to possibility (2) or (3) did not differ from the rest of the sample in terms of loneliness (*p*’s > .45). Importantly, missing IPD responses alone did not result in the complete removal of any subjects (i.e., data for these subjects also had to be removed due to misssingness of other variables). Out of 20 subjects affected, 13 were only missing one IPD response, 6 were missing two, and 1 participant was missing three responses. Missing IPD data was distributed relatively evenly across social dimensions (Intimate: 8 affected observations; Relational: 2 affected observations; Collective: 8 affected observations; Stranger 10 affected observations).

The only other variables with missing data were depressive symptomatology (6 subjects), anxiety (7 subjects), and objective isolation (6 subjects), with no overlap between variables. A binomial logistic regression model was implemented to predict missing data for depressive symptomatology, but no predictor (social dimension, IPD , loneliness, isolation, anxiety, gender, and marriage status) was significantly associated with missingness (all *p*’s > .2). Implementing the same model to predict missing anxiety data, but replacing the anxiety predictor with depressive symptomatology, revealed that only male gender significantly predicted missing data (*B* = 1.00, *χ^2^* (1) = 6.45, *p* = .011). Missing responses for anxiety or depressive symptomatology questionnaires resulted solely from non-responses on items which were included at the end of the survey, suggesting the possibility that fatigue may have played a role in missing data for these variables.

Finally, we implemented the same model to predict missing isolation data, replacing the isolation predictor with anxiety. We noted that increased anxiety (*B* = -.18, *χ^2^* (1) = 3.76, *p* = .052) marginally predicted lower probability of missing data for isolation, whereas no other predictor was significant (all *p*’s > .1). To assure that covariate-dependent missingness for objective isolation did not significantly alter the obtained results, we re-estimated our final model after re-inclusion of data that was previously omitted due to missingness for this variable. This revealed a consistent effect of the loneliness by intimate dimension interaction (*B* = .90, *χ^2^* (1) = 2.03, *p* = .043).

**Section C. Ordinal vs. Continuous Measures of Interpersonal Distance Preference**

**Introduction**

In Study 1, we obtained our results using an ordinal measure of interpersonal distance preference, with distance categories selected based on known neurological subdivisions of perceptual and representational space (Ortigue, Megevand, Perren, Landis, & Blanke, 2006; Ortigue et al., 2001, 2003). Although interpersonal distance may be appropriately conceived of as an ordered series of proximal to distal zones/layers (Hall, 1966; S. Cacioppo, Grippo, London, Goossens, & Cacioppo, 2015 Ortigue et al., 2001, 2003, 2006), here we sought to investigate whether our main findings could also be obtained using an interval scale measurement of interpersonal distance preference. If reinforced, this insight could potentially aid future investigations by enabling simpler statistical analysis strategies, such as traditional mixed-effects models or ordinary least squares regression.

**Method**

To examine this possibility, we added a secondary continuous measure of interpersonal distance for each social dimension in Study 2, implemented using a sliding-bar type question in Qualtrics. This item was worded identically to, and presented in random order with respect to, our previously described ordinal measure. Responses were made on a sliding-scale from “Touching” (0) to “> 5 feet away (> 1.5 meters)” (20). We replicated the final model from Study 1 in the larger Study 2 sample using a linear mixed-effects model with maximum likelihood estimation with this interval scale data (Baayen, Davidson, & Bates, 2008). Mixed-modeling was implemented using the “lme4” package in R (Bates, Maechler, Bolker, & Walker, 2016). The significance of each predictor was determined using summary methods from the “lmerTest” package in R (Kuznetsova, Brockhoff, & Christensen, 2015), which implements Satterthwaite’s approximation to determine the effective degrees of freedom for *t*-tests.

Secondly, we sought to investigate whether our main results were robust to an inappropriate treatment of our ordinal interpersonal distance preference scale as an interval scale measure. We implemented a linear mixed-effects model replicating the final model from Study 1 separately using Study 1 and Study 2 data, in this case treating the ordinal response categories for interpersonal distance as integer values on an interval scale: 1 (“No distance”), 2 (“1-9 inches”), 3 (“10-25 inches”), and 4 (“2.5 feet or greater”).

**Result**

Utilizing a continuous measure of interpersonal distance preference, loneliness predicted preferences for increased interpersonal distance across social dimensions, a direction of effect consistent with our results using an ordinal measure (*B* = .45, *t*(1,349.2) = 1.86, *p* = .063). In this case, the effect of loneliness was not observed to vary significantly by social dimension (all *p*’s > .10). However, the pattern of simple effect sizes for loneliness within each social dimension was markedly congruent with that obtained from ordinal regression. Specifically, the largest effect size was evident for the intimate dimension, and a pattern of decreasing effect sizes was observed in each more distal social dimension (Intimate:Loneliness: *B* = .24; Relational:Loneliness: *B* = .15; Collective:Loneliness: *B* = -.18). We also noted that the effect of gender was non-significant in this model (*B* = .05, *t*(393.3) = .16, *p* > .8). After dropping the non-significant interaction between loneliness and social dimension, and the non-significant effect of gender, we noted that the main effect of loneliness was highly significant (*B* = .52, *t*(406.7) = 3.28, *p* = .001).

We also sought to gauge the robustness of our original ordinal measure of interpersonal distance preference to different analysis strategies. Treating this measure as though it were on an interval scale, and utilizing a linear mixed-effects model for analysis, we found that the effect of loneliness within the intimate dimension was preserved across both study 1 (*B* = .14, *t*(449.3) = 2.12, *p* = .034) and study 2 (*B* = .13, *t*(1157.6) = 2.84, *p* = .005).

**Discussion**

Results obtained using a continuous measure of interpersonal distance preference were largely consistent with those obtained using our original ordinal measure. Loneliness again predicted preferences for increased interpersonal distance, consistent with the hypervigilance for social threats described by the evolutionary model of loneliness, and *inconsistent* with the notion that loneliness acts merely as a signal motivating reconnection with others. Loneliness simple effects were again strongest within the intimate dimension and decreased in more distal dimensions, consistent with established proximity gradients of approach-avoidance conflicts (Åhs, Dunsmoor, Zielinski, & LaBar, 2015; Cacioppo & Bernston, 1994; Miller, 1959). However, the effect of loneliness did not vary significantly by social dimension in this model.

There are a number of considerations regarding the trade-offs between ordinal scales and continuous visual analog scales (VAS), such as the sliding-bar type question used here, which may help to explain this latter discrepancy. For instance, continuous VAS are known to sometimes suffer from lower reliability compared to equivalent ordinal scales (Couper, Tourangeau, Conrad, & Singer, 2006; Grigg, 1980; McKelvie, 1978). Although ratings of personal space preferences are known to vary markedly by social dimension, as was shown in the present study, some consistency might still be expected for within-subjects ratings across dimensions. Interestingly, Cronbach’s alpha was substantially higher in the present study for our ordinal measure of interpersonal distance preference across dimensions (α = 0.60) than for our continuous sliding-bar measure (α = 0.47). This difference was also evident if only the intimate, relational, and collective dimensions, which may be more conceptually similar than the stranger dimension, were considered (ordinal measure: α = 0.53; continuous measure: α = 0.41).

Secondly, VAS are most appropriate in cases in which participants are able to make fine distinctions between the levels of a construct (Couper et al., 2006). However, in cases in which a construct can be subdivided into a small set of meaningful divisions, as is the case for mental representations of interpersonal distance (Hall, 1966; S. Cacioppo et al., 2015; Ortigue et al., 2003, 2006), a scale limiting response options to these levels may produce more valid measurements than scales which seek to maximize discriminability and fine-grained differences between levels (Viswanathan, Sudman, & Johnson, 2004). These considerations, as well as the preliminary differences in sensitivity and reliability observed in the present study, suggest the possibility that ordinal measures of interpersonal distance preference, based upon meaningful divisions of mental representations of space, may constitute a more appropriate choice for gauging interpersonal distance preference in survey research.

**Section D. Stepwise Selection (Study 1)**

**Additional Details**

The proportional odds mixed model forward-selection steps proceeded as follows: **(Step 1)** A factor denoting personal space region yielded the largest increase in log-likelihood from the intercept-only model (*χ^2^* (3) = 427.40, *p* < .001); the proportional odds assumption was not rejected (*χ^2^* (3) = 4.42, *p* > .2). **(Step 2)** The proportional odds assumption was not rejected for any predictor (all *p*’s > .05), and loneliness was the only predictor which exhibited a significant interaction with personal space region (*χ^2^* (3) = 8.40, *p* = .038). Loneliness and a loneliness by personal space region interaction were added as ordinal effects (*χ^2^* (4) = 11.03, *p* = .026). **(Step 3)** Again, the proportional odds assumption was not rejected for any predictor (all *p*’s > .09), and an ordinal effect of gender was selected (*χ^2^* (1) = 6.18, *p* = .013). **(Step 4)** We again failed to reject the proportional odds assumption for any variable (all *p*’s > .09). The largest increase in log-likelihood for Step 4 resulted by adding an ordinal factor denoting marital status. However, this did not produce a significantly improved model compared to the previous one (*χ^2^* (1) = 2.42, *p* = .12). Therefore, this step yielded the final model: $CID=Personal Space Region*Loneliness+Gender$. See Table C for a full description of model selection steps and relevant statistics.

**Section E. Stepwise Selection of Putative Mediators (Study 2)**

**Additional Details**

Beginning from the Study 1 model, we used the same forward-selection procedure, this time sequentially adding putative mediators (social closeness, social closeness preference, IOS, frequency of contact, and frequency of contact preference). The model selection steps proceeded as follows: **(Step 1)** Adding social closeness (*χ2* (3) = 80.81, *p* < .001) as a nominal effect (proportional odds assumption rejected: *χ2* (2) = 27.23, *p* < .001) led to the largest increase in log-likelihood. **(Step 2)** IOS was added (*χ^2^* (3) = 11.97, *p* = .007) as a nominal effect (proportional odds assumption rejected: *χ^2^* (2) = 6.52, *p* = .038). **(Step 3)** The proportional odds assumption was not rejected for any remaining predictor (all *p*’s > .16). Social closeness preference interacted significantly with personal space region (*χ^2^* (2) = 6.67, *p* = .036), and this covariate and interaction were added (*χ^2^* (3) = 8.62, *p* = .035). **(Step 4)** Frequency of contact preference was added as an ordinal effect with no interactions (*χ^2^* (1) = 4.34, *p* = .037). **(Step 5)** An ordinal effect of frequency of contact produced the largest increase in log-likelihood for Step 5, but this did not significantly improve the model (*χ^2^* (1) = .03, *p* > .8). Therefore, the final model for Study 2 was as follows: $CID=Personal Space Region*Loneliness+Gender+{Social Closeness}_{Nominal}+{IOS}_{Nominal}+Personal Space Region*Social Closeness Preference+Frequency of Contact Preference$. Additional details for stepwise selection can be found in Table F.

**Section F. Testing for Mediation (Study 2)**

**Additional Details**

Using the method described by Imai & Yamamoto (2013), the *NDE* and *NIE* were calculated using the following formulas, wherein a primary mediator of interest (*M*) is allowed to be causally affected by a set of secondary mediators (*W*):

$${NDE}_{i}(t)={Pr(Y}_{i}(1,M_{i}\left( t,W_{i}\left( t \right) \right),W_{i}\left( 1 \right)))- {Pr(Y}_{i}(0,M_{i}\left( t,W_{i}\left( t \right) \right),W_{i}\left( 0 \right)))$$

${NIE}_{i}(t)={Pr(Y}_{i}(t,M_{i}\left( 1,W_{i}\left( 1 \right) \right),W_{i}\left( t \right)))- {Pr(Y}_{i}(t,M_{i}\left( 0,W_{i}\left( 0 \right) \right),W_{i}\left( t \right)))$,

for *t* = 0, 1, wherein *t* denotes a value set for the treatment variable (in this case, 0 (mean loneliness score) and 1 (+1 loneliness *SD*)), *Y_i_* denotes a given IPD level for observation *i*, *M_i_* denotes the value of a primary mediator as a function of the treatment variable, as well as potentially causally related alternative mediators *W*, which are also determined as a function of the treatment variable_._ The *NDE* and *NIE* are averaged over the values *t* of the treatment variable, and a bootstrapped distribution is generated for each effect. Thus, the *NIE* represents changes in the probability of an outcome (i.e., a given IPD level) that are transmitted through a primary mediator—which acts as a conduit for effects transmitted by the treatment variable or by the treatment acting through alternative mediators—while holding any direct effects of the treatment, or treatment effects mediated by alternative mediators, constant. In contrast, the *NDE* represents the remainder of the treatment effect which transmits either directly from the treatment to the outcome or via alternative mediators (Imai & Yamamoto, 2013).

In the present study, for each putative primary mediator variable (social closeness, IOS, frequency of contact, social closeness preference, frequency of contact preference), we created 5,000 bootstrapped samples by randomly selecting *m* participants’ data vectors (including all IPD observations) with replacement, with *m* equal to the number of participants included in the original sample (F. Harrell Jr & Harrell Jr, 2016¸ p. 252-257; Parke, Holford, & Charles, 1999, p. 23-25). Next, the following was completed for each bootstrapped sample: (1) a linear mixed-effects model was fit using the “lme4” package (Bates et al., 2016), in which the primary mediator served as the criterion variable and the treatment variable of interest (loneliness), other putative causally related mediators, and covariates (social dimension, gender) served as predictors; a random intercept for subject was also included; (2) an additional linear mixed-effects model was fitted for each putative secondary mediator, in which loneliness and covariates, but not the putative primary mediator, served as predictors; a random intercept for subject was also included (see Tingley, Yamamoto, Hirose, Keele, & Imai, 2014; Section 6, p. 25-27 for details); (3) a proportional odds mixed model was fit using the “ordinal” package (R. Christensen & Christensen, 2015), with IPD as the criterion variable, and with loneliness, the putative primary mediator, putative secondary mediators, and other covariates serving as predictors, including a random intercept for subject; (4) predicted values were obtained for each putative secondary mediator using the respective prediction equations from (2), given a standardized loneliness score of 0 (i.e., the mean score) and secondly, 1 (+1 *SD* of loneliness); (5) a predicted value for the primary putative mediator was obtained using the prediction equation from (1), given each standardized loneliness score and predicted values of putative secondary mediators from (4); (6) the expected probability of each possible ordinal outcome was then obtained using the ordinal model from (3) for each gender, and using specific predicted values of primary and secondary mediators variables obtained based on the formulas listed above for determining the *NDE* and *NIE*; (7) the appropriate expected probabilities of each ordinal outcome were subtracted to determine the *NDE* and *NIE* on a probability difference scale. The effects calculated across bootstrap samples yielded an approximation of the population distribution, and a 95% confidence interval was calculated for each effect based on this distribution.

To conduct significance testing, we created a bootstrapped null distribution of each effect. First, *NIE*’s and *NDE*’s were calculated as above, now using the original, non-bootstrapped, sample. These effects were then subtracted from the corresponding bootstrapped effects at each bootstrap iteration. To test for mediation effects across IPD levels and genders, we performed the omnibus test described by VanderWeele et al. (2016, eAppendix 3, p. 12-13) for each putative mediator. One-tailed *p*-values were used because *NIE* mediation effects were hypothesized to be of the same sign as the *NDE* of loneliness within intimate space, and the sign of the latter was known from the ordinal models implemented in studies 1 and 2.

**Section G. Internal Meta-analysis**

An internal meta-analysis, which synthesizes key results across multi-study research, often provides invaluable evidence for the robustness or fragility of the effects obtained (Kuminoff, Zhang, & Rudi, 2010). Here, we conducted an internal meta-analysis for the current research to investigate the robustness of our main finding of an association between loneliness and increased intimate IPD. Importantly, because the proportional odds assumption was met within each study, the log odds ratios can be validly meta-analytically combined across studies, despite the studies utilizing different cut-points for the ordinal outcome (Whitehead & Jones, 1994; A. Whitehead & Whitehead, 1991).

**Method**

We directly replicated the model obtained from stepwise selection in Study 1 here in our larger Study 2 sample. A meta-analytic-like estimate of effect size for the loneliness by intimate space interaction was calculated across Study 1 and Study 2 using the inverse variance method (Fleiss, 1993) implemented in the R package “meta” version 4.4-1 (G. Schwarzer & Schwarzer, 2016).

**Result**

A meta-analytic-like effect size was computed for the loneliness by intimate space interaction across Studies 1 and 2 (*OR* = 2.08, *95% CI* = [1.48, 2.93], *p* < .001), as well as for other predictors (see Table G for details). Figure B displays the probability of IPD for each ordinal level plotted as a function of standardized loneliness score. The predicted probabilities are based on the meta-analytically combined effects across studies 1 and 2.

**Discussion**

In summary, a direct replication of the Study 1 model and an internal meta-analysis across studies provided strong evidence that lonelier individuals prefer increased intimate IPD. The meta-analytic-like effect size computed across studies 1 and 2 underlined the robustness of this association: one standard deviation increase in loneliness was associated with 108% higher odds of larger IPD for intimate space.

**Section H. Extension of Findings to Individual-specific Measures**

**Introduction**

Study 1 results suggested that lonelier individuals prefer to maintain increased IPD within their intimate space, relative to average individuals who occupy this personal space region. Here, we investigated the robustness of this finding by determining whether the association also extends to specific individuals with whom participants prefer to interact in their intimate personal space.

**Method**

For each personal space, except for strangers, we asked participants to, “Please write the initials of a member of your ______ [Intimate, Relational, or Collective] circle who you: 1. Have the MOST FREQUENT CONTACT WITH (face-to-face, telephone, written, online or via a monitor screen): _____, 2. Have the LEAST FREQUENT CONTACT WITH (face-to-face, telephone, written, online or via a monitor screen): ______, 3. Feel the CLOSEST to (psychologically / emotionally): ______, and 4. Feel the LEAST CLOSE to (psychologically / emotionally): _______.” This prompt encouraged participants to select a group of individuals that spanned the range of a given personal space, in terms of both affective/cognitive closeness and frequency of contact. The goal was to minimize any differences in reporting that might arise if participants were simply asked to specify *any* member of their personal spaces. Notably, participants were able to specify the same individual for multiple categories. Further data was obtained with respect to each unique individual specified, including IPD, relationship type, social closeness, preference for closeness, IOS, frequency of contact, and preference for contact.

We then fit a proportional odds mixed model with participant coded as a random factor (i.e., participants gave repeated measures for each individual they specified). Social dimension was again included as a fixed factor, coded as having an interaction with loneliness. However, we now excluded the stranger dimension because we did not have individual-specific data for this dimension, therefore making the collective dimension the reference category. All the unique individuals specified by participants were included as single observations in this model (i.e., regardless of whether an individual was listed in multiple categories, the individual was only represented by a single observation). Odds ratios for the individual-specific effects of loneliness within social dimensions were compared to the previous average of social dimensions model. Additionally, *McFadden* *R^2^* and *adjusted McFadden* *R^2^* were calculated and compared qualitatively between individual-specific and average-based models.

**Result**

We confirmed the putative mediator model from stepwise selection in Study 2 in data for these specific individuals, with participant coded as a random factor. This model was highly significant compared to an intercept-only model (*χ*^2^ (15) = 1799.59, *p* < .001; *McFadden R*^2^ = .28; *adjusted McFadden R*^2^ = .27). *McFadden’s R*^2^ indicated an excellent fit, and *Count R*^2^ also indicated an excellent fit, with 1,294/2,619 = 49.41% of observations predicted correctly. In contrast, the modal guess (in this case, IPD level one, “no distance”) here resulted in 860/2,619 = 32.84% of observations predicted correctly. Adjusting for this modal guess yielded an adjusted *Count R*^2^ = .25, indicating a substantial improvement relative to the modal guess.

Again, the main effect of loneliness significantly predicted preferences for increased IPD (*B* = .38, *Z* = 2.61, *p* = .009, *OR* = 1.47, 95% *CI* = [1.10, 1.96]). The loneliness by intimate space simple effect again appeared to approach significance (*B* = .21, *Z* = 1.66, *p* = .096, *OR* = 1.23, *CI* = [.96, 1.58]), whereas the effect of loneliness within the relational space was non-significant (*B* = -.16, *Z* = -1.42, *p* > .15). Despite the marginal significance of the loneliness by intimate space interaction, a likelihood-ratio test again confirmed that the current model significantly improved upon a model which included only the loneliness main effect but no interaction (*χ*^2^ (2) = 9.67, *p* = .008). Additionally, the current model was significantly improved relative to a model which included neither the loneliness main effect nor interaction (*χ*^2^ (3) = 18.15, *p* < .001). Full regression results are shown in Table H, and a figure displaying odds ratios and confidence intervals can be found as Figure C.

**Discussion**

In sum, this analysis confirmed our findings from the Study 2 putative mediator model using individual-specific, as opposed to average, ratings of IPD for each personal space. This analysis enhanced the generalizability of our findings, demonstrating that the association between IPD and loneliness was not dependent upon contemplating mental representations of IPD for average members within each space, but also holds when contemplating IPD relative to a set of specific individuals. The main effect of loneliness here predicted 47% higher odds of larger IPD across personal space regions, whereas the loneliness by intimate space interaction marginally predicting 23% higher odds of IPD within larger IPD categories.

**Section I. Further Analysis for the Influence of the Romantic Partner Relationship Type**

Given that spousal dyads tend to require reduced intimate IPD (Hill, Blackham, & Crane, 1982) and that marriage is associated with reduced loneliness (e.g., Hawkley et al., 2008), we replicated the Study 1 model in our Study 2 individual-specific dataset, controlling for whether or not individuals specified by participants were their romantic partners.

**Results**

Knowing precisely which individuals were participants’ romantic partners allowed us to implement a stronger control of effects related to this relationship type. We replicated the Study 1 model, adding a factor indicating whether an individual was a participant’s romantic partner, as well as an interaction between this variable and personal space region. In this model, loneliness predicted significantly increased IPD in intimate space (*B* = .24, *χ^2^* (1) = 3.98, *p* = .046), and the main effect of loneliness also remained highly significant (*B* = .46, *χ^2^* (1) = 11.49, *p* < .001). On the other hand, the non-romantic partner by personal space region interaction was not significant (*χ^2^* (2) = 4.48, *p* = .106) and therefore was dropped. After dropping this interaction, the main effect of non-romantic partner strongly predicting increased IPD, relative to romantic partners (*B* = 3.41, *χ^2^* (1) = 145.19, *p* < .001; *OR* = 30.40, 95% *CI* = [22.90, 40.37]). Despite this, the association between loneliness increased IPD within intimate space remained significant when controlling for this factor (*B* = .25, *χ^2^* (1) = 4.14, *p* = .042; *OR* = 1.28, 95% *CI* = [1.13, 1.45]).

**Discussion**

In sum, we identified a strong effect of romantic partner, wherein non-romantic partners had 30.4 times higher odds of IPD within larger IPD categories compared to romantic partners. However, the loneliness by intimate space interaction remained significant when controlling for this factor, and an increase of one standard deviation of loneliness predicted 28% higher odds of IPD within larger IPD categories. This result strongly bolsters our interpretation that the association between loneliness and IPD within intimate space is not predicated on differences between lonely and non-lonely participants in terms of the romantic partner relationship type.

**References**

Baayen, R. H., Davidson, D. J., & Bates, D. M. (2008). Mixed-effects modeling with crossed random effects for subjects and items. *Journal of memory and language*, **59**, 390-412.

Barton, K. (2011). MuMIn: multi-model inference. R package, version 1.0. 0. R Foundation for Statistical Computing, Vienna, Austria.

Bates, D., Maechler, M., Bolker, B., Walker, S., Christensen, R. H. B., Singmann, H., ... & Bolker, M. B. (2016). Package ‘lme4’. In *R Package Version 1.1–10*.

Cacioppo, S., Grippo, A. J., London, S., Goossens, L., & Cacioppo, J. T. (2015). Loneliness clinical import and interventions. *Perspectives on Psychological Science, 10*(2), 238-249.

Christensen, R. H. B., & Christensen, M. R. H. B. (2015). Package ‘ordinal’. *Stand*, *19*, 2016.

Couper, M. P., Tourangeau, R., Conrad, F. G., & Singer, E. (2006). Evaluating the effectiveness of visual analog scales: A web experiment. *Social Science Computer Review*, *24*(2), 227-245.

Grigg, A. O. (1980). Some problems concerning the use of rating scales for visual assessment. *Journal of the Market Research Society*, *22*(1).

Hall, E. T. (1966). *The hidden dimension.* Garden City, NY: Doubleday.

Harrell Jr, F. E., & Harrell Jr, M. F. E. (2016). Package ‘Hmisc’. p. 252-257.

<ftp://ftp.uni-bayreuth.de/pub/math/statlib/s/Harrell/help/Hmisc/html/rm.boot.html>

Imai, K., & Yamamoto, T. (2013). Identification and sensitivity analysis for multiple causal mechanisms: revisiting evidence from framing experiments. *Political Analysis*, 141-171.

Kuminoff, N. V., Zhang, C., & Rudi, J. (2010). Are travelers willing to pay a premium to stay at a" green" hotel? Evidence from an internal meta-analysis of hedonic price premia. *Agricultural & Resource Economics Review*, **39**, 468.

Kuznetsova, A., Brockhoff, P. B., & Christensen, R. H. B. (2015). Package ‘lmerTest’. *R package version*, *2*(0).

McKelvie, S. J. (1978). Graphic rating scales—How many categories?. *British Journal of Psychology*, *69*(2), 185-202.

Nakagawa, S., & Schielzeth, H. (2013). A general and simple method for obtaining R2 from generalized linear mixed‐effects models. *Methods in Ecology and Evolution*, **4**, 133-142.

Ortigue, S., Megevand, P., Perren, F., Landis, T., & Blanke, O. (2006). Double dissociation between representational personal and extrapersonal neglect. *Neurology, 66*(9), 1414-1417.

Ortigue, S., Viaud‐Delmon, I., Annoni, J. M., Landis, T., Michel, C., Blanke, O., . . . Mayer, E. (2001). Pure representational neglect after right thalamic lesion. *Annals of neurology, 50*(3), 401-404.

Ortigue, S., Viaud-Delmon, I., Michel, C., Blanke, O., Annoni, J., Pegna, A., . . . Landis, T. (2003). Pure imagery hemi-neglect of far space. *Neurology, 60*(12), 2000-2002.

Parke, J., Holford, N. H., & Charles, B. G. (1999). A procedure for generating bootstrap samples

for the validation of nonlinear mixed-effects population models. *Computer methods and programs in biomedicine*, **59**, 19-29.

Tingley, D., Yamamoto, T., Hirose, K., Keele, L., & Imai, K. (2014). Mediation: R package for causal mediation analysis.

VanderWeele, T. J., Zhang, Y., & Lim, P. (2016). Brief Report: Mediation Analysis with an

Ordinal Outcome. *Epidemiology*, **27**, 651-655.

Viswanathan, M., Sudman, S., & Johnson, M. (2004). Maximum versus meaningful discrimination in scale response: Implications for validity of measurement of consumer perceptions about products. *Journal of Business Research*, *57*(2), 108-124.

Whitehead, A., & Jones, N. (1994). A meta-analysis of clinical trials involving different

classifications of response into ordered categories. *Statistics in medicine*, **13**, 2503-2515.

Whitehead, A., & Whitehead, J. (1991). A general parametric approach to the meta‐analysis of

randomized clinical trials. *Statistics in medicine*, **10**, 1665-1677.

**Supplementary Tables & Figures**

**Table A. Study 1 Sample Demographics**

| *Sample Demographics* | STUDY 1 |
| --- | --- |
| Demographic Variable | *n (%)* |
| *Age* |  |
| 18 to 24 | 27 (17.3) |
| 25 to 34 | 41 (26.3) |
| 35 to 44 | 13 (8.33) |
| 45 to 54 | 16 (10.3) |
| 55 to 64 | 32 (20.5) |
| 65 and Over | 27 (17.3) |
| *Gender* |  |
| Male | 55 (35.3) |
| Female | 101 (64.7) |
| *Ethnicity* |  |
| American Indian / Alaskan Native | 0 (00) |
| Asian / Pacific Islander | 5 (3.21) |
| Black / African American | 5 (3.21) |
| Hispanic American | 11 (7.05) |
| White / Caucasian | 121 (77.6) |
| Multiple Ethnicity / Other | 12 (7.69) |
| Socioeconomic Variable |  |
| *Household Income* |  |
| $0 to $9,999 | 10 (6.41) |
| $10,000 to $24,999 | 8 (5.13) |
| $25,000 to $49,999 | 23 (14.7) |
| $50,000 to $74,999 | 21 (13.5) |
| $75,000 to $99,999 | 13 (8.33) |
| $100,000 to $124,999 | 19 (12.2) |
| $125,000 to $149,999 | 9 (5.77) |
| $150,000 to $174,999 | 13 (8.33) |
| $175,000 to $199,999 | 5 (3.21) |
| $200,000 or More | 19 (12.2) |
| *Education Level* |  |
| Did Not Complete High School | 0 (00) |
| Graduated from High School | 3 (1.92) |
| Some College | 22 (14.1) |
| Graduated from College | 43 (27.6) |
| Some Graduate School | 22 (14.1) |
| Completed Graduate School | 65 (41.7) |
| Social Contact Variable |  |
| *Relationship Status* |  |
| Married | 82 (52.6) |
| Unmarried | 74 (47.4) |
| *Number of People* |  |
| In Household | 2.21 (1.12) |
| *Note. "Married" includes the categories married, civil union/domestic partnership, and single but cohabiting. "Unmarried" includes "Single, never married," divorced, widowed, and separated.* | |

| **Table B. Study 1 Social Dimension Composition** | |
| --- | --- |
| *Social Circle Composition* | Study 1 |
| Relationship Type (Intimate Circle) | *n (%)* |
| Best friend(s) | 96 (61.5) |
| Other | 96 (61.5) |
| Romantic Partner | 93 (59.6) |
| Mother | 54 (34.6) |
| Sister(s) | 49 (31.4) |
| Father | 40 (25.6) |
| Brother(s) | 37 (23.7) |
| Grandparent(s) | 7 (4.49) |
| Relationship Type (Relational Circle) | *n (%)* |
| Best friend(s) | 112 (71.8) |
| Other | 82 (52.6) |
| Brother(s) | 46 (29.5) |
| Mother | 39 (25) |
| Father | 39 (25) |
| Sister(s) | 38 (24.4) |
| Grandparent(s) | 12 (7.69) |
| Romantic Partner | 10 (6.41) |
| Collective Type (Collective Circle) | *n (%)* |
| Professional Association(s) | 90 (57.7) |
| Other | 62 (39.7) |
| Club(s) | 60 (38.5) |
| Committee(s) | 46 (29.5) |
| Religious Group | 40 (25.6) |
| Sport Team(s) | 39 (25) |
| Nation(s) | 14 (8.97) |
| *Note. n refers to the number of participants who specified a given category, and % refers to the percentage of all participants who specified this category.* | |

Notably, the number of members selected for each personal space was consistent with the findings of Hawkley et al. (2005): intimate (M = 4.49 individuals, SD = 3.38), relational (M = 12.15 individuals, SD = 10.55), collective (M = 12.44 collectives/groups, SD = 63.35).

**Table C. Step-wise Model Selection for Study 1**

|  | **Step 1: Intercept Only** | |  | **Step 2: Add 1^st^ Covariate** | |
| --- | --- | --- | --- | --- | --- |
| Covariate | *Coef (SE)* | *AIC, Log-Likelihood* |  | *Coef (SE)* | *AIC, Log-Likelihood* |
| Social Dimension |  |  |  |  |  |
| Intimate |  |  |  | -8.06 (.66) *** | 848.25, -418.1255 *** |
| Relational |  |  |  | -4.66 (.45) *** | 848.25, -418.1255 *** |
| Collective |  |  |  | -2.19 (.36) *** | 848.25, -418.1255 *** |
| Psychosocial |  |  |  |  |  |
| Loneliness |  |  |  | .16 (.09) + | 1268.17, -630.08 + |
| Anxiety |  |  |  | .09 (.08) | 1270.56, -631.28 |
| Depressive Sympt. |  |  |  | .03 (.09) | 1271.50, -631.75 |
| Demographic |  |  |  |  |  |
| Gender: Male |  |  |  | .46 (.17) ** | 1264.60, -628.30 ** |
| Social Network |  |  |  |  |  |
| Married |  |  |  | -.18 (.20) | 1270.56, -631.28 |
| Objective Isolation |  |  |  | -.02 (.08) | 1271.62, -631.81 |
| Thresholds |  |  |  |  |  |
| 1\|2 | -.60 (.10) | 1269.65, -631.83 |  | In Model | In Model |
| 2\|3 | .37 (.09) | 1269.65, -631.83 |  | In Model | In Model |
|  |  |  |  |  |  |
|  | *Variance* |  |  | *Variance* |  |
| Random Intercept (Subj.) | 0.18 | 1269.65, -631.83 |  | In Model | In Model |
|  |  |  |  |  |  |
|  | **Step 3: Add 2^nd^ Covariate** | |  | **Step 4: Add 3^rd^ Covariate** | |
| Covariate | *Coef (SE)* | *AIC, Log-Likelihood* |  | *Coef (SE)* | *AIC, Log-Likelihood* |
| Social Dimension |  |  |  |  |  |
| Intimate | In Model | In Model |  | In Model | In Model |
| Relational | In Model | In Model |  | In Model | In Model |
| Collective | In Model | In Model |  | In Model | In Model |
| Psychosocial |  |  |  |  |  |
| Loneliness | .14 (.38) | 845.22, -412.61 * |  | In Model | In Model |
| Loneliness*Int. | .94 (.46) * | 845.22, -412.61 * |  | In Model | In Model |
| Loneliness*Rel. | .23 (.38) | 845.22, -412.61 * |  | In Model | In Model |
| Loneliness*Col. | -.12 (.37) | 845.22, -412.61 * |  | In Model | In Model |
| Anxiety | .22 (.22) | 849.26, -417.63 |  | .04 (.26) | 847.19, -412.60 |
| Depressive Sympt. | .09 (.24) | 850.10, -418.05 |  | -.21 (.30) | 846.70, -412.35 |
| Demographic |  |  |  |  |  |
| Gender: Male | 1.24 (.47) ** | 843.06, -414.53 ** |  | 1.18 (.48) * | 841.04, -409.52 * |
| Social Network |  |  |  |  |  |
| Married | -.59 (.45) | 848.50, -417.25 |  | -.78 (.46) + | 844.32, -411.16 + |
| Objective Isolation | -.11 (.22) | 850.02, -418.01 |  | -.30 (.24) | 845.73, -411.86 |
| Thresholds |  |  |  |  |  |
| 1\|2 | In Model | In Model |  | In Model | In Model |
| 2\|3 | In Model | In Model |  | In Model | In Model |
|  |  |  |  |  |  |
|  | *Variance* |  |  | *Variance* |  |
| Random Intercept (Subj.) | In Model | In Model |  | In Model | In Model |

**Table D. Study 2 Sample Demographics**

| *Sample Demographics* | STUDY 2 |
| --- | --- |
| Demographic Variable | *n (%)* |
| *Age* |  |
| 18 to 24 | 46 (11.4) |
| 25 to 34 | 185 (45.7) |
| 35 to 44 | 108 (26.7) |
| 45 to 54 | 41 (10.1) |
| 55 to 64 | 18 (4.4) |
| 65 and Over | 7 (1.7) |
| *Gender* |  |
| Male | 197 (48.6) |
| Female | 208 (51.4) |
| *Ethnicity* |  |
| American Indian / Alaskan Native | 2 (0.5) |
| Asian / Pacific Islander | 31 (7.7) |
| Black / African American | 20 (4.9) |
| Hispanic American | 18 (4.4) |
| White / Caucasian | 334 (82.5) |
| Multiple Ethnicity / Other | 0 (0) |
| Socioeconomic Variable | *n (%)* |
| *Household Income* |  |
| $0 to $9,999 | 13 (3.2) |
| $10,000 to $24,999 | 78 (19.3) |
| $25,000 to $49,999 | 134 (33.1) |
| $50,000 to $74,999 | 78 (19.3) |
| $75,000 to $99,999 | 36 (8.9) |
| $100,000 to $124,999 | 29 (7.2) |
| $125,000 to $149,999 | 12 (3) |
| $150,000 to $174,999 | 3 (0.7) |
| $175,000 to $199,999 | 2 (0.5) |
| $200,000 or More | 5 (1.2) |
| *Education Level* |  |
| Did Not Complete High School | 3 (0.7) |
| Graduated from High School | 52 (12.8) |
| Some College | 149 (36.8) |
| Graduated from College | 154 (38) |
| Some Graduate School | 20 (4.9) |
| Completed Graduate School | 27 (6.7) |
| Social Contact Variable | *n (%)* |
| *Relationship Status* |  |
| Married | 210 (51.9) |
| Unmarried | 195 (48.2) |
| *Number of People* | *Mean (SD)* |
| In Household | 2.6 (1.4) |
| *Note. "Married" includes the categories married, civil union/domestic partnership, and single but cohabiting. "Unmarried" includes "Single, never married," divorced, widowed, and separated.* | |

| **Table E. Study 2 Social Dimension Composition** | |
| --- | --- |
| ***Social Circle Composition*** | **Study 2** |
| Relationship Type (Intimate Circle) | *n (%)* |
| Romantic Partner | 254 (62.7) |
| Parent(s) | 216 (53.3) |
| Close Friend(s) | 154 (38) |
| Sibling(s) | 146 (36.05) |
| Children | 106 (26.2) |
| Other Relative(s) | 28 (6.9) |
| Friend(s) | 25 (6.2) |
| Grand Parent(s) | 22 (5.4) |
| Relationship Type (Relational Circle) | *n (%)* |
| Close Friend(s) | 212 (52.4) |
| Friend(s) | 193 (47.7) |
| Sibling(s) | 129 (31.9) |
| Other Relative(s) | 128 (31.6) |
| Parent(s) | 121 (29.9) |
| Colleague(s) | 59 (14.6) |
| Grand Parent(s) | 43 (10.6) |
| Children | 26 (6.4) |
| Collective Type (Collective Circle) | *n (%)* |
| Colleague(s) | 112 (27.7) |
| Friend(s) | 86 (21.2) |
| Acquaintance(s) | 81 (20) |
| Leisure Activity Partner(s) | 81 (20) |
| Teammate(s) | 80 (19.8) |
| Club Member(s) | 78 (19.3) |
| Religious Organization Member(s) | 66 (16.3) |
| Other | 62 (15.3) |
| Professional Association Member(s) | 49 (12.1) |
| Classmate(s) | 36 (8.9) |
| Committee Member(s) | 18 (4.4) |
| *Note. n refers to the number of participants who specified a given category, and % refers to the percentage of all participants who specified this category.* | |

**Table F. Step-wise Model Selection for Study 2**

| **Step 1: Add 1^st^ Putative Mediator** | | |  | **Step 2: Add 2^nd^ Putative Mediator** | | | |
| --- | --- | --- | --- | --- | --- | --- | --- |
| Covariate | *Coef (SE)* | *AIC, Log-Likelihood* |  | Covariate | *Coef (SE)* | | *AIC, Log-Likelihood* |
| Social Dimension |  |  |  | Social Dimension |  | |  |
| Intimate | In Model | In Model |  | Intimate | In Model | | In Model |
| Relational | In Model | In Model |  | Relational | In Model | | In Model |
| Psychosocial |  |  |  | Psychosocial |  | |  |
| Loneliness | In Model | In Model |  | Loneliness | In Model | | In Model |
| Loneliness*Int. | In Model | In Model |  | Loneliness*Int. | In Model | | In Model |
| Loneliness*Rel. | In Model | In Model |  | Loneliness*Rel. | In Model | | In Model |
| Social Closeness |  |  |  | Social Closeness |  | |  |
| 1\|2 | .53 (.07) *** | 2286.44, -1130.22 |  | 1\|2 | In Model | | In Model |
| 2\|3 | .30 (.05) *** | 2286.44, -1130.22 |  | 2\|3 | In Model | | In Model |
| 3\|4 | .14 (.06) ** | 2286.44, -1130.22 |  | 3\|4 | In Model | | In Model |
| IOS |  |  |  | IOS |  | |  |
| 1\|2 | .68 (.10) *** | 2305.45, -1139.72 |  | 1\|2 | .33 (.12) ** | | 2280.47, -1124.23 |
| 2\|3 | .46 (.09) *** | 2305.45, -1139.72 |  | 2\|3 | .28 (.12) * | | 2280.47, -1124.23 |
| 3\|4 | .12 (.10) | 2305.45, -1139.72 |  | 3\|4 | -.06 (.14) | | 2280.47, -1124.23 |
| Closeness Pref. | -.29 (.14) * | 2359.11, -1168.55 |  | Closeness Pref. | .34 (.26) | | 2282.94, -1125.47 |
| Freq. Contact | -.27 (.09) ** | 2353.07, -1165.54 |  | Closeness Pref.*Int. | -.75 (.38) * | | 2282.94, -1125.47 |
| Freq. Contact Pref. | -.39 (.12) ** | 2352.82, -1165.41 |  | Closeness Pref.*Rel. | -.81 (.31) ** | | 2282.94, -1125.47 |
| Demographic |  |  |  | Freq. Contact | .01 (.10) | | 2288.42, -1130.21 |
| Gender: Male | In Model | In Model |  | Freq. Contact Pref. | -.33 (.13) ** | | 2281.70, -1126.85 |
| Thresholds |  |  |  | Demographic |  | |  |
| 1\|2 | In Model | In Model |  | Gender: Male | In Model | | In Model |
| 2\|3 | In Model | In Model |  | Thresholds |  | |  |
| 3\|4 | In Model | In Model |  | 1\|2 | In Model | | In Model |
|  | *Variance* |  |  | 2\|3 | In Model | | In Model |
| Random Intercept (Subj.) | In Model | In Model |  | 3\|4 | In Model | | In Model |
|  |  |  |  |  | *Variance* | |  |
| **Step 3: Add 3^rd^ Putative Mediator** | | |  | Random Intercept (Subj.) | In Model | | In Model |
| Covariate | *Coef (SE)* | *AIC, Log-Likelihood* |  |  |  | |  |
| Social Dimension |  |  |  | **Step 4: Add 4^th^ Putative Mediator, Finish** | | | |
| Intimate | In Model | In Model |  | Covariate | | *Coef (SE)* | *AIC, Log-Likelihood* |
| Relational | In Model | In Model |  | Social Dimension | |  |  |
| Psychosocial |  |  |  | Intimate | | In Model | In Model |
| Loneliness | In Model | In Model |  | Relational | | In Model | In Model |
| Loneliness*Int. | In Model | In Model |  | Psychosocial | |  |  |
| Loneliness*Rel. | In Model | In Model |  | Loneliness | | In Model | In Model |
| Social Closeness |  |  |  | Loneliness*Int. | | In Model | In Model |
| 1\|2 | In Model | In Model |  | Loneliness*Rel. | | In Model | In Model |
| 2\|3 | In Model | In Model |  | Social Closeness | |  |  |
| 3\|4 | In Model | In Model |  | 1\|2 | | In Model | In Model |
| IOS |  |  |  | 2\|3 | | In Model | In Model |
| 1\|2 | In Model | In Model |  | 3\|4 | | In Model | In Model |
| 2\|3 | In Model | In Model |  | IOS | |  |  |
| 3\|4 | In Model | In Model |  | 1\|2 | | In Model | In Model |
| Closeness Pref. | .33 (.26) | 2277.85, -1119.92 |  | 2\|3 | | In Model | In Model |
| Closeness Pref.*Int. | -.73 (.38) | 2277.85, -1119.92 |  | 3\|4 | | In Model | In Model |
| Closeness Pref.*Rel. | -.77 (.31) * | 2277.85, -1119.92 |  | Closeness Pref. | | In Model | In Model |
| Freq. Contact | .03 (.10) | 2282.39, -1124.19 |  | Closeness Pref.*Int. | | In Model | In Model |
| Freq. Contact Pref. | -.34 (.13) ** | 2275.50, -1120.75 |  | Closeness Pref.*Rel. | | In Model | In Model |
| Demographic |  |  |  | Freq. Contact | | -.02 (.10) | 2279.82, -1119.91 |
| Gender: Male | In Model | In Model |  | Freq. Contact Pref. | | -.33 (.16) * | 2275.51, -1117.76 |
| Thresholds |  |  |  | Demographic | |  |  |
| 1\|2 | In Model | In Model |  | Gender: Male | | In Model | In Model |
| 2\|3 | In Model | In Model |  | Thresholds | |  |  |
| 3\|4 | In Model | In Model |  | 1\|2 | | In Model | In Model |
|  | *Variance* |  |  | 2\|3 | | In Model | In Model |
| Random Intercept (Subj.) | In Model | In Model |  | 3\|4 | | In Model | In Model |
|  |  |  |  |  | | *Variance* |  |
|  |  |  |  | Random Intercept (Subj.) | | In Model | In Model |

| **Table G. Meta-analytically Combined Effects** | |
| --- | --- |
| Covariate | *OR (95% CI)* |
| Social Dimension |  |
| Intimate | <.001 (<.001 - .001) |
| Relational | .012 (.008 - .018) |
| Collective | .107 (.078 - .146) |
| Psychosocial |  |
| Loneliness | 1.27 (.948 - 1.70) |
| Loneliness*Intimate | 2.08 (1.48 - 2.93) |
| Loneliness*Relational | 1.14 (.848 - 1.53) |
| Loneliness*Collective | 1.12 (.826 - 1.51) |
| Demographic |  |
| Gender: Male | 2.21 (1.48 - 3.30) |
| Thresholds |  |
| 1\|2 | .004 (.002 - .006) |
| 2\|3 | .058 (.040 - .085) |
| 3\|4 | - |
| Random Intercept (Subj.) | - |
| *Note. Combined effect sizes across studies 1 and 2 were obtained using the "meta" package for R. Note. The 95% confidence interval for the standard deviation of the random intercept was determined using the profile likelihood method.* | |

| **Table H. Study 2 Final Model Confirmed in Individual Subjects** | | | |
| --- | --- | --- | --- |
| **Covariate** | ***Coef (SE)*** | ***Z (p)*** | ***OR (95% CI)*** |
| Social Dimension |  |  |  |
| Intimate | -2.57 (.18) | -14.08 (<.001) | .08 (.05 - .11) |
| Relational | -.94 (.13) | -7.08 (<.001) | .39 (.30 - .51) |
| Demographic |  |  |  |
| Gender: Male | .58 (.26) | 2.24 (.025) | 1.79 (1.08 - 2.97) |
| Psychosocial |  |  |  |
| Loneliness | .38 (.15) | 2.61 (.009) | 1.47 (1.10 - 1.96) |
| Loneliness*Int. | .21 (.13) | 1.66 (.096) | 1.23 (.96 - 1.58) |
| Loneliness*Rel. | -.16 (.11) | -1.42 (.157) | .85 (.69 - 1.06) |
| Putative Mediators |  |  |  |
| Freq. Contact Pref. | -.14 (.10) | -1.39 (.164) | .87 (.71 - 1.06) |
| Closeness Pref. | -.42 (.16) | -2.65 (.008) | .66 (.48 - .90) |
| Closeness Pref.*Int. | -.09 (.19) | 0.46 (.649) | .92 (.63 - 1.33) |
| Closeness Pref.*Rel. | -.21 (.17) | -1.21 (.227) | .81 (.58 - 1.14) |
| Social Closeness |  |  |  |
| 1\|2 | -.36 (.04) | -8.54 (<.001) | .70 (.64 - .76) |
| 2\|3 | -.32 (.04) | -8.37 (<.001) | .73 (.68 - .78) |
| 3\|4 | -.35 (.05) | -7.08 (<.001) | .70 (.64 - .78) |
| IOS |  |  |  |
| 1\|2 | -.44 (.07) | -6.20 (<.001) | .65 (.56 - .74) |
| 2\|3 | -.27 (.07) | -3.67 (<.001) | .77 (.66 - .88) |
| 3\|4 | -.03 (.10) | -.35 (.730) | .97 (.80 - 1.17) |
| Thresholds |  |  |  |
| 1\|2 | -6.84 (.33) | -20.64 (<.001) | .001 (<.001 - .002) |
| 2\|3 | -2.71 (.25) | -10.66 (<.001) | .07 (.04 - .11) |
| 3\|4 | .77 (.27) | 2.87 (.004) | 2.17 (1.28 - 3.68) |
|  | ***SD*** | ***SD 95% CI*** |  |
| Random Intercept (Subj.) | 2.39 | (2.09, 2.73) | - |
| Model Fit |  | *Note. The 95% confidence interval for the standard deviation of the random intercept was determined using the profile likelihood method.* | |
| AIC | 4783.01 |  |  |
| Log-Likelihood | -2371.50 |  |  |
| Adj. McFadden R^2^ | 0.27 |  |  |
| Count R^2^ | 0.49 |  |  |
| Adjusted Count R^2^ | 0.25 |  |  |

**
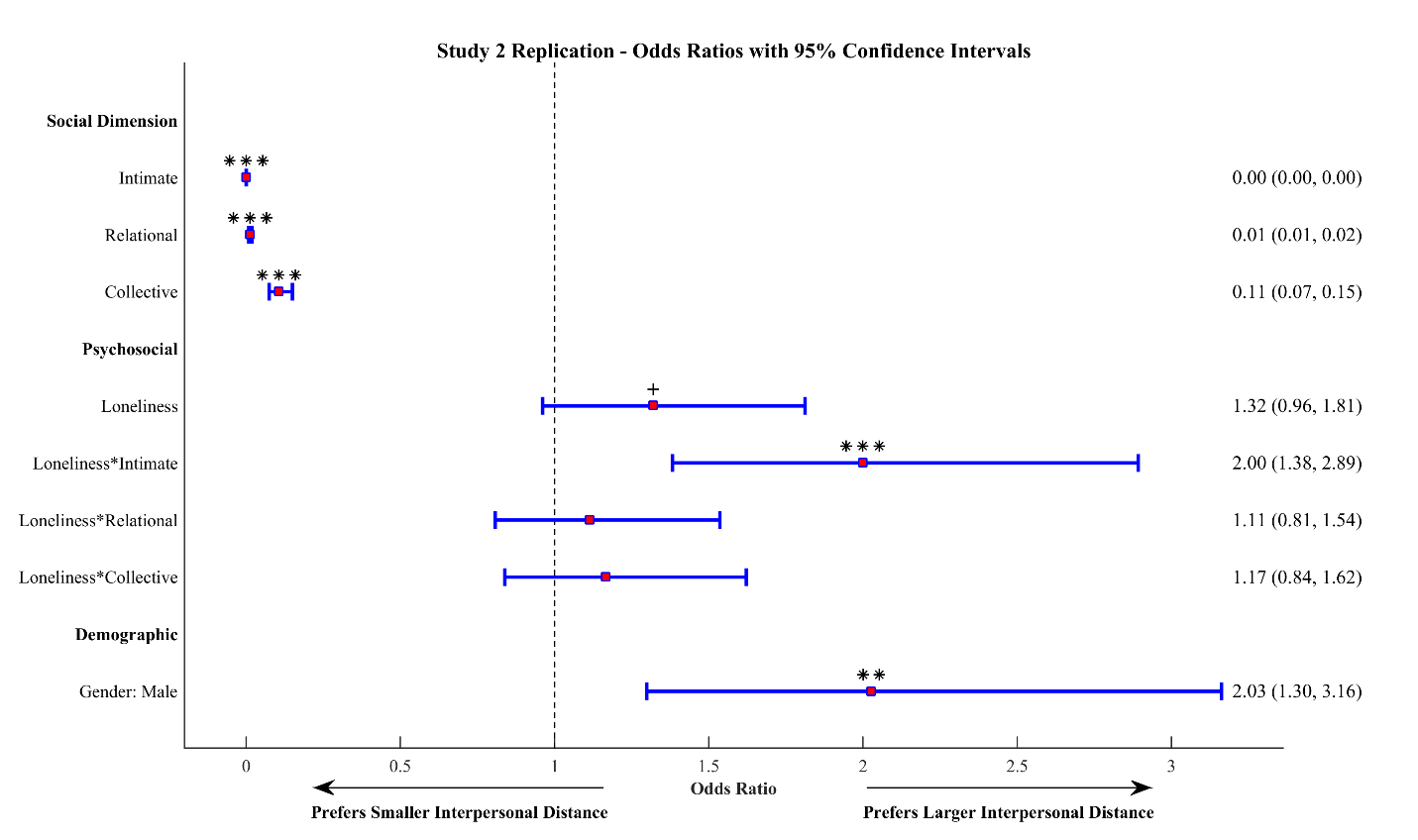
**


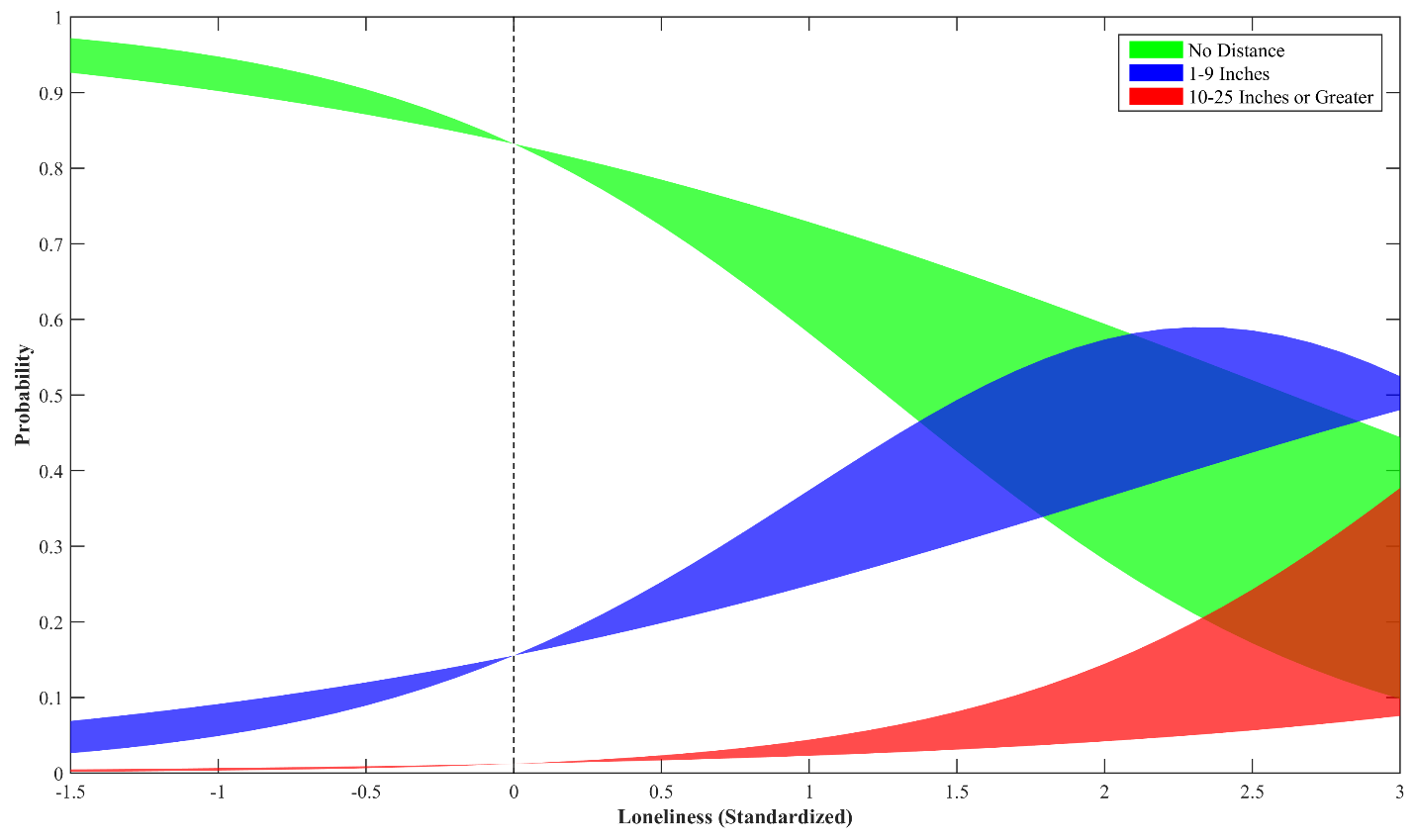


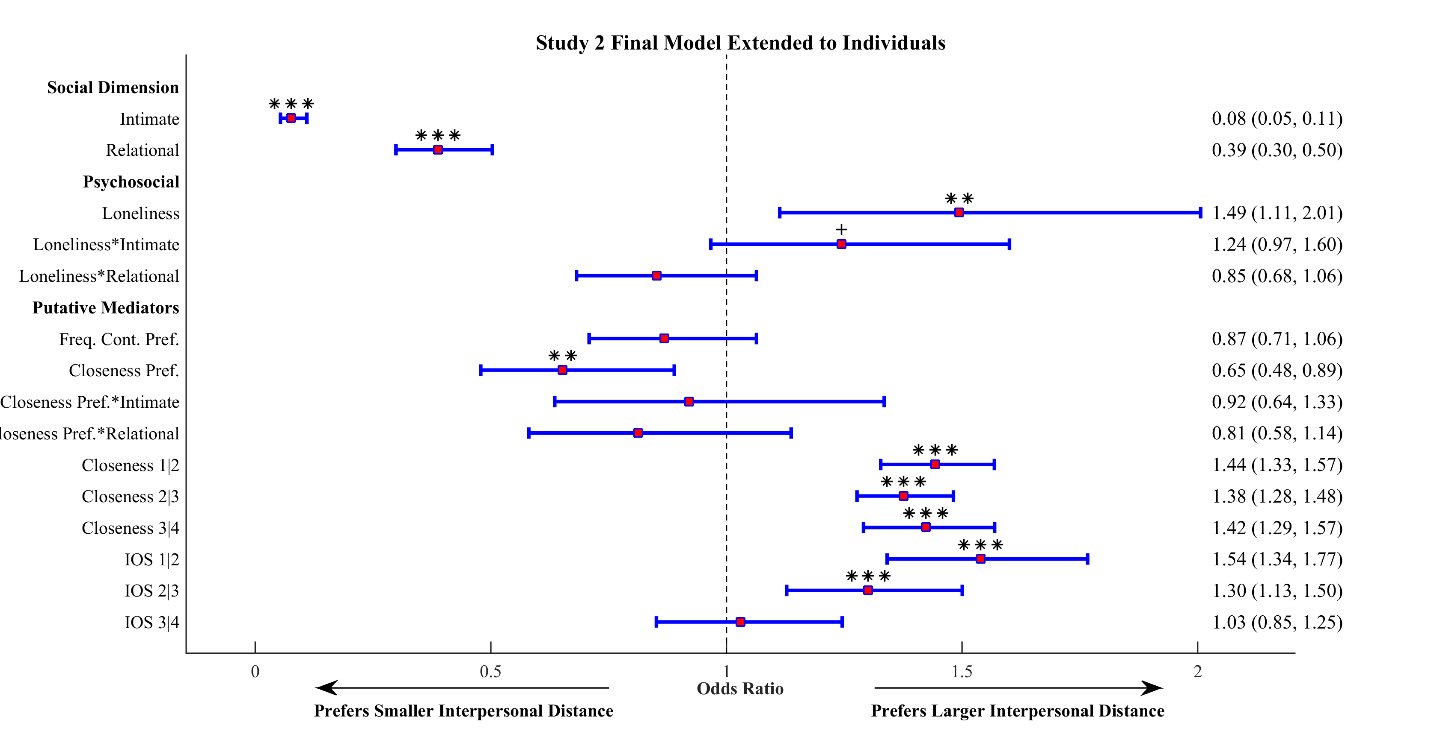

Supplement: S1 File — Figure A. Odds ratios and associated 95% Wald confidence intervals (reported as “OR (5th percentile– 95th percentile)” for each predictor in the Study 2 replication of the final model obtained from step-wise selection in Study 1. Negative odds ratios indicate preferences for smaller interpersonal distances, whereas positive odds ratios indicate preferences for larger interpersonal distances. Strangers served as the reference category for the social dimension variable. +: p < .10, *: p < .05, **: p < .01, and ***: p < .001, respectively. Figure B. Probability of intimate interpersonal distance within each category (No Distance, 1–9 Inches, 10–25 Inches or Greater) as a function of standardized UCLA loneliness score (x-axis), with effects averaged across genders. Probabilities and confidence intervals represent predictions derived from a proportional odds mixed model utilizing meta-analytically combined coefficient estimates across studies 1 and 2. Probabilities on the dotted line at x = 0 represent the baseline probabilities for no effect of loneliness. Figure C. Odds ratios and associated 95% confidence intervals (reported as “OR (5th percentile– 95th percentile)” for each predictor in the final model obtained from step-wise selection with putative mediators in Study 2, extended to the group of individuals specified by participants as members of their intimate, relational, and collective social dimensions. “Closeness 1|2,” “Closeness 2|3,” and “Closeness 3|4” represent the nominal effects of closeness on each respective cumulative probability of the ordinal outcome (i.e., for logP(Y≤1)P(Y>1),logP(Y≤2)P(Y>2), and logP(Y≤3)P(Y>3), respectively), similarly to IOS. +: < .10, *: p < .05, **: p < .01, and ***: p < .001, respectively. (DOCX) [file pone.0203491.s001.docx]
