# Supplementary material for: Loneliness predicts a preference for larger interpersonal distance within intimate space
Source: PLoS One. 2018 Sep 6;13(9):e0203491. doi: 10.1371/journal.pone.0203491 (PMC6126853; doi:10.1371/journal.pone.0203491)
Supplement: S1 Survey — (PDF) [file pone.0203491.s002.pdf]

THE UNIVERSITY OF CHICAGO

Social Distance Project

Consent form

Hello:

You are being asked to participate in a research study on social connections that has been approved by the University of Chicago Institutional Review Board.

This page describes the study procedures, the risks and benefits of participation, as well as how your confidentiality will be maintained.

Please take your time to read this document and feel comfortable making a decision whether to participate or not. This process is called informed consent.

WHY IS THIS STUDY BEING DONE? The purpose of this research study is to better understand social connections worldwide through a series of 70 questions. Through these questions, the investigators hope that it will be possible for them to better characterize the current landscape of social connections.

HOW MANY PEOPLE WILL TAKE PART IN THE STUDY? In order to best grasp the current social landscape, we are hoping to test as many participants as possible.

WHAT IS INVOLVED IN THE STUDY? During this study, you will be asked to fill in 70 questions. Filling out these 70 questions will take approximately 15 minutes.

WHAT ARE THE RISKS OF THE STUDY?
Likely Risks: • General stiffness and back discomfort from remaining in one position • Some people become anxious while answering questions. This anxiety is more likely for those who tend to feel uncomfortable or fearful when answering demographic questions. If you feel uncomfortable in answering some questions, you can skip the question or select the answer "Prefer not to answer" when applicable.

Less Likely Risks: • There is a possible risk of confidentiality loss associated with this study. Although we are not collecting your name or any other identifiers, your IP (Internet Protocol) address is automatically stored in online surveys. We will help protect your confidentiality by keeping all data in locked filing cabinets and password-protected computers. These data will only be available to the research team.

WHAT ABOUT CONFIDENTIALITY? This survey is anonymous. No identifiers will be collected. If you decide to participate, you will be assigned a unique participant number. The analysis of your responses will be performed with this code number in the data field where a name would ordinarily go. Neither your name nor any other personal information will be recorded. After the survey, all responses will be kept in a password protected computer, and will only be accessible to investigators. Data from this study may be used in research publications or presentations.

ARE THERE ANY BENEFITS TO TAKING PART IN THE STUDY? Your participation in this study will have NO direct benefit to you personally.

WHAT OTHER OPTIONS ARE THERE? You may choose not to participate. The decision whether or not you wish to participate in this study will not affect your participation in future psychology studies or future care at the University of Chicago medical facilities.

WHAT ARE THE COSTS? There are no monetary costs to you for participating in this study.

WILL I BE PAID FOR MY PARTICIPATION? You will NOT be paid for your participation. Your participation is VOLUNTARY. You may withdraw participation at any time.

WHAT ARE MY RIGHTS AS A PARTICIPANT? Taking part in this study is voluntary. You may choose not to participate at any time during the study. If you choose to no longer be in the study, your participation will be immediately disregarded. If you start to take this survey and then change your mind, you can close the browser -- we will not use any data about you if you withdraw from the survey.

WHO DO I CALL IF I HAVE QUESTIONS OR PROBLEMS?

If you have further questions about the study, you may call the principal investigator of this study, Dr. Stephanie Cacioppo, at 773-702-1962.

If you have any questions concerning your rights as a participant in this research study or if you have any concerns about this research study, you may contact the University of Chicago's Social and Behavioral Institutional Review Board (IRB), 5835 S. Kimbark Ave. Chicago, IL 60637, which is concerned with the protection of human subjects in research projects. You may reach the Committee office between 8:00 am and 5:00 pm, Monday through Friday, by calling (773) 702-5064 or emailing : sbs-irb@uchicago.edu or writing to the University of Chicago's Social and Behavioral Institutional Review Board (IRB), 5835 S. Kimbark Ave. Chicago, IL 60637, USA.

1. CONSENT FORM
I understand the research project and the procedures associated with it. The experimental procedures have been identified and no guarantee has been given about the possible results. I am at least 18 years old and I agree to participate in this study. This consent form document does not have an expiration date. My participation is voluntary. I can answer "No" in this form if I do not want to be part of this research study.

- ○ Yes
- ○ No

2. Which of the following best describes your current relationship status?

- Married
- Widowed
- Divorced
- Separated
- In a domestic partnership or civil union
- Single, but cohabiting with a significant other
- Single, never married

3. What is your gender?

- Female
- Male

4. What is the highest level of education you have completed?

5. Which of the following categories best describes your employment status?

- Employed, working full-time
- Employed, working part-time
- Not employed, looking for work
- Full-time student
- Not employed, NOT looking for work
- Retired
- Disabled, not able to work

6. In what country do you currently reside?

- United States
- Other (please specify)

7. Which race/ethnicity best describes you? (Please choose only one.)

- American Indian or Alaskan Native
- Asian / Pacific Islander
- Black or African American
- Hispanic American
- White / Caucasian
- Prefer not to answer
- Multiple ethnicity / Other (please specify)

8. In what year were you born? (enter 4-digit birth year; for example, 1976)

9. How many people currently live in your household (including you)?

10. Are you co-habiting with a partner or spouse?

- Yes
- No
- I don't have a partner or a spouse

11. Do you currently have a roommate (someone with whom you live, and who is not your spouse or partner)?

- Yes
- No

12. How much total combined money did all members of your HOUSEHOLD earn last year?

- $0 to $9,999
- $10,000 to $24,999
- $25,000 to $49,999
- $50,000 to $74,999
- $75,000 to $99,999
- $100,000 to $124,999
- $125,000 to $149,999
- $150,000 to $174,999
- $175,000 to $199,999
- $200,000 and up
- Prefer not to answer

13. How many children are you parent or guardian for and live in your household (aged 17 or younger only)?

- ○ None
- ○ 1
- ○ 2
- ○ 3
- ○ 4
- ○ More than 4
- ○ Prefer not to answer

THE UNIVERSITY OF CHICAGO

14. Do you have at least monthly contact (including face-to-face, telephone, or written/email contact) with your children?

- Yes
- No
- I don't have children

15. Do you have at least montly contact (including face-to-face, telephone, or written/email contact) with other family?

- Yes
- No
- I don't have other family

16. Do you have at least monthly contact (including face-to-face, telephone, or written/email contact) with friends?

- Yes
- No
- I don't have friends

17. During the past week, how often did you feel that you were "in tune" with the people around you?

Never	Rarely	Sometimes	Always
○	○	○	○

18. During the past week, how often did you feel that you lacked companionship?

Never	Rarely	Sometimes	Always
○	○	○	○

19. During the past week, how often did you feel that there were people you could talk to?

Never	Rarely	Sometimes	Always
○	○	○	○

20. During the past week, how often did you feel part of a group of friends?

Never	Rarely	Sometimes	Always
○	○	○	○

21. During the past week, how often did you feel that you had a lot in common with the people around you?

Never	Rarely	Sometimes	Always
○	○	○	○

22. During the past week, how often did you feel close to people?

Never	Rarely	Sometimes	Always
○	○	○	○

23. During the past week, how often did you feel left out?

Never	Rarely	Sometimes	Always
○	○	○	○

24. During the past week, how often did you feel isolated from others?

Never	Rarely	Sometimes	Always
○	○	○	○

25. During the past week, how often did you feel that there were people you could turn to?

Never	Rarely	Sometimes	Always
○	○	○	○

Social Distance Project

BEFORE ASKING YOU QUESTIONS ABOUT YOUR SOCIAL CONNECTIONS, LET US TELL YOU MORE ABOUT WHAT WE KNOW ABOUT SOCIAL CONNECTIONS. OUR CONNECTIONS WITH OTHER PEOPLE CAN INCLUDE 4 CIRCLES:

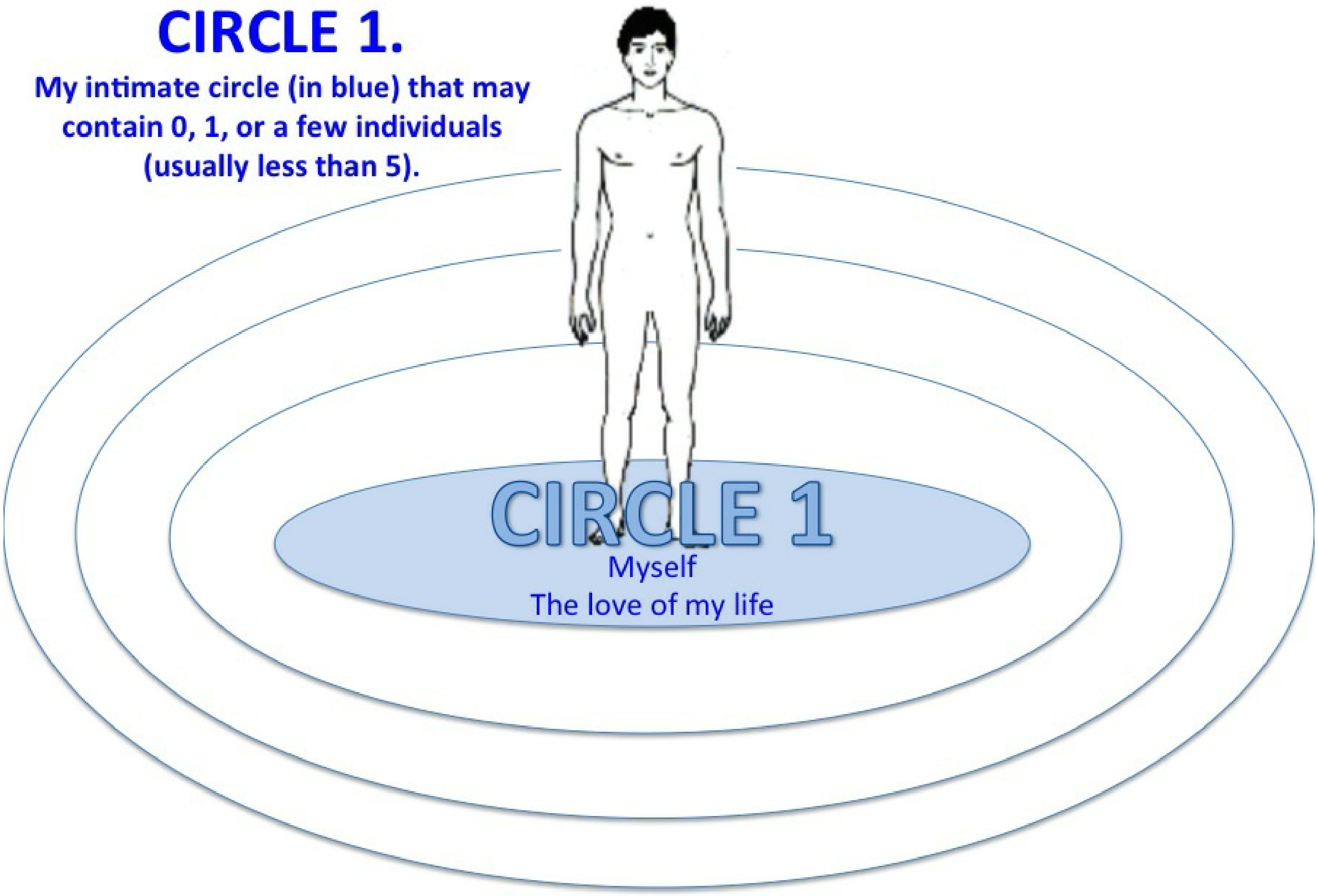

My intimate circle includes people I consider intimate (e.g., Myself, The love of my life).
People in whom I can confide and who can confide in me, or people whose love means everything to me.

CIRCLE 2.

My relational circle (in blue)

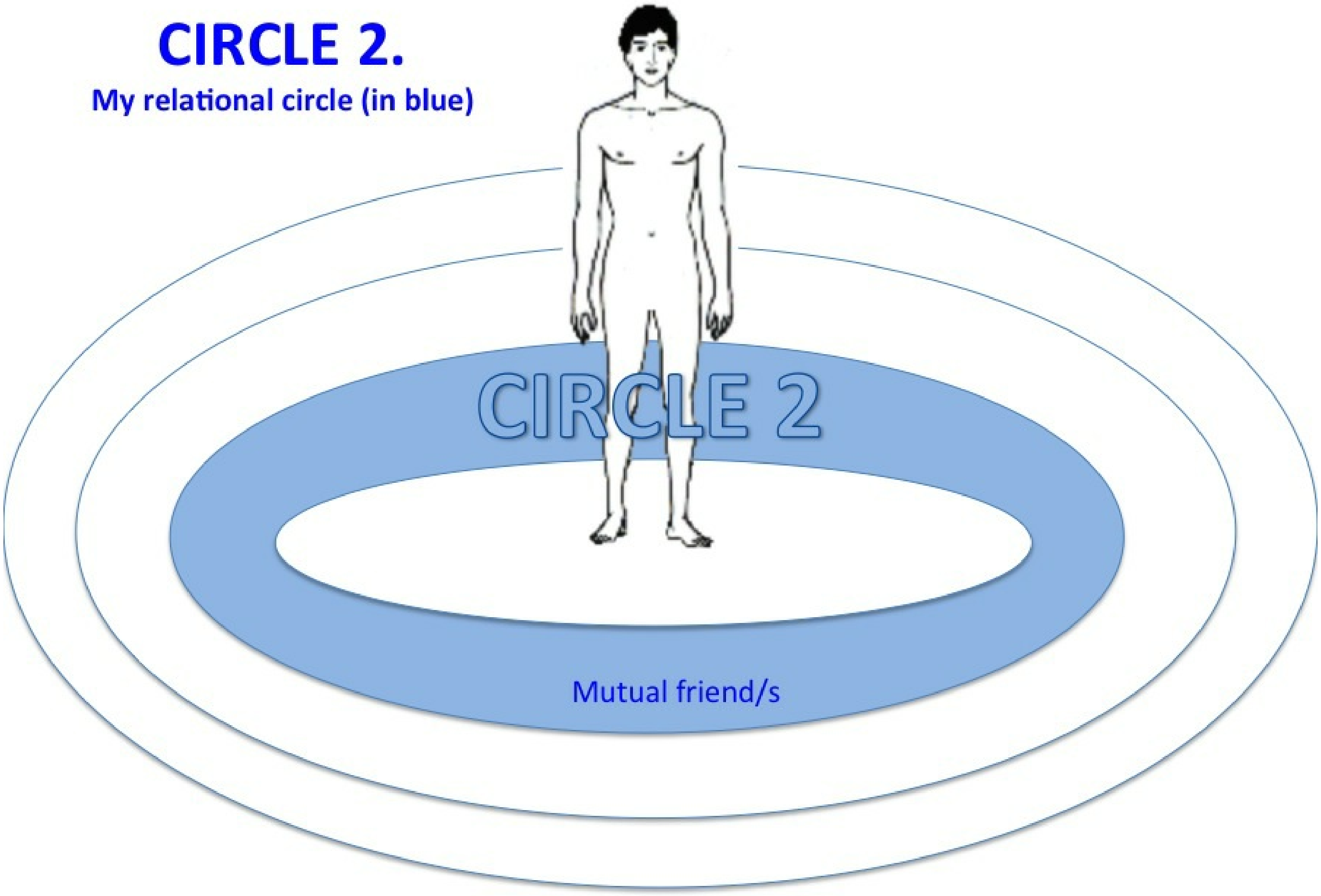

My relational circle includes people I consider being part of my life, people who are friends or family members that I can trust for protection and assistance and who can similarly trust me.

Note: A mutual friend is a friend I like, and a friend who likes me as well.

CIRCLE 3.

My collective circle (in blue)

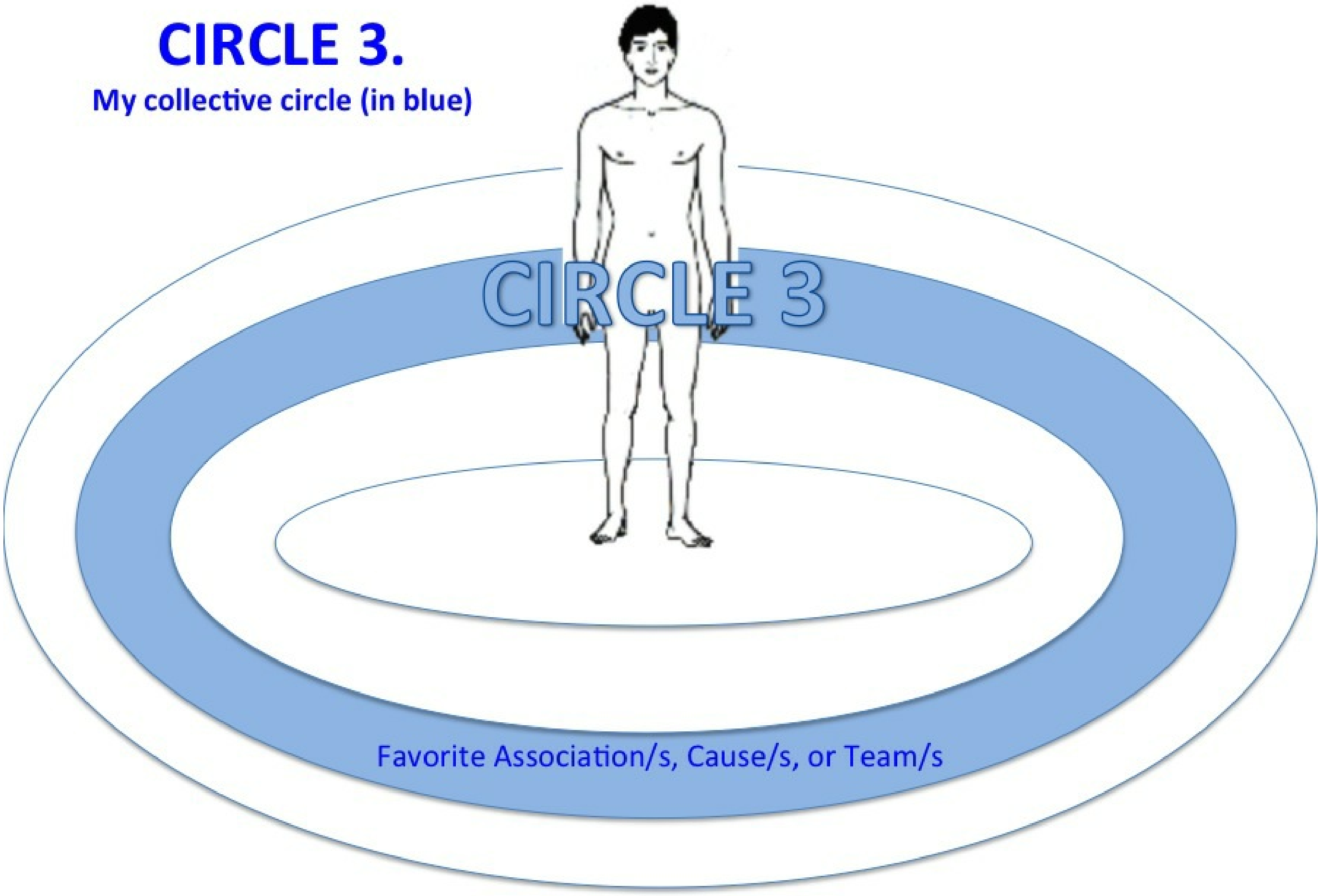

My collective circle includes "social identities" or groups with which I identify – such as a sport team, an occupational association, religious group, social group, or political party with which I identify. I feel connected to the people in this sector because of a shared group identity.

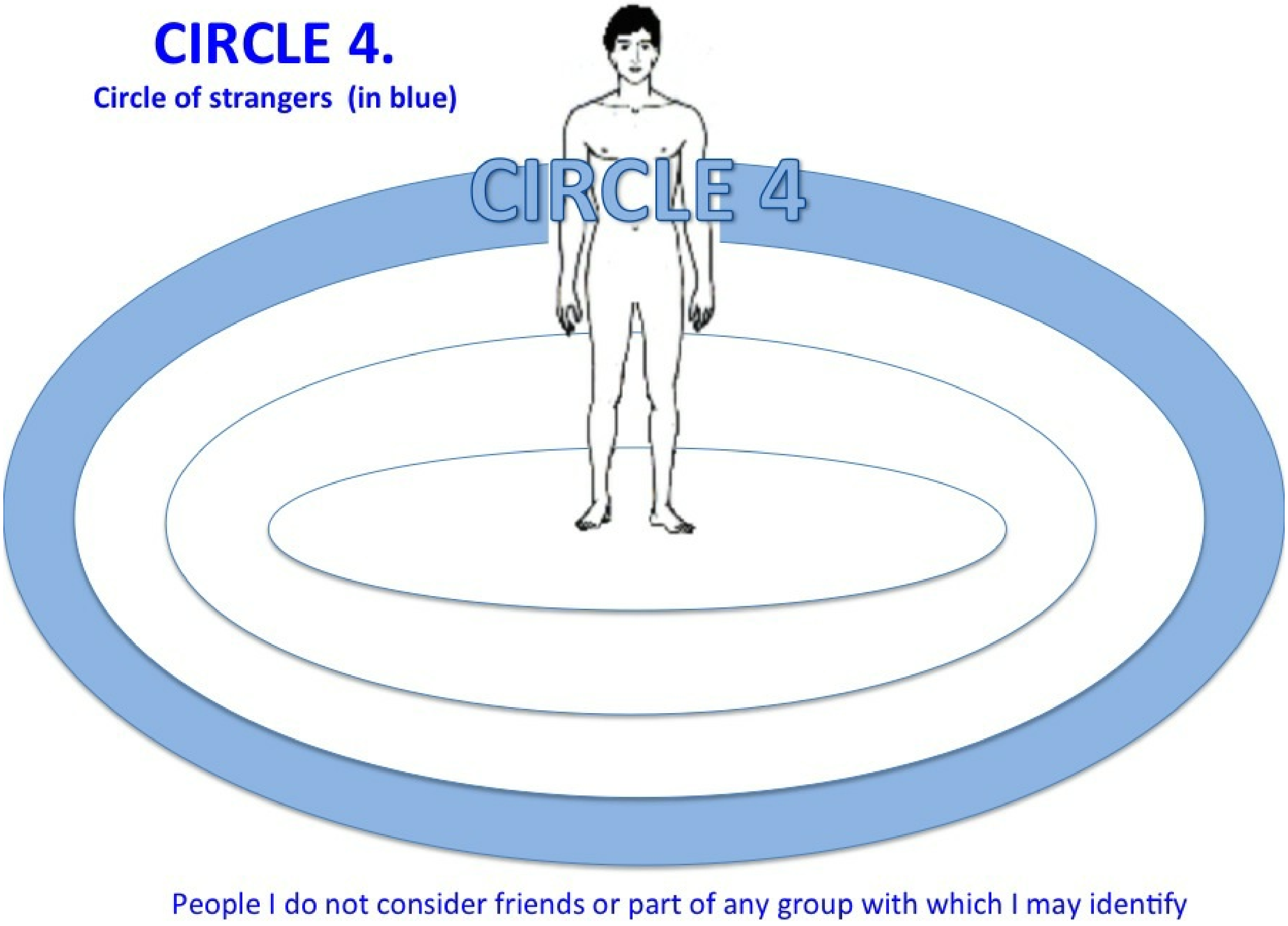

NOW CLICK NEXT TO ANSWER QUESTIONS ABOUT YOUR SOCIAL CIRCLES.

THE UNIVERSITY OF CHICAGO

QUESTIONS ABOUT YOUR INTIMATE CIRCLE

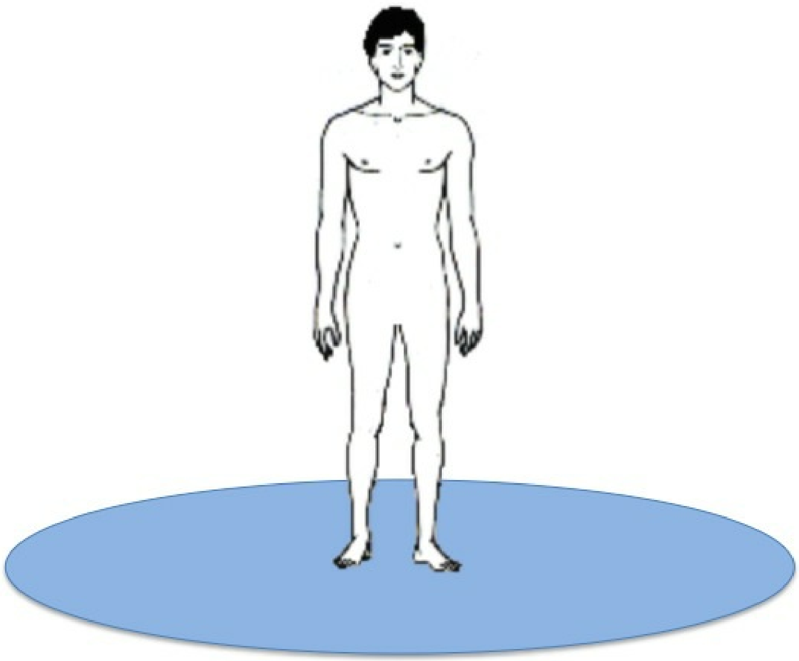

THE UNIVERSITY OF CHICAGO

Social Distance Project

26. How many people do you include in your intimate circle?

27. What are their roles?

- [ ] Spouse or partner
- [ ] Grand-parent/s
- [ ] Mother
- [ ] Father
- [ ] Brother/s
- [ ] Sister/s
- [ ] Best friend/s
- [ ] Other (please specify)

28. If a member in your intimate circle were to be sitting or standing next to you, how close would that individual have to get for you to make you feel uncomfortable?

- ○ No distance would make me feel unconfortable
- ○ 1-9 inches would begin to feel uncomfortable
- ○ 10-25 inches would begin to feel uncomfortable
- ○ 2.5 feet would begin to feel uncomfortable
- ○ 5 feet would begin to feel uncomfortable
- ○ More than 5 feet would make me feel uncomfortable
- ○ I have no one in my intimate circle
- ○ Prefer not to answer

29. How far would you travel for a member of your intimate circle?

- ○ 0, I would not travel for a member of my intimate circle.
- ○ Up to 10 miles
- ○ Up to 50 miles
- ○ Up to 100 miles
- ○ Up to 500 miles
- ○ Up to 3000 miles
- ○ Up to 7000 miles
- ○ Up to 25000 miles
- ○ No limit
- ○ I have no one in my intimate circle
- ○ Prefer not to answer

30. During the past week, how often did you feel that you lacked companionship in your intimate circle ?

Never	Rarely	Sometimes	Always	I have no one in my intimate circle
○	○	○	○	○

31. How did that make you feel?

Very happy, not hurt	Hurts just a little bit	Hurts a little more	Hurts even more	Hurts a whole lot	Hurts as much as you can imaging
○	○	○	○	○	○

32. During the past week, how often did you feel rejected by or distant from someone in your intimate circle ?

Never	Rarely	Sometimes	Always	I have no one in my intimate circle
○	○	○	○	○

33. How did that make you feel?

Very happy, not hurt	Hurts just a little bit	Hurts a little more	Hurts even more	Hurts a whole lot	Hurts as much as you can imaging
○	○	○	○	○	○

QUESTIONS ABOUT YOUR RELATIONAL CIRCLE

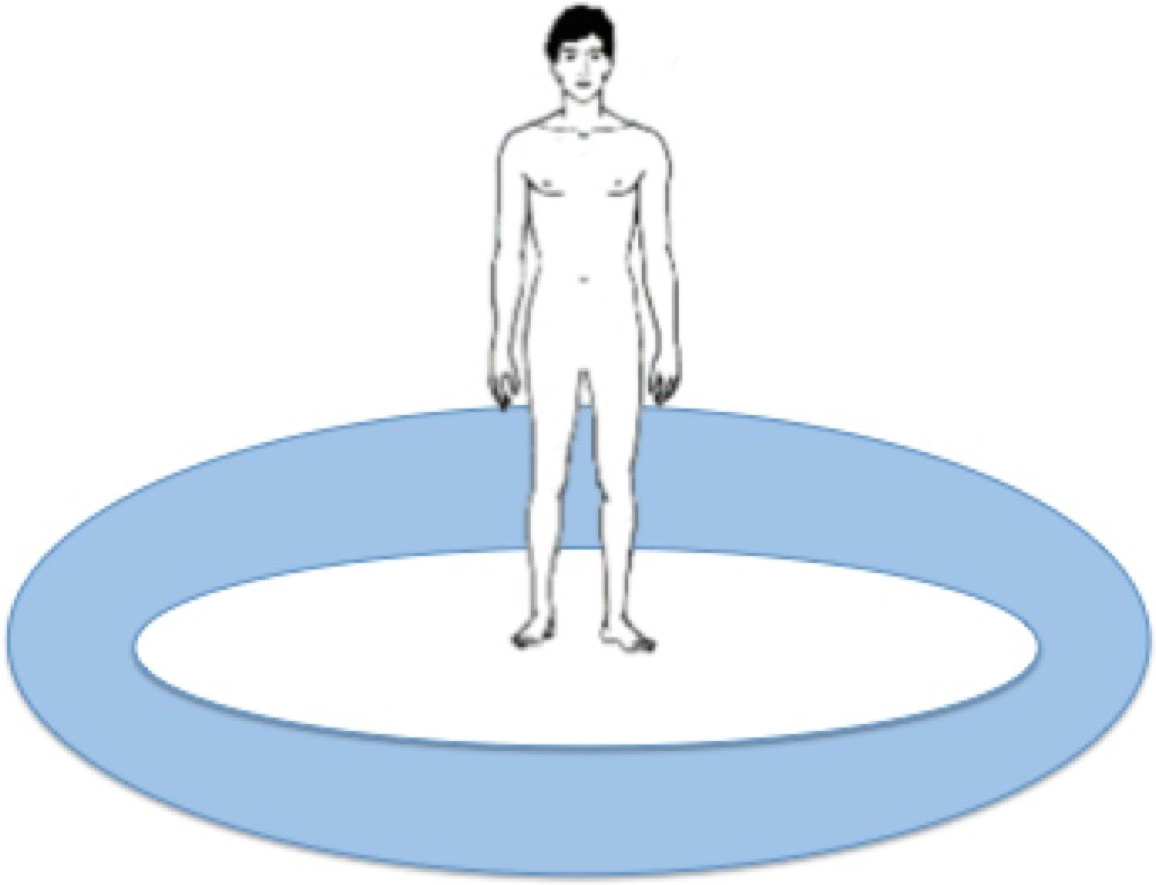

34. How many people do you include in your RELATIONAL circle?

35. What are their roles?

- Spouse or partner
- Grand-parent/s
- Mother
- Father
- Brother/s
- Sister/s
- Best friend/s
- Other (please specify)

36. If a member in your RELATIONAL circle were to be sitting or standing next to you, how close would that individual have to get for you to make you feel uncomfortable?

- No distance would make me feel unconfortable
- 1-9 inches would begin to feel uncomfortable
- 10-25 inches would begin to feel uncomfortable
- 2.5 feet would begin to feel uncomfortable
- 5 feet would begin to feel uncomfortable
- More than 5 feet would make me feel uncomfortable
- I have no one in my relational circle
- Prefer not to answer

37. How far would you travel for a member of your RELATIONAL circle?

- 0, I would not travel for a member of my relational circle.
- Up to 10 miles
- Up to 50 miles
- Up to 100 miles
- Up to 500 miles
- Up to 3000 miles
- Up to 7000 miles
- Up to 25000 miles
- No limit
- I have no one in my relational circle
- Prefer not to answer

38. During the past week, how often did you feel that you lacked companionship in your RELATIONAL circle ?

Never	Rarely	Sometimes	Always	I have no one in my relational circle
○	○	○	○	○

39. How did that make you feel?

Very happy, not hurt	Hurts just a little bit	Hurts a little more	Hurts even more	Hurts a whole lot	Hurts as much as you can imaging
○	○	○	○	○	○

40. During the past week, how often did you feel rejected by or distant from someone in your RELATIONAL circle ?

Never	Rarely	Sometimes	Always	I have no one in my relational circle
○	○	○	○	○

41. How did that make you feel?

Very happy, not hurt	Hurts just a little bit	Hurts a little more	Hurts even more	Hurts a whole lot	Hurts as much as you can imaging
○	○	○	○	○	○

QUESTIONS ABOUT YOUR COLLECTIVE CIRCLE

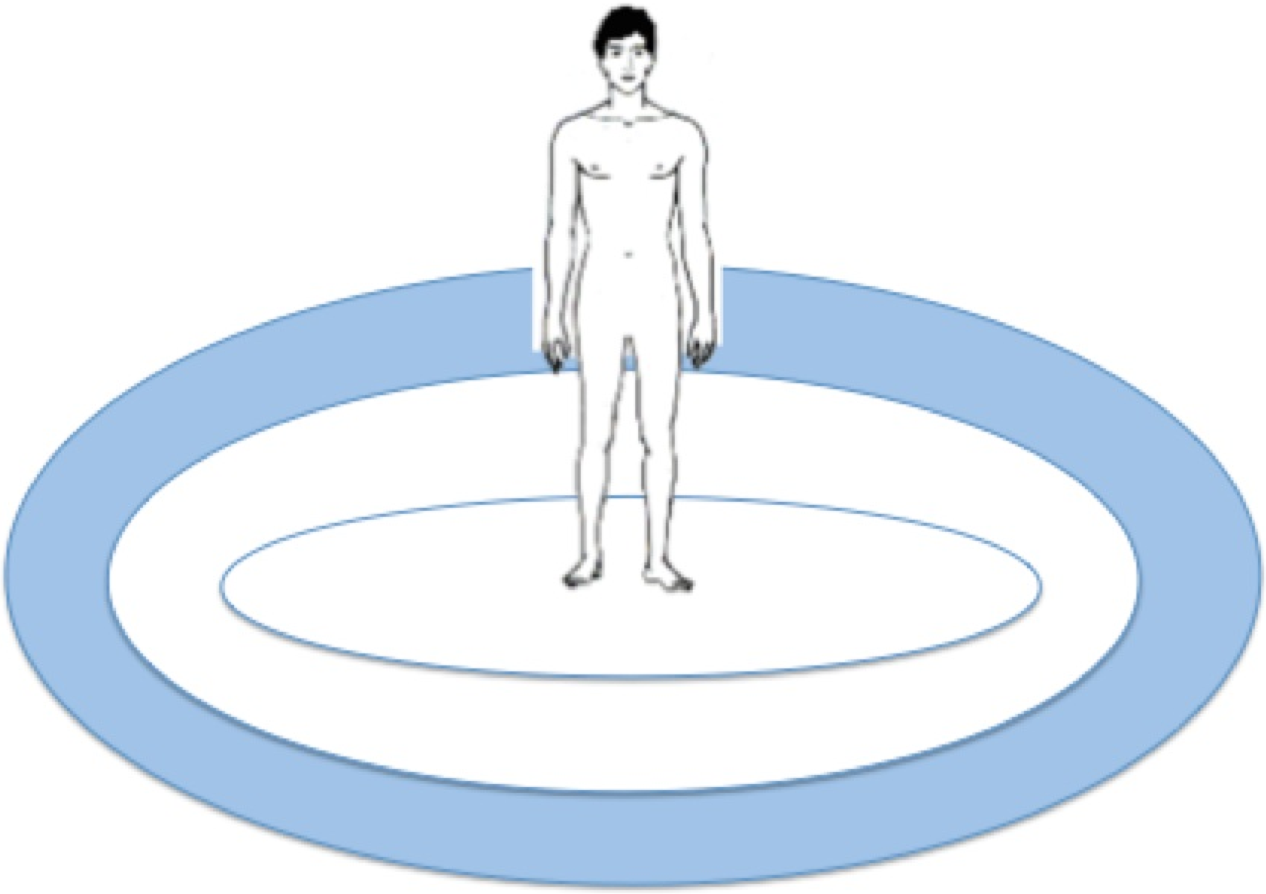

42. How many groups, teams, clubs, or associations do you include in your COLLECTIVE circle?

43. What are their roles?

- Sport team/s
- Club/s
- Professional Association/s
- Committee/s
- Religious
- Nation/s
- Other (please specify)

44. Do you actively participate in any social clubs, religious groups, associations or committees?

- Yes
- No
- Prefer not to answer

45. If a member in your COLLECTIVE circle were to be sitting or standing next to you, how close would that individual have to get for you to make you feel uncomfortable?

- No distance would make me feel unconfortable
- 1-9 inches would begin to feel uncomfortable
- 10-25 inches would begin to feel uncomfortable
- 2.5 feet would begin to feel uncomfortable
- 5 feet would begin to feel uncomfortable
- More than 5 feet would make me feel uncomfortable
- I have no one in my collective circle
- Prefer not to answer

46. How far would you travel for a member of your COLLECTIVE circle?

- 0, I would not travel for a member of my collective circle.
- Up to 10 miles
- Up to 50 miles
- Up to 100 miles
- Up to 500 miles
- Up to 3000 miles
- Up to 7000 miles
- Up to 25000 miles
- No limit
- I have no one in my collective circle
- Prefer not to answer

THE UNIVERSITY OF CHICAGO

Social Distance Project

47. During the past week, how often did you feel that you lacked companionship in your COLLECTIVE circle ?

Never	Rarely	Sometimes	Always	I have no one in my collective circle
○	○	○	○	○

48. How did that make you feel?

Very happy, not hurt	Hurts just a little bit	Hurts a little more	Hurts even more	Hurts a whole lot	Hurts as much as you can imaging
○	○	○	○	○	○

49. During the past week, how often did you feel rejected by or distant from someone in your COLLECTIVE circle ?

Never	Rarely	Sometimes	Always	I have no one in my collective circle
○	○	○	○	○

50. How did that make you feel?

Very happy, not hurt	Hurts just a little bit	Hurts a little more	Hurts even more	Hurts a whole lot	Hurts as much as you can imaging
○	○	○	○	○	○

QUESTIONS ABOUT YOUR CIRCLE OF STRANGERS (I.E., WITH EVERYONE ELSE)

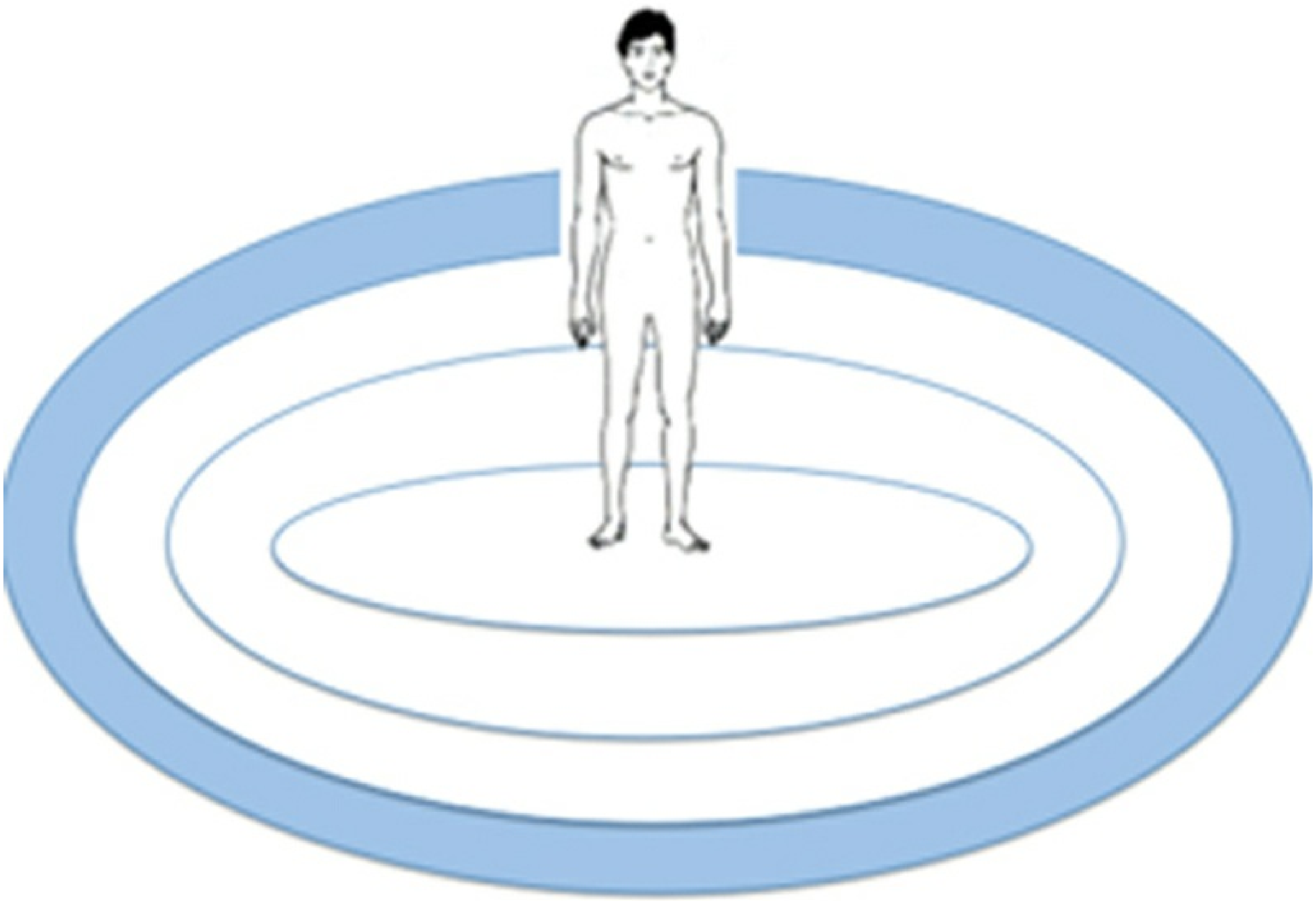

THE UNIVERSITY OF CHICAGO

Social Distance Project

51. How many people do you include in your circle of STRANGERS?

52. If a member in your circle of STRANGERS were to be sitting or standing next to you, how close would that individual have to get for you to make you feel uncomfortable?

- No distance would make me feel unconfortable
- 1-9 inches would begin to feel uncomfortable
- 10-25 inches would begin to feel uncomfortable
- 2.5 feet would begin to feel uncomfortable
- 5 feet would begin to feel uncomfortable
- More than 5 feet would make me feel uncomfortable
- I have no one in my circle of strangers
- Prefer not to answer

53. How far would you travel for a member of your circle of STRANGERS?

- 0, I would not travel for a member of my circle of strangers.
- Up to 10 miles
- Up to 50 miles
- Up to 100 miles
- Up to 500 miles
- Up to 3000 miles
- Up to 7000 miles
- Up to 25000 miles
- No limit
- I have no one in my circle of strangers
- Prefer not to answer

54. During the past week, how often did you feel that you lacked companionship in your circle of STRANGERS?

Never	Rarely	Sometimes	Always	I have no one in my circle of strangers

55. How did that make you feel?

Very happy, not hurt	Hurts just a little bit	Hurts a little more	Hurts even more	Hurts a whole lot	Hurts as much as you can imaging
○	○	○	○	○	○

56. During the past week, how often did you feel rejected by or distant from someone in your circle of STRANGERS?

Never	Rarely	Sometimes	Always	I have no one in my circle of strangers
○	○	○	○	○

57. How did that make you feel?

Very happy, not hurt	Hurts just a little bit	Hurts a little more	Hurts even more	Hurts a whole lot	Hurts as much as you can imaging
○	○	○	○	○	○

YOU ARE ALMOST DONE!! NOW LET US ASK YOU A FEW QUESTIONS ABOUT YOUR GENERAL MOOD OVER THE PAST WEEK.

58. During the past week, how often did you feel tense or wound up?

Not at all	Time to time	A lot of the time	Most of the time
○	○	○	○

59. During the past week, have you still enjoyed the things you used to enjoy?

Hardly at all	Only a little	Not quite so much	Definitely as much
○	○	○	○

60. During the past week, did you get a sort of frightened feeling as if something awful was about to happen?

Not al all	A little, but it doesn't worry me	Yes, but not too badly	Very definitely and quite badly
○	○	○	○

61. During the past week, did you laugh and see the funny side of things?

Not al all	Definitely not so much now	Not quite as much now	As much as I always could
○	○	○	○

62. During the past week, did you have worrying thoughts going through your mind?

A great deal of time	A lot of time	From time to time but not too often	Only occasionally
○	○	○	○

63. During the past week, did you feel cheerful?

Not at all	Not often	Sometimes	Most of the time
○	○	○	○

64. During the past week, were you able to sit at ease and feel relaxed?

Definitely	Usually	Not often	Not at all
○	○	○	○

65. During the past week, did you feel as if you were slowed down?

Nearly all the time	Very often	Sometimes	Not at all
○	○	○	○

66. During the past week, did you get a sort of frightened feeling like butterflies in the stomach?

Not at all	Occasionally	Quite often	Very often
○	○	○	○

67. During the past week, have you lost interest in your appearance?

Definitely	I don't take so much care as I should	I may not take quite as much care	I take just as much care as ever
○	○	○	○

68. During the past week, have you felt restless as if you had to be on the move?

Not at all	Not very much	Quite a lot	Very much indeed
○	○	○	○

69. During the past week, have you looked forward with enjoyment to things?

As much as I ever did	Rather less than I used to	Definitely less than I used to	Hardly at all
○	○	○	○

70. During the past week, did you get sudden feelings of panic?

Not at all	Not very often	Quite often	Very often indeed
○	○	○	○

71. During the past week, were you able to enjoy a good book or radio or TV programme?

Often	Sometimes	Not often	Very seldom
○	○	○	○
