# Supplementary material for: Loneliness predicts a preference for larger interpersonal distance within intimate space
Source: PLoS One. 2018 Sep 6;13(9):e0203491. doi: 10.1371/journal.pone.0203491 (PMC6126853; doi:10.1371/journal.pone.0203491)
Supplement: S2 Survey — (PDF) [file pone.0203491.s003.pdf]

mTurk ID

Please re-enter your MTurk worker ID (this must be accurate in order to receive compensation):

Consent

Hello:

You are being asked to participate in a research study on social connections that has been approved by the University of Chicago Institutional Review Board.

This page describes the study procedures, the risks and benefits of participation, as well as how your confidentiality will be maintained.

Please take your time to read this document and feel comfortable making a decision whether to participate or not. This process is called informed consent.

WHY IS THIS STUDY BEING DONE? The purpose of this research study is to better understand social connections worldwide through a series of approximately 130 questions. Through these questions, the investigators hope that it will become possible for them to better characterize the current landscape of social connections.

HOW MANY PEOPLE WILL TAKE PART IN THE STUDY? In order to best grasp the current social landscape, we are hoping to test as many participants as possible. We are accepting US-residents only as participants for the study at this time. Also, we can only accept one response from each participant. Please do not take this survey more than once.

WHAT IS INVOLVED IN THE STUDY? During this study, you will be asked to answer approximately 130 questions. Filling out these 130 questions tends to take approximately 20-25 minutes. 10 OF THESE QUESTIONS ARE ATTENTION CHECKS AND/OR COMPREHENSION QUESTIONS. YOU MUST GET AT LEAST 8 OUT OF 10 OF THESE QUESTIONS CORRECT IN ORDER TO RECEIVE COMPENSATION FOR THIS STUDY.

WHAT ARE THE RISKS OF THE STUDY?
Likely Risks: • General stiffness and back discomfort from remaining in one position • Some people become anxious while answering questions. This anxiety is more likely for those who tend to feel uncomfortable or fearful when answering demographic questions. If you feel uncomfortable in answering some questions, you can skip the question or select the answer "Prefer not to answer" when applicable.

Less Likely Risks: • There is a possible risk of confidentiality loss associated with this study. Although we are not collecting your name or any other identifiers, your IP (Internet Protocol) address is automatically stored in online surveys. We will help protect your confidentiality by keeping all data in locked filing cabinets and password-protected computers. These data will only be available to the research team.

WHAT ABOUT CONFIDENTIALITY? This survey is anonymous. No identifiers will be collected, except for your mTurk worker ID for compensation purposes. Please be aware that any work performed on Amazon MTurk can potentially be linked to information about you on your Amazon public profile page, depending on the settings you have for your Amazon profile. We will not be accessing any personally identifying information about you that you may have put on your Amazon public profile page. We will store your mTurk worker ID separately from the other information you provide to us. If you decide to participate, you will also be assigned a unique participant number. The analysis of your responses will be performed with this code number in the data field where a name would ordinarily go. Your name or any other personal information will not be recorded. After the survey, all responses will be kept in a password protected computer, and will only be accessible to investigators. Data from this study may be used in research publications or presentations.

ARE THERE ANY BENEFITS TO TAKING PART IN THE STUDY? Your participation in this study will have NO direct benefit to you personally. (Compensation procedures are described below.)

WHAT OTHER OPTIONS ARE THERE? You may choose not to participate. The decision whether or not you wish to participate in this study will not affect your participation in future psychology studies.

WHAT ARE THE COSTS? There are no monetary costs to you for participating in this study.

WILL I BE PAID FOR MY PARTICIPATION? You will be paid a small remuneration of $3 for your participation, if you successfully complete the survey. Your participation is VOLUNTARY. You may withdraw participation at any time. Upon successful completion of the survey, you will receive a code to redeem a $3 remuneration for your time via Amazon Mechanical Turk. To receive payment for participating, you will need to click "Accept HIT" in the Mechanical Turk window, enter the validation code provided, and then click "Submit." PLEASE NOTE, YOU WILL NOT RECEIVE A VALIDATION CODE OR COMPENSATION IF YOU MISS MORE THAN 2 OF THE 10 ATTENTION/COMPREHENSION CHECK QUESTIONS.

WHAT ARE MY RIGHTS AS A PARTICIPANT? Taking part in this study is voluntary. You may choose not to participate at any time during the study. If you choose to no longer be in the study, your participation will be immediately disregarded. If you start to take this survey and then change your mind, you can close the browser -- we will not use any data about you if you withdraw from the survey.

WHO DO I CALL IF I HAVE QUESTIONS OR PROBLEMS?

If you have further questions about the study, you may call the principal investigator of this study, Dr. Stephanie Cacioppo, at 773-702-1962.

If you have any questions concerning your rights as a participant in this research study or if you have any concerns about this research study, you may contact the University of Chicago's Social and Behavioral Institutional Review Board (IRB), 1155 E 60th Street, Room 414, Chicago, IL 60637, which is concerned with the protection of human subjects in research projects. You may reach the Committee office between 8:00 am and 5:00 pm, Monday through Friday, by calling (773) 702-5064, emailing sbs-irb@uchicago.edu, or writing to the University of Chicago's Socia and Behavioral Institutional Review Board (IRB), 1155 E 60th Street, Room 414, Chicago, IL 60637, USA.

CONSENT FORM

I understand the research project and the procedures associated with it. The experimental procedures have been identified and no guarantee has been given about the possible results. I am at least 18 years old and I agree to participate in this study. This consent form document does not have an expiration date. My participation is voluntary. I can answer "No" in this form if I do not want to be part of this research study.

- Yes
- No

These page timer metrics will not be displayed to the recipient.

First Click: *0 seconds.*
Last Click: *0 seconds.*
Page Submit: *0 seconds.*
Click Count: *0 clicks.*

Screen-Out 2

In what country do you currently reside?

- United States
- Other (please specify)

To the best of your knowledge, have you taken this survey before?

- No, I have not taken this survey before
- Yes, I have taken this survey before

Main Survey - Block 1

What is your gender?

- Female
- Male

Which of the following best describes your current relationship status?

- Married
- Widowed
- Divorced
- Separated
- In a domestic partnership or civil union
- Single, but cohabiting with a significant other
- Single, never married

What is the highest level of education you have completed?

Which of the following categories best describes your employment status?

- Retired

Full-time Student

- Employed, working part-time
- Not employed, looking for work
- Disabled, not able to work
- Employed, working full-time
- Not employed, NOT looking for work
- Prefer Not to Answer

Which race/ethnicity best describes you? (Please choose only one.)

- American Indian or Alaskan Native
- Asian / Pacific Islander
- Black or African American
- Hispanic American
- White / Caucasian
- Prefer not to answer

These page timer metrics will not be displayed to the recipient.
First Click: *0 seconds.*
Last Click: *0 seconds.*
Page Submit: *0 seconds.*
Click Count: *0 clicks.*

In what year were you born? (enter 4-digit birth year; for example, 1976)

How many people currently live in your household (including you)?

Are you cohabiting with a partner or spouse?

- Yes, I have a partner or spouse and am cohabiting with him or her
- No, I have a partner or spouse but am not cohabiting with him or her
- I don't have a partner or a spouse

Do you currently have a roommate (someone with whom you live, and who is not your spouse or partner)?

- Yes
- No

How much total combined money did all members of your HOUSEHOLD earn last year?

- $0 to $9,999
- $10,000 to $24,999
- $25,000 to $49,999
- $50,000 to $74,999
- $75,000 to $99,999
- $100,000 to $124,999
- $125,000 to $149,999
- $150,000 to $174,999
- $175,000 to $199,999

- $200,000 and up
- Prefer not to answer

How many children are you parent or guardian for and live in your household (aged 17 or younger only)?

- None
- 1
- 2
- 3
- 4
- More than 4
- Prefer not to answer

These page timer metrics will not be displayed to the recipient.
First Click: *0 seconds.*
Last Click: *0 seconds.*
Page Submit: *0 seconds.*
Click Count: *0 clicks.*

Do you have at least monthly contact (including face-to-face, telephone, or written/email contact) with your children?

- Yes
- No
- I don't have children

Do you have at least monthly contact (including face-to-face, telephone, or written/email contact) with other family?

- Yes
- No
- I don't have other family

Do you have at least monthly contact (including face-to-face, telephone, or written/email contact) with friends?

- Yes
- No
- I don't have friends

Do you actively participate in any social clubs, religious groups, associations or committees?

- Yes
- No
- Prefer not to answer

These page timer metrics will not be displayed to the recipient.
First Click: *0 seconds.*
Last Click: *0 seconds.*
Page Submit: *0 seconds.*
Click Count: *0 clicks.*

During the past week, how often did you feel that you were "in tune" with the people around you?

Never	Rarely	Sometimes	Often
○	○	○	○

During the past week, how often did you feel that you lacked companionship?

Never Rarely Sometimes Often

During the past week, how often did you feel that there were people you could talk to?

Never Rarely Sometimes Often

These page timer metrics will not be displayed to the recipient.

First Click: *0 seconds.*

Last Click: *0 seconds.*

Page Submit: *0 seconds.*

Click Count: *0 clicks.*

During the past week, how often did you feel part of a group of friends?

Never Rarely Sometimes Often

During the past week, how often did you feel that you had a lot in common with the people around you?

Never Rarely Sometimes Often

To what extent would you say you have felt isolated within the past week? Please select often.

Never Rarely Sometimes Often

During the past week, how often did you feel close to people?

Never Rarely Sometimes Often

These page timer metrics will not be displayed to the recipient.

First Click: *0 seconds.*

Last Click: *0 seconds.*

Page Submit: *0 seconds.*

Click Count: *0 clicks.*

During the past week, how often did you feel left out?

Never Rarely Sometimes Often

During the past week, how often did you feel isolated from others?

Never Rarely Sometimes Often

During the past week, how often did you feel that there were people you could turn to?

Never Rarely Sometimes Often

These page timer metrics will not be displayed to the recipient.

First Click: *0 seconds.*

Last Click: *0 seconds.*

Page Submit: *0 seconds.*

Click Count: *0 clicks.*

BEFORE ASKING YOU QUESTIONS ABOUT YOUR SOCIAL

CONNECTIONS, LET US TELL YOU MORE ABOUT WHAT WE KNOW

ABOUT SOCIAL NETWORKS. OUR CONNECTIONS WITH OTHER PEOPLE CAN INCLUDE 4 CIRCLES:

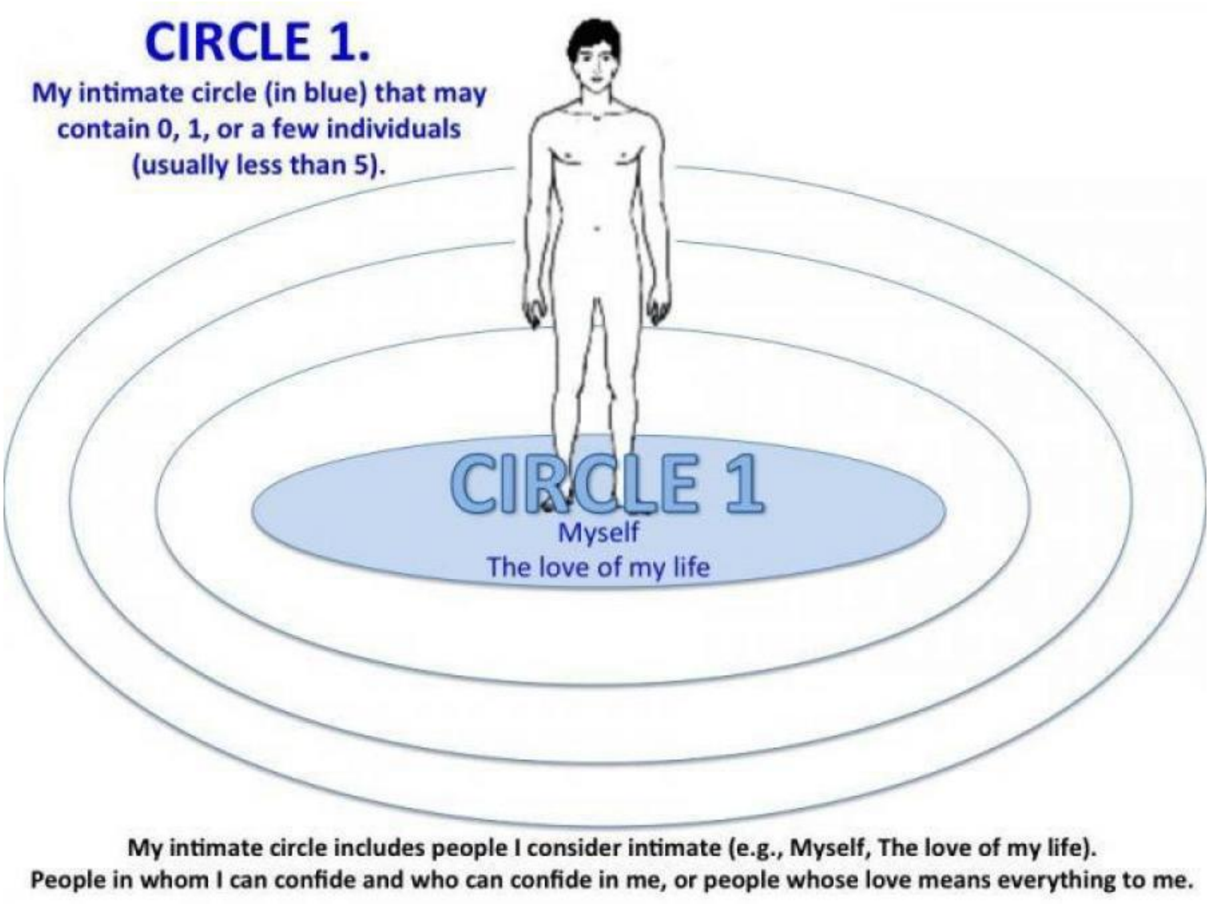

These page timer metrics will not be displayed to the recipient.
First Click: *0 seconds.*
Last Click: *0 seconds.*
Page Submit: *0 seconds.*
Click Count: *0 clicks.*

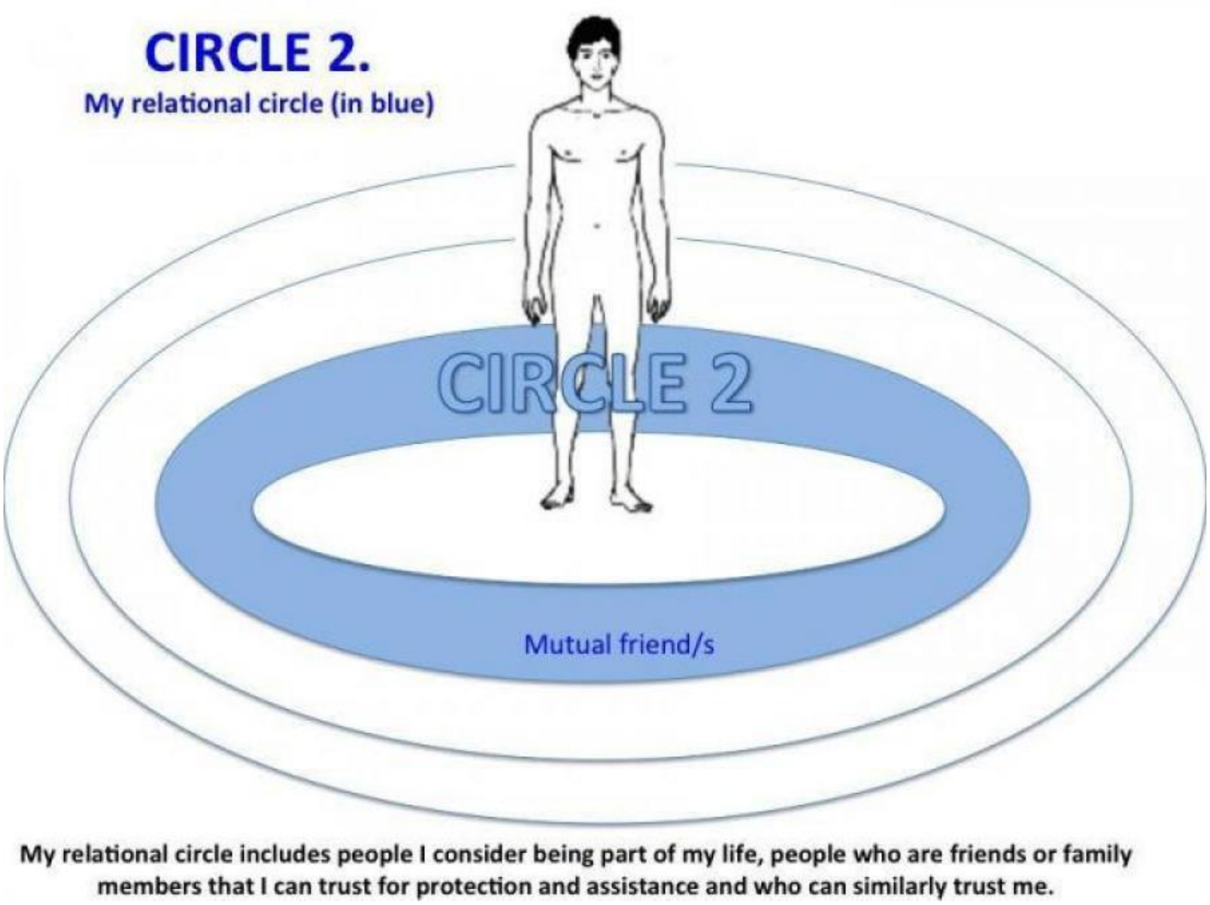

Note: A mutual friend is a friend I like, and a friend who likes me as well.

These page timer metrics will not be displayed to the recipient.
First Click: *0 seconds.*
Last Click: *0 seconds.*
Page Submit: *0 seconds.*
Click Count: *0 clicks.*

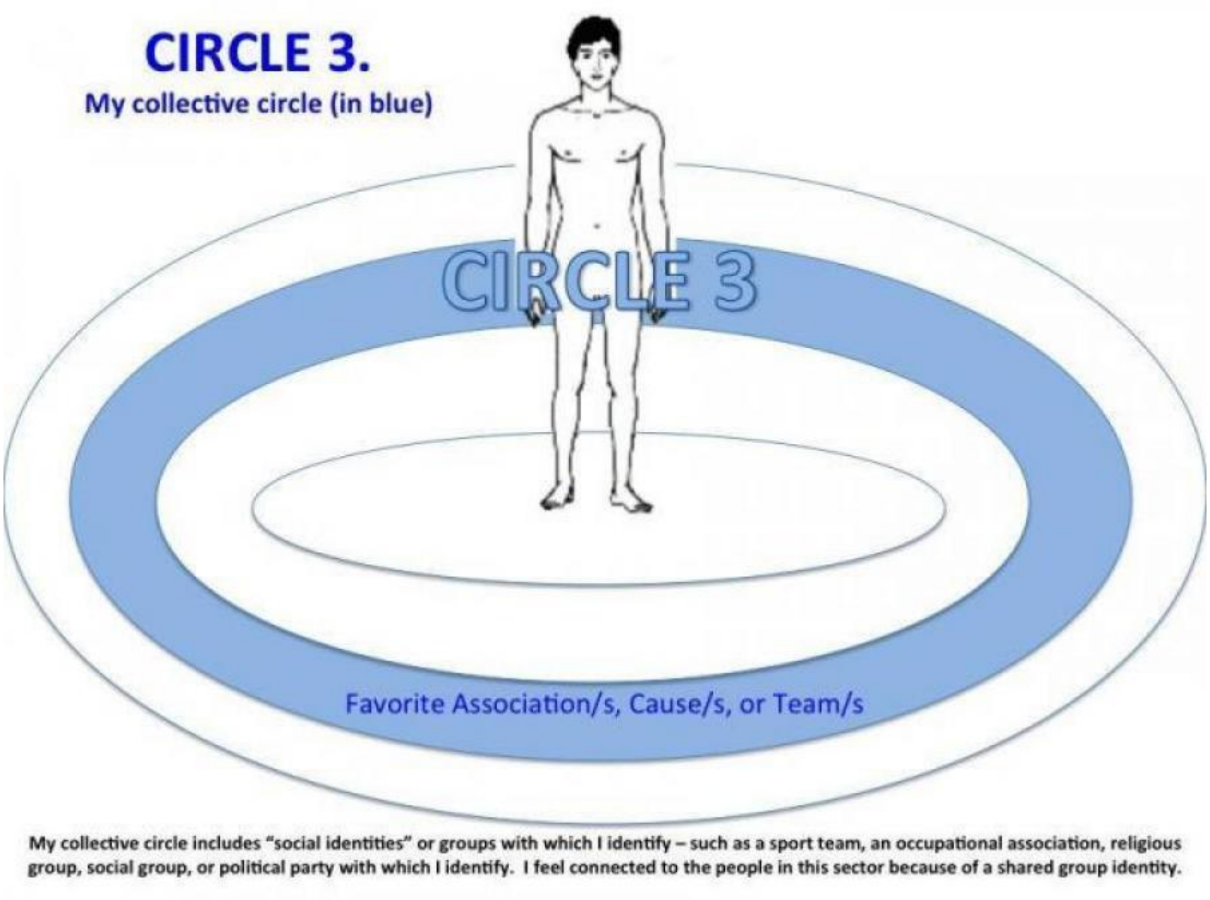

These page timer metrics will not be displayed to the recipient.
First Click: *0 seconds.*
Last Click: *0 seconds.*
Page Submit: *0 seconds.*
Click Count: *0 clicks.*

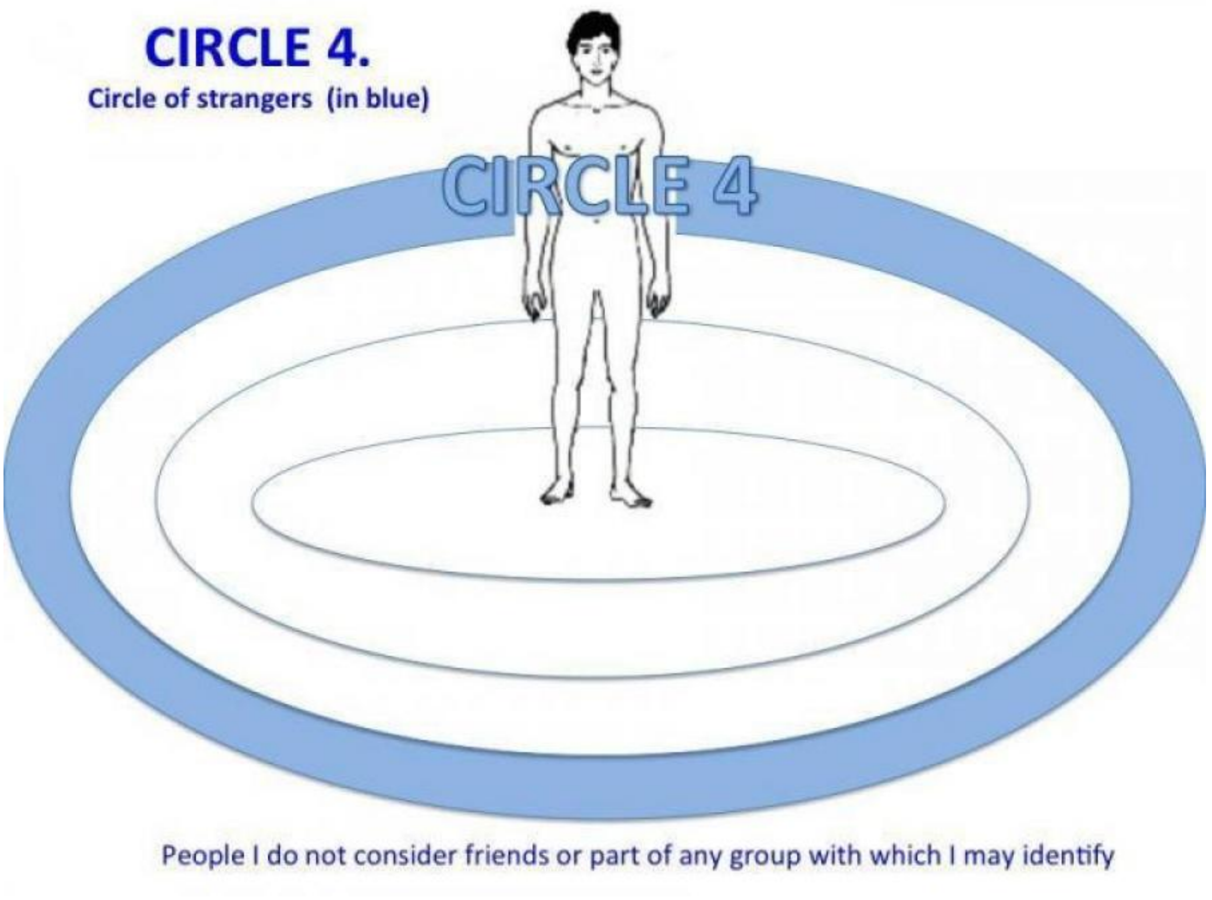

NOW CLICK NEXT TO ANSWER QUESTIONS ABOUT YOUR SOCIAL CIRCLES.

These page timer metrics will not be displayed to the recipient.
First Click: *0 seconds.*
Last Click: *0 seconds.*
Page Submit: *0 seconds.*
Click Count: *0 clicks.*

QUESTIONS ABOUT YOUR INTIMATE CIRCLE

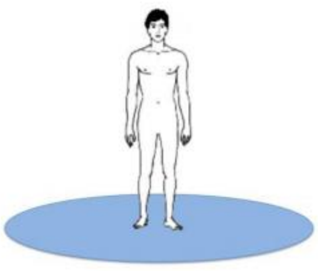

As a reminder, the intimate circle generally includes a small number of people with whom you consider yourself to be intimate, who you feel free confide in, and who are

emotionally important to you. These might include a significant other and/or close friends and family. Please do not include yourself as a member.

These page timer metrics will not be displayed to the recipient.
First Click: *0 seconds.*
Last Click: *0 seconds.*
Page Submit: *0 seconds.*
Click Count: *0 clicks.*

How many people do you include in your INTIMATE circle?

Which relationship type(s) and/or role(s) do members of your INTIMATE circle play in relation to you? (Please select all that apply.)

- Spouse or Partner
- Parent/s
- Grandparent/s
- Sibling/s
- Children
- Grand Children
- Other Relative/s
- Close Friend/s
- Friend/s
- Acquaintance/s
- Co-worker/s or Colleague/s
- Teammate/s
- Club Member/s
- Professional Association Member/s
- Committee Member/s
- Religious Organization Member/s
- Fellow Student/s or Classmate/s (former or current)
- Leisure Activity Group Member/s
- Other (Please Specify)

How often do you have contact (face-to-face, telephone, written, online or via a monitor screen) with members of your INTIMATE circle on average?

Very Rarely	Rarely	Sometimes	Often	Very Often
○	○	○	○	○

Which relationship type would most commonly be associated with the INTIMATE circle?

- A distant relative
- A coworker you know a little
- A significant other
- Your landlord who you see only to pay rent

Considering your amount of contact with your INTIMATE circle, would you prefer to have:

Much less contact	Less contact	The same level of contact	More contact	Much more contact
○	○	○	○	○

These page timer metrics will not be displayed to the recipient.
First Click: *0 seconds.*
Last Click: *0 seconds.*
Page Submit: *0 seconds.*
Click Count: *0 clicks.*

Main Survey - Block 2

Please select the picture below that best describes your relationship with members of your INTIMATE circle on average:

▼

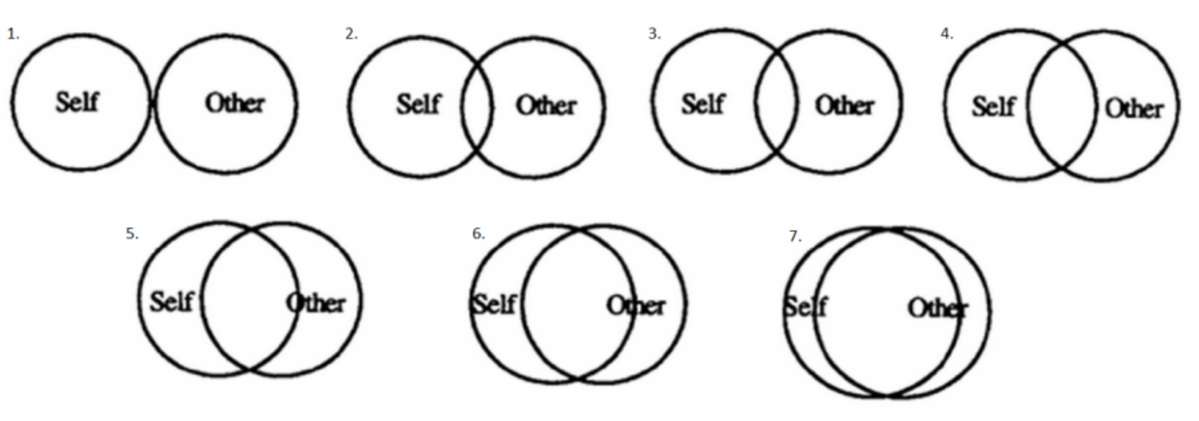

How close (psychologically / emotionally) do you feel to members of your INTIMATE circle on average?

Least Close | Close

Rate your closeness:

Considering your level of closeness to members of your INTIMATE circle, would you prefer to have:

Much less closeness	Less closeness	About the same closeness	More closeness	Much more closeness
○	○	○	○	○

How far would you travel for a member of your INTIMATE circle, select an unlimited distance?

Not very far at all	Not very far	A moderate distance	Pretty far	An unlimited distance
○	○	○	○	○

These page timer metrics will not be displayed to the recipient.

First Click: *0 seconds.*

Last Click: *0 seconds.*

Page Submit: *0 seconds.*

Click Count: *0 clicks.*

Please write the initials of a member of your INTIMATE circle who you:

[Please note that these initials will not be used for any identification purposes and are completely confidential. They will only be used to refer to specific people in later questions. Also, feel free to respond with the same initials in more than one blank--for example, if the person you have the most frequent contact is also the person you feel closest to.]

Have the MOST FREQUENT CONTACT WITH (face-to-face, telephone, written, online or via a monitor screen):

Which relationship type(s) and/or role(s) does this person play in relation to you? (Please select all that apply.)

- ☐ Spouse or Partner
- ☐ Parent/s
- ☐ Grandparent/s
- ☐ Sibling/s
- ☐ Children
- ☐ Grand Children
- ☐ Other Relative/s
- ☐ Close Friend/s
- ☐ Friend/s
- ☐ Acquaintance/s
- ☐ Co-worker/s or Colleague/s
- ☐ Teammate/s
- ☐ Club Member/s
- ☐ Professional Association Member/s
- ☐ Committee Member/s
- ☐ Religious Organization Member/s
- ☐ Fellow Student/s or Classmate/s (former or current)
- ☐ Leisure Activity Group Member/s
- ☐ Other (Please Specify)

Have the LEAST FREQUENT CONTACT WITH (face-to-face, telephone, written, online or via a monitor screen):

Which relationship type(s) and/or role(s) does this person play in relation to you? (Please select all that apply.)

- Spouse or Partner
- Parent/s
- Grandparent/s
- Sibling/s
- Children
- Grand Children
- Other Relative/s
- Close Friend/s
- Friend/s
- Acquaintance/s
- Co-worker/s or Colleague/s
- Teammate/s
- Club Member/s
- Professional Association Member/s
- Committee Member/s
- Religious Organization Member/s
- Fellow Student/s or Classmate/s (former or current)
- Leisure Activity Group Member/s
- Other (Please Specify)

Feel the CLOSEST to (psychologically / emotionally):

Which relationship type(s) and/or role(s) does this person play in relation to you? (Please select all that apply.)

- Spouse or Partner
- Parent/s
- Grandparent/s
- Sibling/s
- Children
- Grand Children
- Other Relative/s
- Close Friend/s
- Friend/s
- Acquaintance/s
- Co-worker/s or Colleague/s
- Teammate/s
- Club Member/s
- Professional Association Member/s
- Committee Member/s
- Religious Organization Member/s
- Fellow Student/s or Classmate/s (former or current)
- Leisure Activity Group Member/s
- Other (Please Specify)

Feel the LEAST CLOSE to (psychologically / emotionally):

Which relationship type(s) and/or role(s) does this person play in relation to you? (Please select all that apply.)

- Spouse or Partner
- Parent/s
- Grandparent/s
- Sibling/s
- Children
- Grand Children
- Other Relative/s
- Close Friend/s
- Friend/s
- Acquaintance/s
- Co-worker/s or Colleague/s
- Teammate/s
- Club Member/s
- Professional Association Member/s
- Committee Member/s
- Religious Organization Member/s
- Fellow Student/s or Classmate/s (former or current)
- Leisure Activity Group Member/s
- Other (Please Specify)

These page timer metrics will not be displayed to the recipient.

First Click: *0 seconds.*
Last Click: *0 seconds.*
Page Submit: *0 seconds.*

Click Count: *0 clicks.*

Block 2.1 Int Skip

Please select each set of initials you entered once (avoiding duplicates):

- ${q://QID47/ChoiceTextEntryValue}
- ${q://QID48/ChoiceTextEntryValue}
- ${q://QID49/ChoiceTextEntryValue}
- ${q://QID50/ChoiceTextEntryValue}
- I do not have anyone in my intimate circle

These page timer metrics will not be displayed to the recipient.
First Click: *0 seconds.*
Last Click: *0 seconds.*
Page Submit: *0 seconds.*
Click Count: *0 clicks.*

Block 2.2

Please answer the questions below regarding the members of your INTIMATE circle you specified on the previous page.

How often do you have contact (face-to-face, telephone, written, online or via a monitor screen) with _____?

	Very Rarely	Rarely	Sometimes	Often	Very Often
» ${q://QID47/ChoiceTextEntryValue}					
» ${q://QID48/ChoiceTextEntryValue}					
» ${q://QID49/ChoiceTextEntryValue}					
» ${q://QID50/ChoiceTextEntryValue}					
» I do not have anyone in my intimate circle					

Considering your amount of contact with _____, would you prefer to have:

	Much less contact	Less contact	The same level of contact	More contact	Much more contact
» ${q://QID47/ChoiceTextEntryValue}					
» ${q://QID48/ChoiceTextEntryValue}					
» ${q://QID49/ChoiceTextEntryValue}					
» ${q://QID50/ChoiceTextEntryValue}					
» I do not have anyone in my intimate circle					

These page timer metrics will not be displayed to the recipient.
First Click: *0 seconds.*
Last Click: *0 seconds.*
Page Submit: *0 seconds.*
Click Count: *0 clicks.*

Please select the picture below that best describes your relationship with _________.

» ${q://QID47/ChoiceTextEntryValue}
» ${q://QID48/ChoiceTextEntryValue}
» ${q://QID49/ChoiceTextEntryValue}
» ${q://QID50/ChoiceTextEntryValue}
» I do not have anyone in my intimate circle

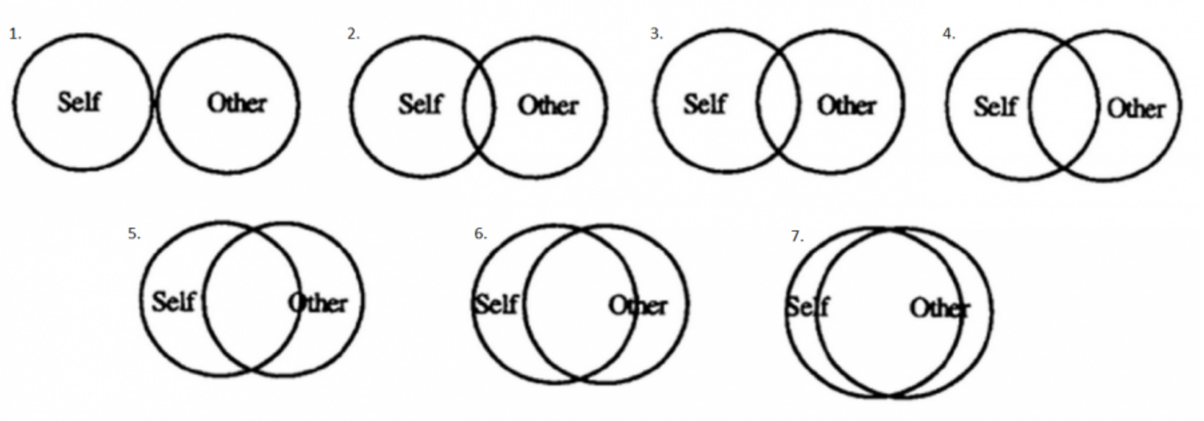

How close (psychologically / emotionally) do you feel to _____?

Least Close Close

» ${q://QID47/ChoiceTextEntryValue}	
» ${q://QID48/ChoiceTextEntryValue}	
» ${q://QID49/ChoiceTextEntryValue}	
» ${q://QID50/ChoiceTextEntryValue}	
» I do not have anyone in my intimate circle	

Considering your level of closeness to _______, would you prefer to have:

	Much less closeness	Less closeness	About the same closeness	More closeness	Much more closeness
» ${q://QID47/ChoiceTextEntryValue}	○	○	○	○	○
» ${q://QID48/ChoiceTextEntryValue}	○	○	○	○	○
» ${q://QID49/ChoiceTextEntryValue}	○	○	○	○	○
» ${q://QID50/ChoiceTextEntryValue}	○	○	○	○	○
» I do not have anyone in my intimate circle	○	○	○	○	○

These page timer metrics will not be displayed to the recipient.
First Click: *0 seconds.*
Last Click: *0 seconds.*
Page Submit: *0 seconds.*
Click Count: *0 clicks.*

Block 2.5

If a member in your INTIMATE circle were to be sitting or standing next to you, how close would that individual have to get to make you feel uncomfortable?

- ○ No distance would make me feel uncomfortable
- ○ 1-9 inches would begin to feel uncomfortable
- ○ 10-25 inches would begin to feel uncomfortable
- ○ 2.5 feet would begin to feel uncomfortable
- ○ 5 feet would begin to feel uncomfortable
- ○ More than 5 feet would begin to feel uncomfortable
- ○ I have no one in my intimate circle
- ○ Prefer not to answer

The intimate circle is often associated with close emotional intimacy and companionship. Please select strongly agree below.

Strongly Disagree	Disagree	Neither Agree or Disagree	Agree	Strongly Agree
○	○	○	○	○

Please answer the question above a second time using a different scale:

If a member in your INTIMATE circle were to be sitting or standing next to you, how close would that individual have to get for you to feel uncomfortable?

Touching   > 5 feet away (>

Please Select a Distance:	

These page timer metrics will not be displayed to the recipient.

First Click: *0 seconds.*

Last Click: *0 seconds.*

Page Submit: *0 seconds.*

Click Count: *0 clicks.*

Main Survey - Block 3

If ______ were to be sitting or standing next to you, how close would ______ have to get to make you feel uncomfortable?

	No distance would make me feel uncomfortable	1-9 inches would begin to feel uncomfortable	10-25 inches would begin to feel uncomfortable	2.5 feet would begin to feel uncomfortable	5 feet would begin to feel uncomfortable	More than 5 feet would begin to feel uncomfortable	I have no one in my intimate circle	Prefer not to answe
» ${q://QID47/ChoiceTextEntryValue}	○	○	○	○	○	○	○	○
» ${q://QID48/ChoiceTextEntryValue}	○	○	○	○	○	○	○	○
» ${q://QID49/ChoiceTextEntryValue}	○	○	○	○	○	○	○	○
» ${q://QID50/ChoiceTextEntryValue}	○	○	○	○	○	○	○	○
» I do not have anyone in my intimate circle	○	○	○	○	○	○	○	○

Please answer again using a different scale:

If ______ were to be sitting or standing next to you, how close would ______ have to get to make you feel uncomfortable?

Touching   > 5 feet (> 1.

» ${q://QID47/ChoiceTextEntryValue}	
» ${q://QID48/ChoiceTextEntryValue}	
» ${q://QID49/ChoiceTextEntryValue}	
» ${q://QID50/ChoiceTextEntryValue}	
» I do not have anyone in my intimate circle	

These page timer metrics will not be displayed to the recipient.

First Click: *0 seconds.*

Last Click: *0 seconds.*

Page Submit: *0 seconds.*

Click Count: *0 clicks.*

Block 3.9

QUESTIONS ABOUT YOUR RELATIONAL CIRCLE

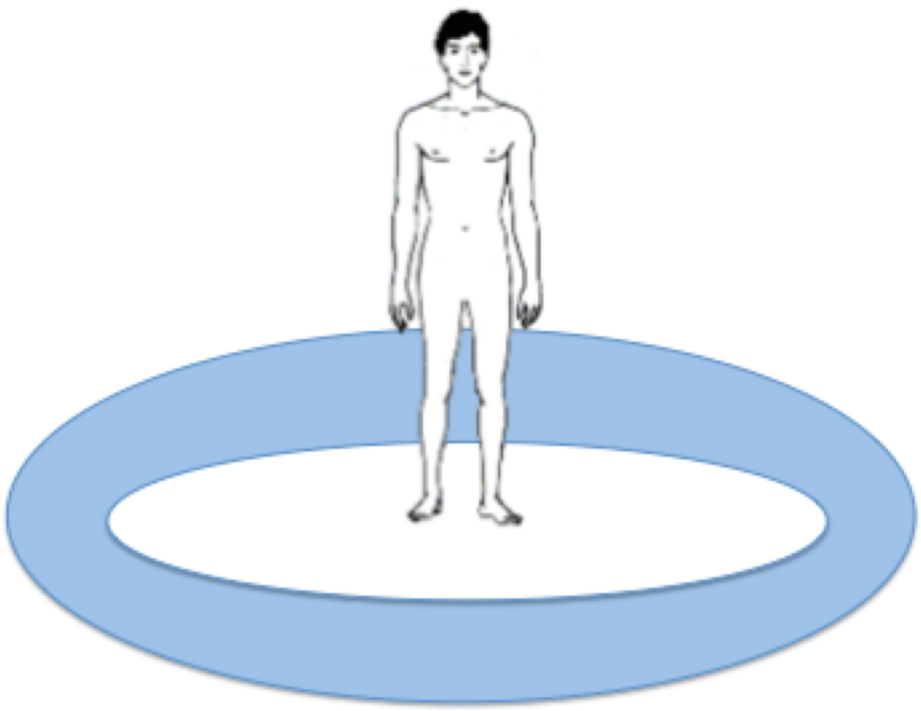

**As a reminder,
the relational circle generally includes people you consider to be part of your life who you can trust for mutual protection and assistance, yet who are somewhat less intimate than members of your intimate circle.**

These page timer metrics will not be displayed to the recipient.
First Click: *0 seconds.*
Last Click: *0 seconds.*
Page Submit: *0 seconds.*
Click Count: *0 clicks.*

How many people do you include in your RELATIONAL circle?

Which relationship type(s) and/or role(s) do members of your RELATIONAL circle play in relation to you? (Please select all that apply.)

- Spouse or Partner
- Parent/s
- Grandparent/s
- Sibling/s
- Children
- Grand Children
- Other Relative/s
- Close Friend/s
- Friend/s
- Acquaintance/s
- Co-worker/s or Colleague/s
- Teammate/s
- Club Member/s
- Professional Association Member/s
- Committee Member/s
- Religious Organization Member/s
- Fellow Student/s or Classmate/s (former or current)
- Leisure Activity Group Member/s
- Other (Please Specify)

How often do you have contact (face-to-face, telephone, written, online or via a monitor screen) with members of your RELATIONAL circle on average?

Very Rarely	Rarely	Sometimes	Often	Very Often
○	○	○	○	○

Considering your amount of contact with your RELATIONAL circle, would you prefer to have:

Much less contact	Less contact	The same level of contact	More contact	Much more contact
○	○	○	○	○

These page timer metrics will not be displayed to the recipient.
First Click: *0 seconds.*
Last Click: *0 seconds.*
Page Submit: *0 seconds.*
Click Count: *0 clicks.*

Please select the picture below that best describes your relationship with members of your RELATIONAL circle on average:

▾

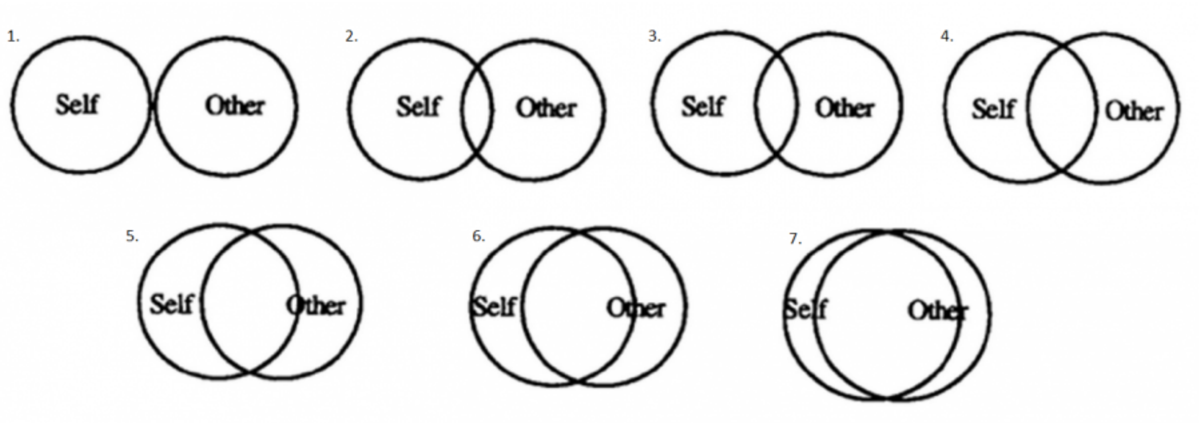

How close (psychologically / emotionally) do you feel to members of your RELATIONAL circle on average?

Least Close Close

Rate your closeness:

Considering your level of closeness to members of your RELATIONAL circle, would you prefer to have:

Much less closeness	Less closeness	About the same closeness	More closeness	Much more closeness
○	○	○	○	○

Which best describes a member/s of the relational circle?

- ○ Groups I identify with, such as sport teams or professional associations
- ○ People, such as family and friends, whom I would rely on for mutual protection and assistance
- ○ The love of my life
- ○ People I see on a daily basis but who I rarely talk to

These page timer metrics will not be displayed to the recipient.
First Click: *0 seconds.*
Last Click: *0 seconds.*
Page Submit: *0 seconds.*
Click Count: *0 clicks.*

Main Survey - Block 4

Please write the initials of a member of your RELATIONAL circle who you:
[Please note: these should NOT be people who you would include in your intimate or collective circles. Also, feel free to respond with the same initials in more than one blank--for example, if the person you have the most frequent contact is also the person you feel closest to. These initials will not be used for any identification purposes and are completely confidential. They will only be used to refer to specific people in later questions.]

Have the MOST FREQUENT CONTACT WITH (face-to-face, telephone, written, online or via a monitor screen):

Which relationship type(s) and/or role(s) does this person play in relation to you? (Please select all that apply.)

- ☐ Spanse or Partner
- ☐ Parent/s
- ☐ Grandparent/s
- ☐ Sibling/s
- ☐ Children
- ☐ Grand Children
- ☐ Other Relative/s
- ☐ Close Friend/s
- ☐ Friend/s
- ☐ Acquaintance/s
- ☐ Co-worker/s or Colleague/s
- ☐ Teammate/s
- ☐ Club Member/s
- ☐ Professional Association Member/s
- ☐ Committee Member/s
- ☐ Religious Organization Member/s
- ☐ Fellow Student/s or Classmate/s (former or current)
- ☐ Leisure Activity Group Member/s
- ☐ Other (Please Specify)

Have the LEAST FREQUENT CONTACT WITH (face-to-face, telephone, written, online or via a monitor screen):

Which relationship type(s) and/or role(s) does this person play in relation to you? (Please select all that apply.)

- ☐ Spouse or Partner
- ☐ Parent/s
- ☐ Grandparent/s
- ☐ Sibling/s
- ☐ Children
- ☐ Grand Children
- ☐ Other Relative/s
- ☐ Close Friend/s
- ☐ Friend/s
- ☐ Acquaintance/s
- ☐ Co-worker/s or Colleague/s
- ☐ Teammate/s
- ☐ Club Member/s
- ☐ Professional Association Member/s
- ☐ Committee Member/s
- ☐ Religious Organization Member/s
- ☐ Fellow Student/s or Classmate/s (former or current)
- ☐ Leisure Activity Group Member/s
- ☐ Other (Please Specify)

Feel the CLOSEST TO (psychologically / emotionally):

Which relationship type(s) and/or role(s) does this person play in relation to you? (Please select all that apply.)

- ☐ Spouse or Partner
- ☐ Parent/s
- ☐ Grandparent/s
- ☐ Sibling/s
- ☐ Children
- ☐ Grand Children
- ☐ Other Relative/s
- ☐ Close Friend/s
- ☐ Co-worker/s or Colleague/s
- ☐ Teammate/s
- ☐ Club Member/s
- ☐ Professional Association Member/s
- ☐ Committee Member/s
- ☐ Religious Organization Member/s
- ☐ Fellow Student/s or Classmate/s (former or current)
- ☐ Leisure Activity Group Member/s

- Friend/s
- Acquaintance/s
- Other (Please Specify)

Feel the LEAST CLOSE TO (psychologically / emotionally):

Which relationship type(s) and/or role(s) does this person play in relation to you? (Please select all that apply.)

- Spouse or Partner
- Parent/s
- Grandparent/s
- Sibling/s
- Children
- Grand Children
- Other Relative/s
- Close Friend/s
- Friend/s
- Acquaintance/s
- Co-worker/s or Colleague/s
- Teammate/s
- Club Member/s
- Professional Association Member/s
- Committee Member/s
- Religious Organization Member/s
- Fellow Student/s or Classmate/s (former or current)
- Leisure Activity Group Member/s
- Other (Please Specify)

These page timer metrics will not be displayed to the recipient.
First Click: *0 seconds.*
Last Click: *0 seconds.*
Page Submit: *0 seconds.*
Click Count: *0 clicks.*

Block 4.1 Rel Skip

Please select each set of initials you entered once (avoiding duplicates):

- ${q://QID81/ChoiceTextEntryValue}
- ${q://QID82/ChoiceTextEntryValue}
- ${q://QID83/ChoiceTextEntryValue}
- ${q://QID84/ChoiceTextEntryValue}
- I do not have anyone in my relational circle

These page timer metrics will not be displayed to the recipient.
First Click: *0 seconds.*
Last Click: *0 seconds.*
Page Submit: *0 seconds.*
Click Count: *0 clicks.*

Block 4.2

Please answer the questions below regarding the members of your RELATIONAL circle you specified on the previous page.

How often do you have contact (face-to-face, telephone, written, online or via a monitor screen) with _____ ?

	Very Rarely	Rarely	Sometimes	Often	Very Often
» ${q://QID81/ChoiceTextEntryValue}	○	○	○	○	○
» ${q://QID82/ChoiceTextEntryValue}	○	○	○	○	○
» ${q://QID83/ChoiceTextEntryValue}	○	○	○	○	○
» ${q://QID84/ChoiceTextEntryValue}	○	○	○	○	○
» I do not have anyone in my relational circle	○	○	○	○	○

Considering your amount of contact with _____, would you prefer to have:

	Much less contact	Less contact	The same level of contact	More contact	Much more contact
» ${q://QID81/ChoiceTextEntryValue}	○	○	○	○	○
» ${q://QID82/ChoiceTextEntryValue}	○	○	○	○	○
» ${q://QID83/ChoiceTextEntryValue}	○	○	○	○	○
» ${q://QID84/ChoiceTextEntryValue}	○	○	○	○	○
» I do not have anyone in my relational circle	○	○	○	○	○

Please select the picture below that best describes your relationship with _________.

» ${q://QID81/ChoiceTextEntryValue}

» ${q://QID82/ChoiceTextEntryValue}

» ${q://QID83/ChoiceTextEntryValue}

» ${q://QID84/ChoiceTextEntryValue}

» I do not have anyone in my relational circle

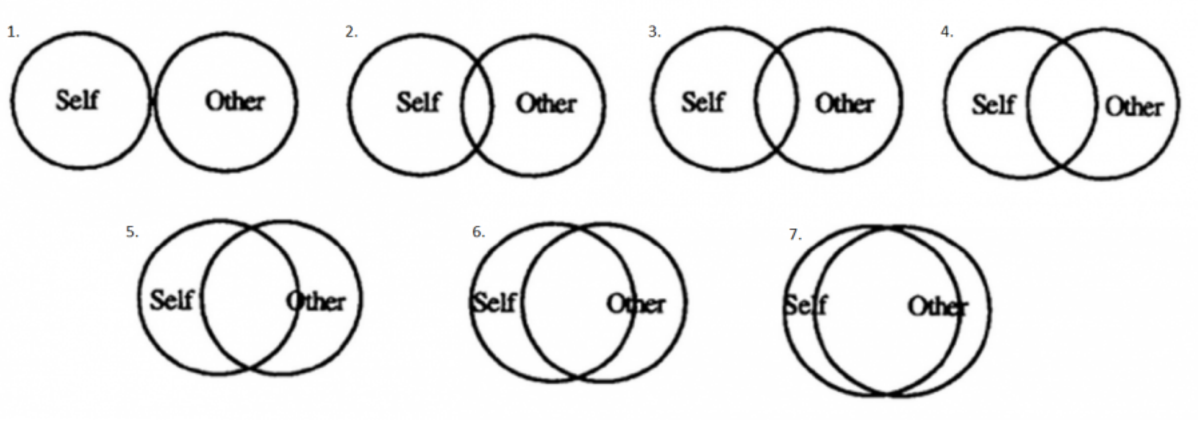

How close (psychologically / emotionally) do you feel to _____?

	Least Close	Closest
» ${q://QID81/ChoiceTextEntryValue}		
» ${q://QID82/ChoiceTextEntryValue}		
» ${q://QID83/ChoiceTextEntryValue}		
» ${q://QID84/ChoiceTextEntryValue}		
» I do not have anyone in my relational circle		

Considering your level of closeness to _______, would you prefer to have:

	Much less closeness	Less closeness	About the same closeness	More closeness	Much more closeness
» ${q://QID81/ChoiceTextEntryValue}	○	○	○	○	○
» ${q://QID82/ChoiceTextEntryValue}	○	○	○	○	○
» ${q://QID83/ChoiceTextEntryValue}	○	○	○	○	○
» ${q://QID84/ChoiceTextEntryValue}	○	○	○	○	○
» I do not have anyone in my relational circle	○	○	○	○	○

These page timer metrics will not be displayed to the recipient.

First Click: *0 seconds.*

Last Click: *0 seconds.*

Page Submit: *0 seconds.*

Click Count: *0 clicks.*

Block 4.3

If an average member of your RELATIONAL circle were to be sitting or standing next to you, how close would that individual have to get to make you feel uncomfortable?

- No distance would make me feel uncomfortable
- 1-9 inches would begin to feel uncomfortable
- 10-25 inches would begin to feel uncomfortable
- 2.5 feet would begin to feel uncomfortable
- 5 feet would begin to feel uncomfortable
- More than 5 feet would begin to feel uncomfortable
- I have no one in my relational circle
- Prefer not to answer

Please answer again using a different scale:

If an average member of your RELATIONAL circle were to be sitting or standing next to you, how close would that individual have to get for yo to feel uncomfortable?

Touching > 5 feet away (>

Select a Distance:

Research regarding human social networks can certainly increase knowledge, but it may also lead to better societal decisions influencing how we relate to one other. This is why we are interested in obtaining information about how you think about your social network. However, research involving human subjects can sometimes be hindered by bad data. Thus, we want to make sure that participants read questions and directions carefully. Please ignore the question below and only select "Ambivalence" for your answer. We realize that this survey can be somewhat repetitive at times, but we greatly appreciate your continued attention. You are well over half way finished.

Please select below the most frequent emotions you have felt in the past week, in reaction to members of your RELATIONAL circle:

- Fear
- Anger
- Sadness
- Joy
- Ambivalence
- Trust
- Anticipation
- Surprise

These page timer metrics will not be displayed to the recipient.

First Click: *0 seconds.*

Last Click: *0 seconds.*

Page Submit: *0 seconds.*

Click Count: *0 clicks.*

Block 4.7

If ______ were to be sitting or standing next to you, how close would ______ have to get to make you feel uncomfortable?

	No distance would make me feel uncomfortable	1-9 inches would begin to feel uncomfortable	10-25 inches would begin to feel uncomfortable	2.5 feet would begin to feel uncomfortable	5 feet would begin to feel uncomfortable	More than 5 feet would begin to feel uncomfortable	I have no one in my relational circle	Prefer not to answe
» ${q://QID81/ChoiceTextEntryValue}	○	○	○	○	○	○	○	○
» ${q://QID82/ChoiceTextEntryValue}	○	○	○	○	○	○	○	○
» ${q://QID83/ChoiceTextEntryValue}	○	○	○	○	○	○	○	○
» ${q://QID84/ChoiceTextEntryValue}	○	○	○	○	○	○	○	○

» I do not have anyone in my relational circle	○	○	○	○	○	○	○	○

Please answer again using a different scale:

If ______ were to be sitting or standing next to you, how close would ______ have to get to make you feel uncomfortable?

	Touching	> 5 feet away (>
» ${q://QID81/ChoiceTextEntryValue}		
» ${q://QID82/ChoiceTextEntryValue}		
» ${q://QID83/ChoiceTextEntryValue}		
» ${q://QID84/ChoiceTextEntryValue}		
» I do not have anyone in my relational circle		

These page timer metrics will not be displayed to the recipient.
First Click: *0 seconds.*
Last Click: *0 seconds.*
Page Submit: *0 seconds.*
Click Count: *0 clicks.*

Main Survey - Block 5

QUESTIONS ABOUT YOUR COLLECTIVE CIRCLE

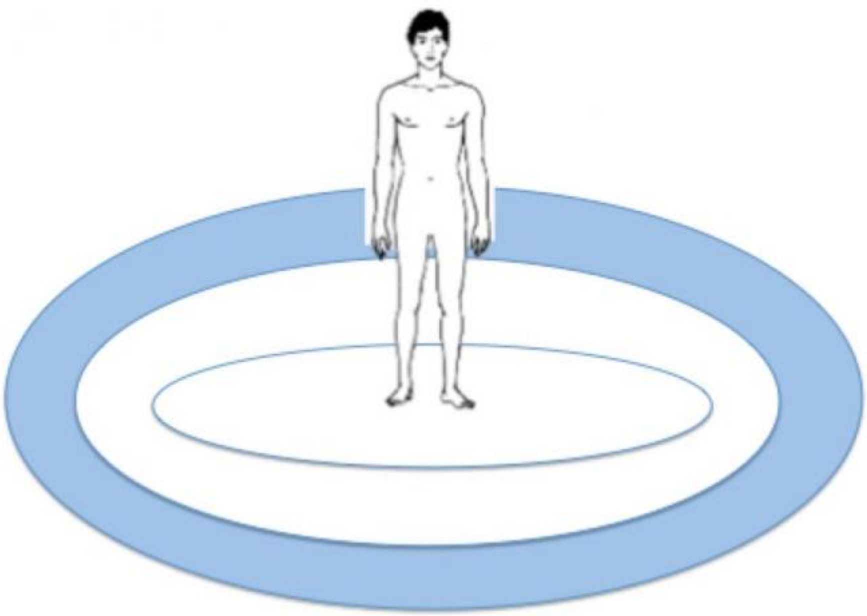

As a reminder, the collective circle generally includes groups or collectives with which you identify, such as sports teams, occupational associations, or religious groups. Other examples might include members of a workplace, a project team, or an academic program, for example. In general, your COLLECTIVE circle is composed of any organizations which help you to form a shared

group identity, or a "social identity."

These page timer metrics will not be displayed to the recipient.
First Click: *0 seconds.*
Last Click: *0 seconds.*
Page Submit: *0 seconds.*
Click Count: *0 clicks.*

Please consider these questions regarding the *groups and collectives* of your COLLECTIVE circle.

How many collectives or groups (teams, clubs, professional associations, etc.) do you associate with regularly?

Which categories would these groups best fit into?

- Sport Team/s
- Club/s
- Professional Association/s
- Committee/s
- Religious Organization/s
- Academic Institution/s or Student Organization/s
- Leisure Activity Group/s
- Other (Please Specify)

How involved are you in your collectives and groups on average?

Involved very little	Involved a little bit	Somewhat involved	Fairly involved	Very involved
○	○	○	○	○

Considering your level of involvement, would you like to be

Much less involved	Less involved	Involved the same amount	More involved	Much more involved
○	○	○	○	○

These page timer metrics will not be displayed to the recipient.
First Click: *0 seconds.*
Last Click: *0 seconds.*
Page Submit: *0 seconds.*
Click Count: *0 clicks.*

Please select the picture below that best describes your relationship with your collectives and groups on average:

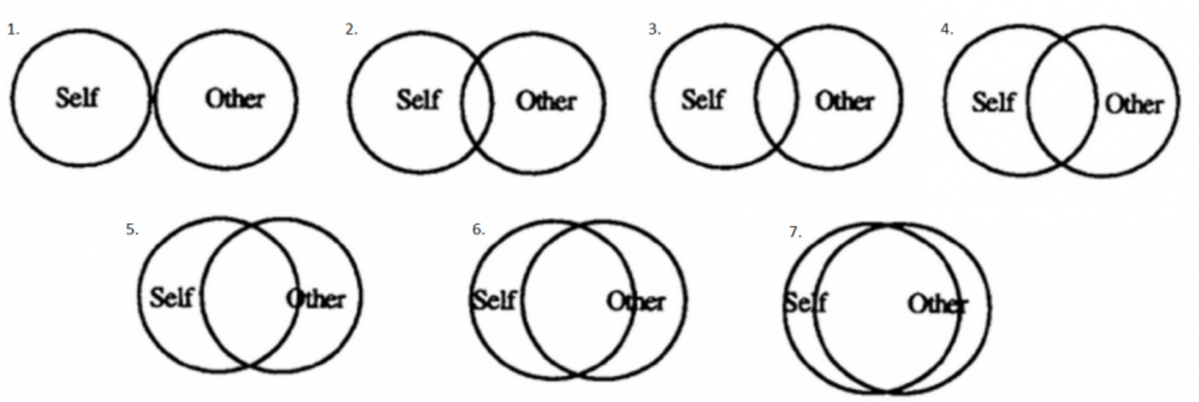

How close (psychologically / emotionally) do you feel to your groups or collectives on average?

Least Close Close

Rate your closeness:	

Which person/s would most likely be a member/s of the collective circle?

- A stranger I pass on the street
- The love of my life
- My good friend
- My teammate

Considering your level of closeness to your COLLECTIVE circle, would you prefer to have:

Much less closeness	Less closeness	About the same closeness	More closeness	Much more closeness
○	○	○	○	○

How much would you say you personally identify with your groups or collectives (how important are they for forming your "social identity")?

Very little	A little	Somewhat	A fair amount	Very much
○	○	○	○	○

Based on your level of identification, would you prefer to identify with your groups and collectives ______?

Much less	Less	About the same	More	Much more
○	○	○	○	○

These page timer metrics will not be displayed to the recipient.
First Click: *0 seconds.*
Last Click: *0 seconds.*
Page Submit: *0 seconds.*
Click Count: *0 clicks.*

Main Survey - Block 6

Now, please consider these questions regarding the *individual members* of your groups and collectives.

How many *individual members* of your collectives or groups (teams, clubs, professional associations, etc.) do you associate with on a regular basis? (Please do not include any individuals who might better fit within your relational circle, such as friends who you would rely on for mutual protection and assistance.)

Which relationship type(s) and/or role(s) do the *individual members* of your COLLECTIVE circle play in relation to you? (Please select all that apply.)

- Spouse or Partner
- Parent/s
- Grandparent/s
- Sibling/s
- Children
- Grand Children
- Other Relative/s
- Close Friend/s
- Friend/s
- Acquaintance/s
- Co-worker/s or Colleague/s
- Teammate/s
- Club Member/s
- Professional Association Member/s
- Committee Member/s
- Religious Organization Member/s
- Fellow Student/s or Classmate/s (former or current)
- Leisure Activity Group Member/s
- Other (Please Specify)

Please write the initials of an *individual member* of your COLLECTIVE circle who you
[Please note: these should NOT be people who you would include in your intimate or relational circles. Also, feel free to respond with the same initials in more than one blank--for example, if the person you have the most frequent contact is also the person you feel closest to. These initials will not be used for any identification purposes and are completely confidential. They will only be used to refer to specific people in late questions.]

Have the MOST FREQUENT CONTACT WITH (face-to-face, telephone, written, online or via a monitor screen):

Which relationship type(s) and/or role(s) does this person play in relation to you? (Please select all that apply.)

- Spouse or Partner
- Parent/s
- Grandparent/s
- Sibling/s
- Children
- Grand Children
- Other Relative/s
- Close Friend/s
- Friend/s
- Acquaintance/s
- Co-worker/s or Colleague/s
- Teammate/s
- Club Member/s
- Professional Association Member/s
- Committee Member/s
- Religious Organization Member/s
- Fellow Student/s or Classmate/s (former or current)
- Leisure Activity Group Member/s
- Other (Please Specify)

Have the LEAST FREQUENT CONTACT WITH (face-to-face, telephone, written, online or via a monitor screen):

Which relationship type(s) and/or role(s) does this person play in relation to you? (Please select all that apply.)

- Spouse or Partner
- Parent/s
- Grandparent/s
- Sibling/s
- Children
- Grand Children
- Other Relative/s
- Close Friend/s
- Friend/s
- Acquaintance/s
- Co-worker/s or Colleague/s
- Teammate/s
- Club Member/s
- Professional Association Member/s
- Committee Member/s
- Religious Organization Member/s
- Fellow Student/s or Classmate/s (former or current)
- Leisure Activity Group Member/s
- Other (Please Specify)

Feel the CLOSEST TO (psychologically / emotionally):

Which relationship type(s) and/or role(s) does this person play in relation to you? (Please select all that apply.)

- Spouse or Partner
- Parent/s
- Grandparent/s
- Sibling/s
- Children
- Grand Children
- Other Relative/s
- Close Friend/s
- Friend/s
- Acquaintance/s
- Co-worker/s or Colleague/s
- Teammate/s
- Club Member/s
- Professional Association Member/s
- Committee Member/s
- Religious Organization Member/s
- Fellow Student/s or Classmate/s (former or current)
- Leisure Activity Group Member/s
- Other (Please Specify)

Feel the LEAST CLOSE TO (psychologically / emotionally):

Which relationship type(s) and/or role(s) does this person play in relation to you? (Please select all that apply.)

- ☐ Spouse or Partner
- ☐ Parent/s
- ☐ Grandparent/s
- ☐ Sibling/s
- ☐ Children
- ☐ Grand Children
- ☐ Other Relative/s
- ☐ Close Friend/s
- ☐ Friend/s
- ☐ Acquaintance/s
- ☐ Co-worker/s or Colleague/s
- ☐ Teammate/s
- ☐ Club Member/s
- ☐ Professional Association Member/s
- ☐ Committee Member/s
- ☐ Religious Organization Member/s
- ☐ Fellow Student/s or Classmate/s (former or current)
- ☐ Leisure Activity Group Member/s
- ☐ Other (Please Specify) ________

These page timer metrics will not be displayed to the recipient.
First Click: *0 seconds.*
Last Click: *0 seconds.*
Page Submit: *0 seconds.*
Click Count: *0 clicks.*

6.1 Col Skip

Please select each set of initials you entered once (avoiding duplicates):

- ☐ ${q://QID122/ChoiceTextEntryValue}
- ☐ ${q://QID123/ChoiceTextEntryValue}
- ☐ ${q://QID124/ChoiceTextEntryValue}
- ☐ ${q://QID125/ChoiceTextEntryValue}
- ☐ I do not have anyone in my collective circle

These page timer metrics will not be displayed to the recipient.
First Click: *0 seconds.*
Last Click: *0 seconds.*
Page Submit: *0 seconds.*
Click Count: *0 clicks.*

6.2

Please answer the questions below regarding the *individual members* of your COLLECTIVE circle you specified on the previous page.

How often do you have contact (face-to-face, telephone, written, online or via a monitor screen) with _____ ?

	Very Rarely	Rarely	Sometimes	Often	Very Often
» ${q://QID122/ChoiceTextEntryValue}	○	○	○	○	○
» ${q://QID123/ChoiceTextEntryValue}	○	○	○	○	○
» ${q://QID124/ChoiceTextEntryValue}	○	○	○	○	○
» ${q://QID125/ChoiceTextEntryValue}	○	○	○	○	○
» I do not have anyone in my collective circle	○	○	○	○	○

Considering your amount of contact with _____, would you prefer to have:

	Much less contact	Less contact	The same level of contact	More contact	Much more contact
» ${q://QID122/ChoiceTextEntryValue}	○	○	○	○	○
» ${q://QID123/ChoiceTextEntryValue}	○	○	○	○	○
» ${q://QID124/ChoiceTextEntryValue}	○	○	○	○	○
» ${q://QID125/ChoiceTextEntryValue}	○	○	○	○	○

» I do not have anyone in my collective circle					

Please select the picture below that best describes your relationship with _________.

Members of your collective or groups on average
» ${q://QID122/ChoiceTextEntryValue}
» ${q://QID123/ChoiceTextEntryValue}
» ${q://QID124/ChoiceTextEntryValue}
» ${q://QID125/ChoiceTextEntryValue}
» I do not have anyone in my collective circle

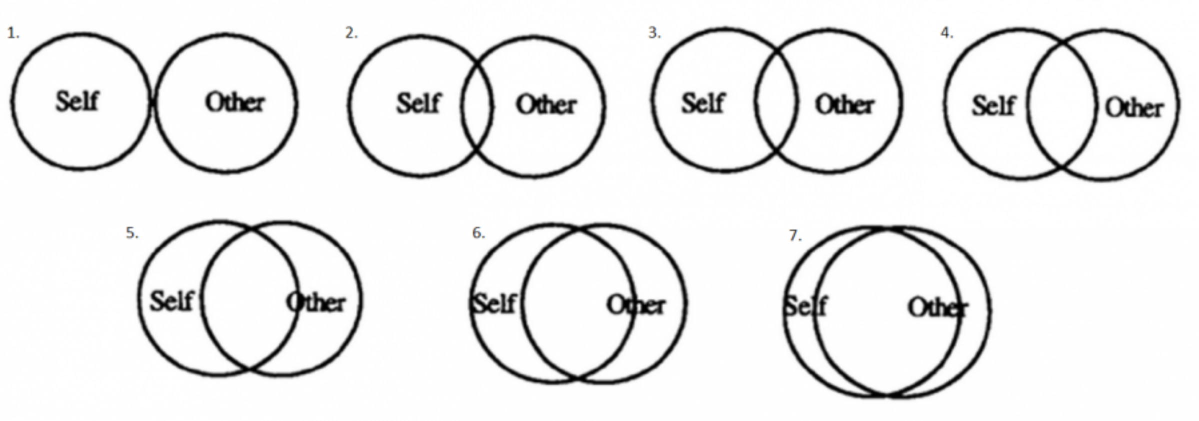

How close (psychologically / emotionally) do you feel to _____?

Least Close Close

Members of your collectives or groups on average:	
» ${q://QID122/ChoiceTextEntryValue}	
» ${q://QID123/ChoiceTextEntryValue}	
» ${q://QID124/ChoiceTextEntryValue}	
» ${q://QID125/ChoiceTextEntryValue}	
» I do not have anyone in my collective circle	

Considering your level of closeness to _______, would you prefer to have:

	Much less closeness	Less closeness	About the same closeness	More closeness	Much more closeness
Members of your collectives or groups on average:					
» ${q://QID122/ChoiceTextEntryValue}					
» ${q://QID123/ChoiceTextEntryValue}					
» ${q://QID124/ChoiceTextEntryValue}					
» ${q://QID125/ChoiceTextEntryValue}					
» I do not have anyone in my collective circle					

These page timer metrics will not be displayed to the recipient.
First Click: *0 seconds.*
Last Click: *0 seconds.*
Page Submit: *0 seconds.*
Click Count: *0 clicks.*

Block 6.3

If a member of your COLLECTIVE circle were to be sitting or standing next to you, how close would that individual have to get to make you feel uncomfortable?

- No distance would make me feel uncomfortable
- 1-9 inches would begin to feel uncomfortable
- 10-25 inches would begin to feel uncomfortable
- 2.5 feet would begin to feel uncomfortable
- 5 feet would begin to feel uncomfortable
- More than 5 feet would begin to feel uncomfortable
- I have no one in my collective circle
- Prefer not to answer

Please answer again using a different scale:

If a member of your COLLECTIVE circle were to be sitting or standing next to you, how close would that individual have to get for you to feel uncomfortable?

Touching > 5 feet away (>

Select a Distance:

These page timer metrics will not be displayed to the recipient.

First Click: *0 seconds.*

Last Click: *0 seconds.*

Page Submit: *0 seconds.*

Click Count: *0 clicks.*

Block 6.5

If ______ were to be sitting or standing next to you, how close would ______ have to get to make you feel uncomfortable?

	No distance would make me feel uncomfortable	1-9 inches would begin to feel uncomfortable	10-25 inches would begin to feel uncomfortable	2.5 feet would begin to feel uncomfortable	5 feet would begin to feel uncomfortable	More than 5 feet would begin to feel uncomfortable	I have no one in my collective circle	Prefe not to answe
» ${q://QID122/ChoiceTextEntryValue}								
» ${q://QID123/ChoiceTextEntryValue}								
» ${q://QID124/ChoiceTextEntryValue}								
» ${q://QID125/ChoiceTextEntryValue}								
» I do not have anyone in my collective circle								

Please answer again using a different scale:

If ______ were to be sitting or standing next to you, how close would ______ have to get to make you feel uncomfortable?

Touching > 5 feet (> 5

» ${q://QID122/ChoiceTextEntryValue}	
» ${q://QID123/ChoiceTextEntryValue}	
» ${q://QID124/ChoiceTextEntryValue}	

» ${q://QID125/ChoiceTextEntryValue}	
» I do not have anyone in my collective circle	

The collective circle often fulfills a sense of group belonging and identity. Please respond to the statement, the earth is flat:

- True
- False

These page timer metrics will not be displayed to the recipient.

First Click: *0 seconds.*

Last Click: *0 seconds.*

Page Submit: *0 seconds.*

Click Count: *0 clicks.*

Main Survey - Block 7 (Strangers)

QUESTIONS ABOUT YOUR CIRCLE OF STRANGERS (i.e., those you might encounter on a regular day who you would not include in your Intimate, Relational, or Collective circles)

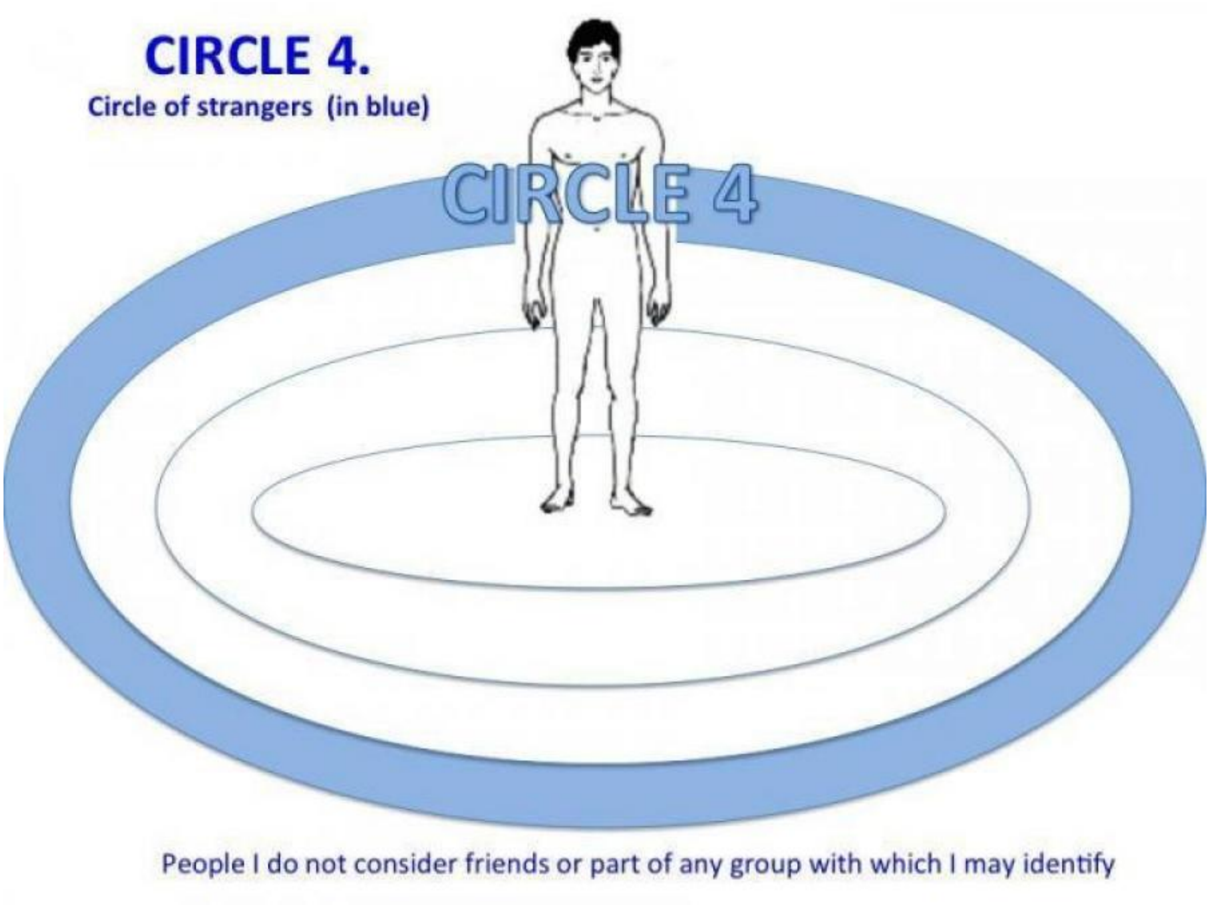

As a reminder, the circle of strangers includes people you might encounter on a normal day who you do not know personally, consider friends, acquaintances, or members of your groups or collectives.

These page timer metrics will not be displayed to the recipient.
First Click: *0 seconds.*
Last Click: *0 seconds.*
Page Submit: *0 seconds.*
Click Count: *0 clicks.*

Block 7.5

How many STRANGERS would you estimate you encounter on a typical day?

If a STRANGER were to be sitting or standing next to you, how close would that individual have to get to make you feel uncomfortable?

- No distance would make me feel uncomfortable
- 1-9 inches would begin to feel uncomfortable
- 10-25 inches would begin to feel uncomfortable
- 2.5 feet would begin to feel uncomfortable
- 5 feet would begin to feel uncomfortable
- More than 5 feet would begin to feel uncomfortable
- I do not encounter any strangers
- Prefer not to answer

Please answer again using a different scale:

If a STRANGER were to be sitting or standing next to you, how close would that individual have to get to make you feel uncomfortable?

Touching > 5 feet away (>

Select a Distance:

Which question were you asked earlier in this survey?

- "During the past week, how often did you feel totally rejected?""
- "During the past week, how often did you feel that there were people you could turn to?"
- "During the past week, how many times did you have a panic attack?"
- During the past week, what was your favorite flavor of ice cream?"

These page timer metrics will not be displayed to the recipient.
First Click: *0 seconds.*
Last Click: *0 seconds.*
Page Submit: *0 seconds.*
Click Count: *0 clicks.*

Main Survey - Block 8 (Mood)

YOU ARE ALMOST DONE!! NOW LET US ASK YOU A FEW QUESTIONS

ABOUT YOUR GENERAL MOOD OVER THE PAST WEEK.

During the past week, how often did you feel tense or wound up?

Not at all	Time to time	A lot of the time	Most of the time
○	○	○	○

During the past week, have you still enjoyed the things you used to enjoy?

Hardly at all	Only a little	Not quite so much	Definitely as much
○	○	○	○

During the past week, did you get a sort of frightened feeling as if something awful was about to happen?

Not at all	A little, but it doesn't worry me	Yes, but not too badly	Very definitely and quite badly
○	○	○	○

During the past week, did you laugh and see the funny side of things?

Not at all	Definitely not so much now	Not quite as much now	As much as I always could
○	○	○	○

During the past week, did you have worrying thoughts going through your mind?

A great deal of time	A lot of time	From time to time but not too often	Only occasionally
○	○	○	○

During the past week, did you feel cheerful?

Not at all	Not often	Sometimes	Most of the time
○	○	○	○

During the past week, how often did you feel a sort of nervous energy? Select sometimes.

Not at all	Not often	Sometimes	Most of the time
○	○	○	○

These page timer metrics will not be displayed to the recipient.
First Click: *0 seconds.*
Last Click: *0 seconds.*
Page Submit: *0 seconds.*
Click Count: *0 clicks.*

During the past week, were you able to sit at ease and feel relaxed?

Definitely	Usually	Not often	Not at all
○	○	○	○

During the past week, did you feel as if you were slowed down?

Nearly all the time	Very often	Sometimes	Not at all
○	○	○	○

During the past week, did you get a sort of frightened feeling like butterflies in the stomach?

Not at all	Occasionally	Quite often	Very often
○	○	○	○

During the past week, have you lost interest in your appearance?

Definitely	I don't take so much care as I should	I may not take quite as much care	I take just as much care as ever
○	○	○	○

During the past week, have you felt restless as if you had to be on the move?

Not at all	Not very much	Quite a lot	Very much indeed
○	○	○	○

During the past week, have you looked forward with enjoyment to things?

As much as I ever did	Rather less than I used to	Definitely less than I used to	Hardly at all
○	○	○	○

During the past week, did you get sudden feelings of panic?

Not at all	Not very often	Quite often	Very often indeed
○	○	○	○

During the past week, were you able to enjoy a good book or radio or TV program?

Often	Sometimes	Not often	Very seldom
○	○	○	○

These page timer metrics will not be displayed to the recipient.
First Click: *0 seconds.*
Last Click: *0 seconds.*
Page Submit: *0 seconds.*
Click Count: *0 clicks.*

Ending and Feedback

Thank you for your time! We greatly appreciate your help with this survey. Please click through to the next page for your mTurk code number.

Optional: please feel free to leave any feedback you have regarding this survey (impressions, insights, problems you encountered, etc.). We greatly value your feedback, and these will be read by the primary investigator.

These page timer metrics will not be displayed to the recipient.
First Click: *0 seconds.*
Last Click: *0 seconds.*
Page Submit: *0 seconds.*
Click Count: *0 clicks.*
